# Cryptic diversity, polyploidy and intraspecific divergence in *Erysimum* (Brassicaceae) species endemic to the western Balkans

**DOI:** 10.1093/aob/mcag229

**Published:** 2026-07-30

**Authors:** Richard Bačák, Marek Šlenker, Hana Majerová, Barbora Šingliarová, Adam Kantor, Katarína Skokanová, Ingrid Turisová, Peter Turis, Terezie Mandáková, Judita Zozomová-Lihová

**Affiliations:** Institute of Botany, Plant Science and Biodiversity Centre, Slovak Academy of Sciences, SK-845 23 Bratislava, Slovakia; Institute of Botany, Plant Science and Biodiversity Centre, Slovak Academy of Sciences, SK-845 23 Bratislava, Slovakia; Institute of Botany, Plant Science and Biodiversity Centre, Slovak Academy of Sciences, SK-845 23 Bratislava, Slovakia; Institute of Botany, Plant Science and Biodiversity Centre, Slovak Academy of Sciences, SK-845 23 Bratislava, Slovakia; Institute of Botany, Plant Science and Biodiversity Centre, Slovak Academy of Sciences, SK-845 23 Bratislava, Slovakia; Institute of Botany, Plant Science and Biodiversity Centre, Slovak Academy of Sciences, SK-845 23 Bratislava, Slovakia; Department of Biology and Environmental Studies, Faculty of Natural Sciences, Matej Bel University, SK-974 01 Banská Bystrica, Slovakia; Department of Biology and Environmental Studies, Faculty of Natural Sciences, Matej Bel University, SK-974 01 Banská Bystrica, Slovakia; Central European Institute of Technology, Masaryk University, CZ-625 00 Brno, Czechia; Department of Experimental Biology, Faculty of Science, Masaryk University, CZ-625 00 Brno, Czechia; Institute of Botany, Plant Science and Biodiversity Centre, Slovak Academy of Sciences, SK-845 23 Bratislava, Slovakia

**Keywords:** Balkan Peninsula, cryptic diversity, dysploidy, endemics, *Erysimum*, introgression, polyploidy, reticulate evolution

## Abstract

**Background and Aims:**

Several evolutionary drivers have been proposed to explain the exceptional species and genetic diversity of the Balkan flora, including the moderate impact of glaciations enabling long-term *in situ* persistence, ecological and topographic heterogeneity promoting vicariant differentiation, and polyploidy. Despite these shared drivers, plant lineages often show contrasting evolutionary histories, suggesting lineage-specific rather than broadly generalizable responses. The genus *Erysimum*, notable for its high endemism, karyological variation, recent and rapid speciation, and signatures of reticulate evolution, provides an excellent model for exploring this diversity of evolutionary trajectories and speciation drivers in the Balkans.

**Methods:**

Focusing on five Balkan endemics of the *E. odoratum* species complex sampled from 47 populations, we examined chromosomal and genome size variation and applied two complementary genomic approaches, target enrichment (Hyb-Seq) and RADseq, along with morphometric analyses. These datasets were used to circumscribe species, uncover intraspecific variation patterns and assess the extent and evolutionary consequences of interspecific gene flow and polyploidy.

**Key Results:**

Genetic and morphological evidence was largely congruent, clarifying species boundaries but also revealing cryptic diversity, leading to the recognition of eight entities. Most diversity was concentrated in the southern Dinarides, where we detected multiple cytotypes, an undescribed lineage, a polyphyletic species resulting from two independent autopolyploidization events, and allotetraploids arising in a contact zone. Geographical distance and biogeographical barriers across the mountainous Balkan landscape are likely to have shaped patterns of divergence and promoted speciation. Discrepancies between chromosome numbers and genome size indicate dynamic chromosomal evolution.

**Conclusions:**

Vicariant differentiation, auto- and allopolyploidy, genome size and chromosomal dynamics together account for the pronounced diversity of *Erysimum* in the Balkans, illustrating how speciation is driven within this biodiversity hotspot. Importantly, substantial evolutionary potential is retained within species, because the coexistence of multiple intraspecific genetic lineages and cytotypes can reinforce reproductive barriers and promote future diversification.

## INTRODUCTION

The Balkan Peninsula is one of the major biodiversity centres in Europe (Griffiths *et al.*, 2004; Nieto Feliner, 2014), with its central and southern regions particularly notable for high species richness and endemism (Stevanović *et al.*, 2007; Georghiou and Delipetrou, 2010; Lubarda *et al.*, 2014; Tomović *et al.*, 2014). Phylogeographical studies likewise revealed greater genetic diversity in the southern than in the northern regions of the peninsula (Španiel and Rešetnik, 2022). This concentration of species and genetic diversity appears to reflect the interplay between the complex palaeogeographical history and heterogeneous environmental conditions in the Balkans (Médail and Diadema, 2009; Nieto Feliner, 2014; Thompson, 2020). The relatively moderate impact of Pleistocene glaciations (Hughes *et al.*, 2006) contributed to long-term environmental stability across much of the area, enabling the persistence of many plant lineages *in situ* (Hewitt, 1996, 2011; Nieto Feliner, 2011). At the same time, pronounced topographic complexity, such as high mountain ridges and deep river gorges, especially in the central Balkans, promoted population isolation and vicariant differentiation (Španiel and Rešetnik, 2022). Polyploidization, a major driver of angiosperm diversification, has also been shown to be common and recurrent in the Balkans (Marques *et al.*, 2018, see e.g. Niketić *et al.*, 2022; Rešetnik *et al.*, 2023; Đurović *et al*., 2024; Šlenker *et al.*, 2024; Berisha *et al.*, 2025). Despite these shared evolutionary drivers, phylogenetic and phylogeographical studies consistently reveal considerable heterogeneity among taxa, underscoring that no single evolutionary scenario applies uniformly across different plant groups (Španiel and Rešetnik, 2022). Within this context, the genus *Erysimum* (Brassicaceae) provides an excellent system to study the mosaic of diverse evolutionary trajectories in the Balkan flora. This genus comprises >200 species distributed mainly in Eurasia, with its centre of diversity extending from the Mediterranean Basin to Central Asia (German and Al-Shehbaz, 2008; Moazzeni *et al.*, 2014; German *et al.*, 2023). Only a few *Erysimum* species occupy broad geographical ranges, whereas the majority are narrow endemics (Jalas and Suominen, 1994; Polatschek, 2013; Moazzeni *et al.*, 2014; Czarna *et al.*, 2016). In the Balkan Peninsula, ∼30 mainland species (excluding several Aegean endemics) are currently recognized (Jalas and Suominen, 1994), yet their phylogenetic relationships and evolutionary history remain unexplored.

Genus-level phylogenetic studies indicate that *Erysimum* underwent a recent and rapid radiation, with speciation beginning in the late Pliocene to early Pleistocene (5.2–2.3 Mya). The diversification of European clades occurred later, primarily during the early to middle Pleistocene (Moazzeni *et al.*, 2014; Züst *et al*., 2020). Pronounced phylogenetic discordance has been attributed to extensive incomplete lineage sorting and reticulate evolutionary processes, which are common during recent species radiations (Züst *et al*., 2020). Previous studies of Iberian and Carpathian species further suggest multiple allopolyploidization events and signatures of introgression even among diploids, patterns consistent with weak prezygotic post-pollination barriers documented in several Iberian species (Osuna-Mascaró, 2020; Osuna-Mascaró *et al.*, 2022, 2023; Bačák *et al.*, 2026). Although traditional taxonomic treatments grouped some species into complexes based on morphological resemblance, growth form, life cycles and biogeographical patterns (Ball *et al.*, 1993; Jalas and Suominen, 1994; Polatschek, 2013; Abdelaziz *et al.*, 2014; Peccenini and Polatschek, 2016), molecular evidence demonstrates that these assemblages do not necessarily reflect natural evolutionary lineages (Abdelaziz *et al.*, 2014; Bačák *et al.*, 2026). Nevertheless, published phylogenies consistently reveal a strong geographical signal, suggesting that spatial separation has been one of major drivers of genus diversification (Moazzeni *et al.*, 2014; Züst *et al*., 2020; Bačák *et al.*, 2026). Rapid radiation in response to allopatric or ecological isolation is also thought to have generated cryptic species (Abdelaziz *et al.*, 2011; Czarna *et al.*, 2016). Furthermore, *Erysimum* exhibits remarkable karyological diversity, encompassing multiple base chromosome numbers (*x* = 6–17) and ploidy levels, with chromosome numbers ranging from 2*n* = 12 to 2*n* ≈ 90 (Warwick and Al-Shehbaz, 2006; Polatschek, 2013; Kiefer *et al.*, 2014).

Our recent study of the *Erysimum odoratum* complex, comprising Balkan and Carpathian biennial species, demonstrated its polyphyletic nature (Bačák *et al.*, 2026). Despite morphological similarity, Balkan and Carpathian species formed distinct clades. Carpathian diploids were proposed to represent a single species, *Erysimum witmannii* Zaw., within which biogeographical barriers and cytotype variation have driven population differentiation. However, this differentiation did not align with morphological patterns and contradicted earlier taxonomic treatments that recognized up to four diploid species in the Carpathians (Jalas and Suominen, 1994; Kliment, 1999; Michalková, 1999). The more broadly distributed *Erysimum odoratum* Ehrh., ranging from north-eastern France to eastern Ukraine and extending south into the northern Balkans (Jalas and Suominen, 1994; Polatschek, 2013), was found to consist of sibling polyploids that had arisen independently (Bačák *et al.*, 2026).

In contrast, five Balkan species traditionally assigned to the *E. odoratum* complex were previously addressed only marginally (Bačák *et al.*, 2026). These species occur in the western Balkans and Peloponnese, where they inhabit screes, rock crevices, xerothermic rocky grasslands and forest edges on limestone, in addition to ultramafic and silicate substrates (Jalas and Suominen, 1994; Polatschek, 2013). They include *Erysimum carniolicum* Dolliner (2*n* = 14; Slovenia, Croatia, Bosnia and Herzegovina, south-western Serbia and Montenegro), *Erysimum kuemmerlei* Jáv. (2*n* = 14; Kosovo, North Macedonia and Albania) and *Erysimum pectinatum* Bory & Chaub. (2*n* = 12, 14; Peloponnese), all three occurring in the montane to (sub)alpine belt. Two additional southern Dinaric endemics were described more recently (Polatschek, 2013), both growing from coastal to subalpine habitats: *Erysimum vitekii* Polatschek (2*n* = 12; southern Bosnia and Herzegovina, southern Croatia, Montenegro and northern Albania) and *Erysimum croaticum* Polatschek (2*n* = 42; southern Croatia and Kosovo). Several taxonomic and evolutionary challenges surround these taxa. Despite their disjunct occurrence, *E. kuemmerlei* and *E. pectinatum* show striking morphological resemblance, particularly in their pinnatifid to pinnatisect stem leaves. They were even synonymized in the past (Josifović *et al.*, 1972; Vangjeli, 2017), or it was suggested that *E. kuemmerlei* might be morphologically intermediate between *E. carniolicum* and *E. pectinatum* (Jalas and Suominen, 1994). Ambiguous morphological distinction between *E. carniolicum* and *E. odoratum* has also been reported from western Serbia and Montenegro (Jalas and Suominen, 1994). Furthermore, when describing *E. croaticum*, Polatschek (2013) noted morphological differentiation between populations from the Adriatic coast in southern Croatia and those from Kosovo but suggested that these differences did not justify separate taxonomic recognition. The presence of two diploid cytotypes, 2*n* = 12 and 2*n* = 14 (see above) indicated two basic chromosome numbers (*x* = 6 and *x* = 7) in these western Balkan species (Polatschek, 2013). In addition, recent findings revealed remarkable variation in genome size and the presence of another cytotype (2*n* = 16), although based on limited sampling (Bačák *et al.*, 2026). Overall, the previous findings in the *E. odoratum* complex highlight the need for an integrative approach that evaluates patterns of variations from multiple comparative perspectives (Dayrat, 2005). Focusing on the Balkan lineage traditionally assigned to this species complex, and in line with the unified species concept (de Queiroz, 2007; Karbstein *et al.*, 2024), our aims were as follows: (1) to clarify species circumscription and assess species divergence by integrating genomic, cytotype, morphological and geographical evidence; (2) to characterize genome size and cytotype variation within species and test whether these patterns correspond to genetic structure or geographic distribution; (3) to assess the extent and evolutionary significance of interspecific gene flow, both within the target group and with sympatric or parapatric congeners; and (4) to evaluate specifically whether disjunctly distributed entities (such as *E. kuemmerlei* vs. *E. pectinatum*, or *E. croaticum* from Adriatic vs. inland regions) exhibit significant divergence to be supported as different species.

## MATERIALS AND METHODS

### Sampling design

This study comprised 47 populations of the target species, i.e. Balkan species traditionally assigned to the *E. odoratum* complex: *E. carniolicum*, *E. kuemmerlei*, *E. pectinatum*, *E. vitekii* and *E. croaticum*, including their type localities (Fig. 1A; Supplementary Data Table S1). Eleven of these populations (one to three per species) were previously sampled and analysed in the study focused on the Carpathians (Bačák *et al.*, 2026), from which the relevant flow cytometric, sequence and chromosome-count data were obtained (for details, see Supplementary Data Table S1). In the present study, we substantially expanded both the sampling and datasets to cover the entire distribution ranges of these Balkan species and to capture potential intraspecific patterns. All 47 populations were subjected to flow cytometry (Doležel *et al.*, 2007*a*) to estimate relative nuclear DNA content, and two next-generation sequencing approaches: Restriction site-Associated DNA sequencing (RADseq) (Andrews *et al.*, 2016), and Hyb-Seq (Weitemier *et al.*, 2014). A subset of populations was sampled for morphometric analyses (30 populations) and determination of the number of chromosomes (31 populations). Chromosomes were counted for each species and each genome size category (cytotype) within species, as inferred from flow cytometry data, to identify all cytotypes present across the sampled populations. Given that *E. croaticum* was found to be divided into two geographically structured phylogenetic clades (see Results), we refer to the species in a broad sense as *E. croaticum* s.l. For the clade including samples from the type locality (Brela: Zadvarje, Croatia), we use the name *E. croaticum*, whereas the clade encompassing samples from Kosovo is referred to as *E. croaticum* auct.

**Fig. 1.**
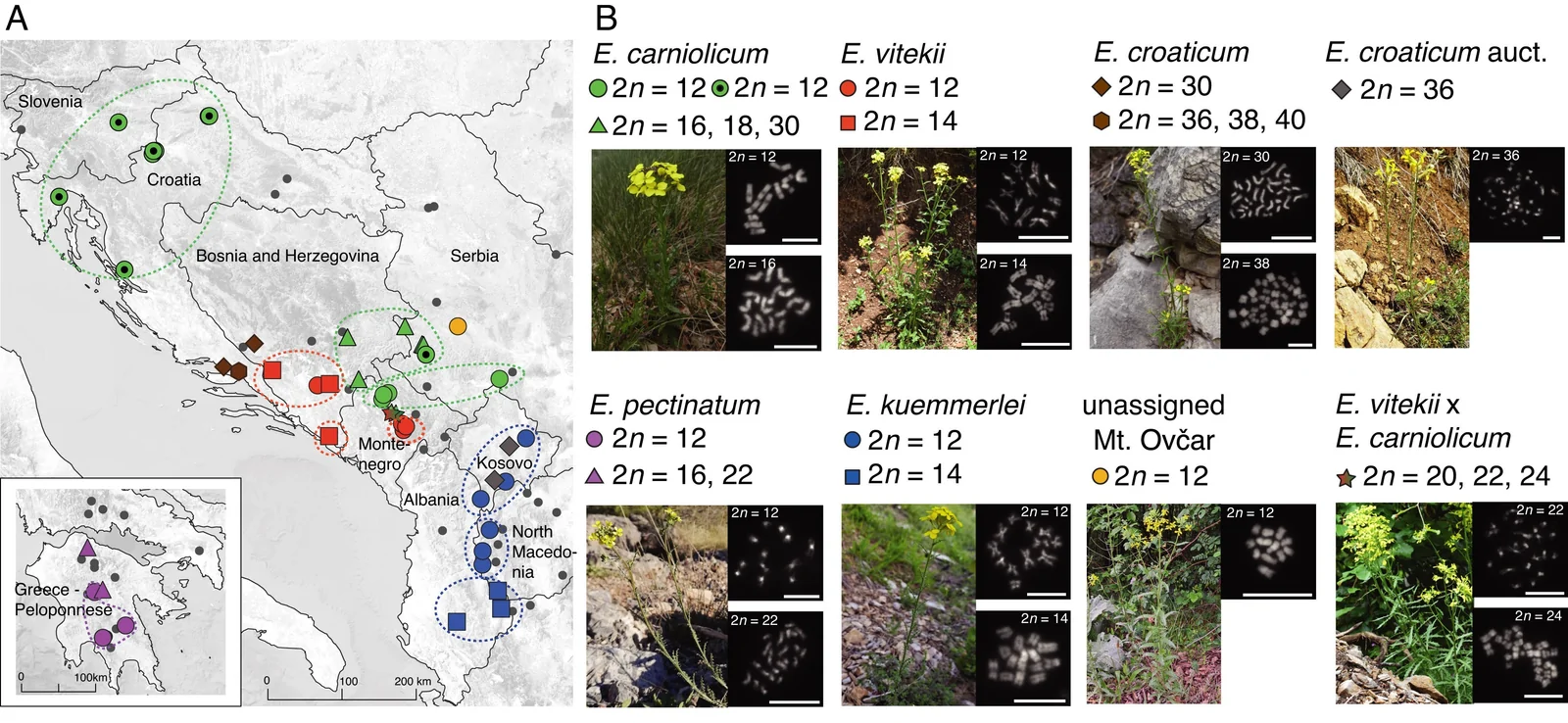
Balkan species of the traditionally circumscribed *Erysimum odoratum* complex and their cytotype variation. (A) Geographical distribution of sampled populations used for flow cytometry, RADseq and Hyb-Seq. A subset of these populations was examined for chromosome number and morphometrics (see Supplementary Data Table S1). Symbols correspond to those in B. Different symbol colours represent different species (or entities), and the symbol shapes distinguish cytotypes within the species. The two circle types used for *E. carniolicum* denote cytotypes that share the same chromosome number (2*n* = 12) but differ in genome size. Coloured dashed outlines in the map indicate genetic clades following Fig. 3 and Supplementary Data Fig. S2. Small grey circles on the map indicate sample sites of additional, non-target species occurring within the mapped area (all listed in Supplementary Data Table S1). (B) Photographs of the target species and mitotic chromosomes from selected accessions. Sample origin (population codes as in Supplementary Data Table S1): *E. carniolicum* (Sadikovac), 2*n* = 12 (Kalnik, chromosome spread prepared from anthers), 2*n* = 16 (MGLC, chromosome spread prepared from root tips); *E. vitekii* (Rilja), 2*n* = 12 (CRKV, root tips), 2*n* = 14 (Podvranić, root tips); *E. pectinatum* (MSVN), 2*n* = 12 (ELAT, anthers), 2*n* = 22 (MSVN, anthers); *E. kuemmerlei* (GJSL), 2*n* = 12 (MVRV, root tips), 2*n* = 14 (GLCA, root tips); *E. croaticum* (OMIS), 2*n* = 30 (OMIS, root tips), 2*n* = 38 (GBRE, root tips); *E. croaticum* auct. (GLSH), 2*n* = 36 (PRIZ, anthers); taxonomically unassigned entity from Mt. Ovčar, 2*n* = 12 (OVCA, root tips); allotetraploids *E. carniolicum* × *E. vitekii* (VRMAC), 2*n* = 22, 24 (VRMAC, anthers). Scale bars: 5 μm. Additional chromosomal plates are shown in Supplementary Data Fig. S1.

To place the target species in a broader phylogenetic context and assess potential gene flow with congeners, we included 26 additional *Erysimum* species represented by 99 populations. Of these, ten species (45 populations) were sampled and analysed by Bačák *et al.* (2026), and we integrated those data into the present analyses. All remaining species and populations were newly sampled and analysed here (for details, see Supplementary Data Table S1). For *E. odoratum* and *E. witmannii*, we selected 21 populations (from the 120 populations examined in detail by Bačák *et al.*, 2026) that capture different cytotypes and geographical regions; for *E. odoratum*, this included particularly the southern margin of its distribution (Croatia, Serbia, Bosnia and Herzegovina), where it overlaps with the ranges of the target Balkan species (Supplementary Data Table S1). Two population samples initially identified as *E. sylvestre* from the south-eastern Alps and Dinarides are treated here as *E. sylvestre* auct., because this species appears to be polyphyletic (R. Bačák, unpublished research). It comprises an Eastern Alpine clade (not sampled in the present study) and a clade distributed in the south-eastern Alps and Dinarides (*E. sylvestre* auct.), requiring further taxonomic studies. *Malcolmia graeca* (Supplementary Data Table S1), representing the genus sister to *Erysimum* (Hendriks *et al.*, 2023), was selected as the outgroup for the phylogenetic analyses. Voucher specimens from all sampled populations are deposited in the herbarium SAV.

### Flow cytometric measurements

Flow cytometry was used to estimate relative nuclear DNA content following Doležel *et al.* (2007*a*, *b*) with a CyFlow Space flow cytometer (Partec, Münster, Germany) equipped with an ultraviolet light-emitting diode excitation source. A total of 296 individuals were measured for this study, supplemented by measurements of 200 individuals from previous work (Bačák *et al.*, 2026; for details, see above and Supplementary Data Table S1). Multiple individuals per population were analysed: three to ten for the target species and mostly two to four for the additional species (Supplementary Data Table S1). Silica gel-dried leaves were processed together with leaf tissue of internal standards in every flow cytometric run. As standards, we used either *Solanum lycopersicum* ‘*Stupické polní rané*’ (2C = 1.96 pg; Doležel *et al.*, 1992) or *Solanum pseudocapsicum* (2C = 2.59 pg; Temsch *et al.*, 2010), selected to avoid overlap in genome size with the sample. Each tube measured by flow cytometry contained both the sample and the internal standard tissue, and no samples were pooled. A protocol using Otto buffers (Doležel *et al.*, 2007*b*) and DAPI (4′,6-diamidino-2-phenylindole) fluorochrome was followed, as described by Bačák *et al.* (2026). The fluorescence intensity of 5000 stained nuclei was measured, and flow cytometric histograms were analysed using FloMax software v.2.7d (Partec, Münster, Germany). Relative nuclear DNA content (RGS; 2C value) was calculated from the ratio of the G0/G1 fluorescence peak positions of the sample and the internal standard (sample/standard ratio), expressed in arbitrary units (a.u.), with *S. lycopersicum* used as the primary standard. Only well-resolved histograms with coefficients of variation of <5 % were retained for analysis. RGS values were visualized in scatter plots to identify genome size categories (cytotypes) and summarized as boxplots by species and by cytotypes within species. To link these RGS values unambiguously to chromosome numbers, we counted chromosomes for selected individuals of each cytotype within each species (Fig. 2; Supplementary Data Table S1).

**Fig. 2.**
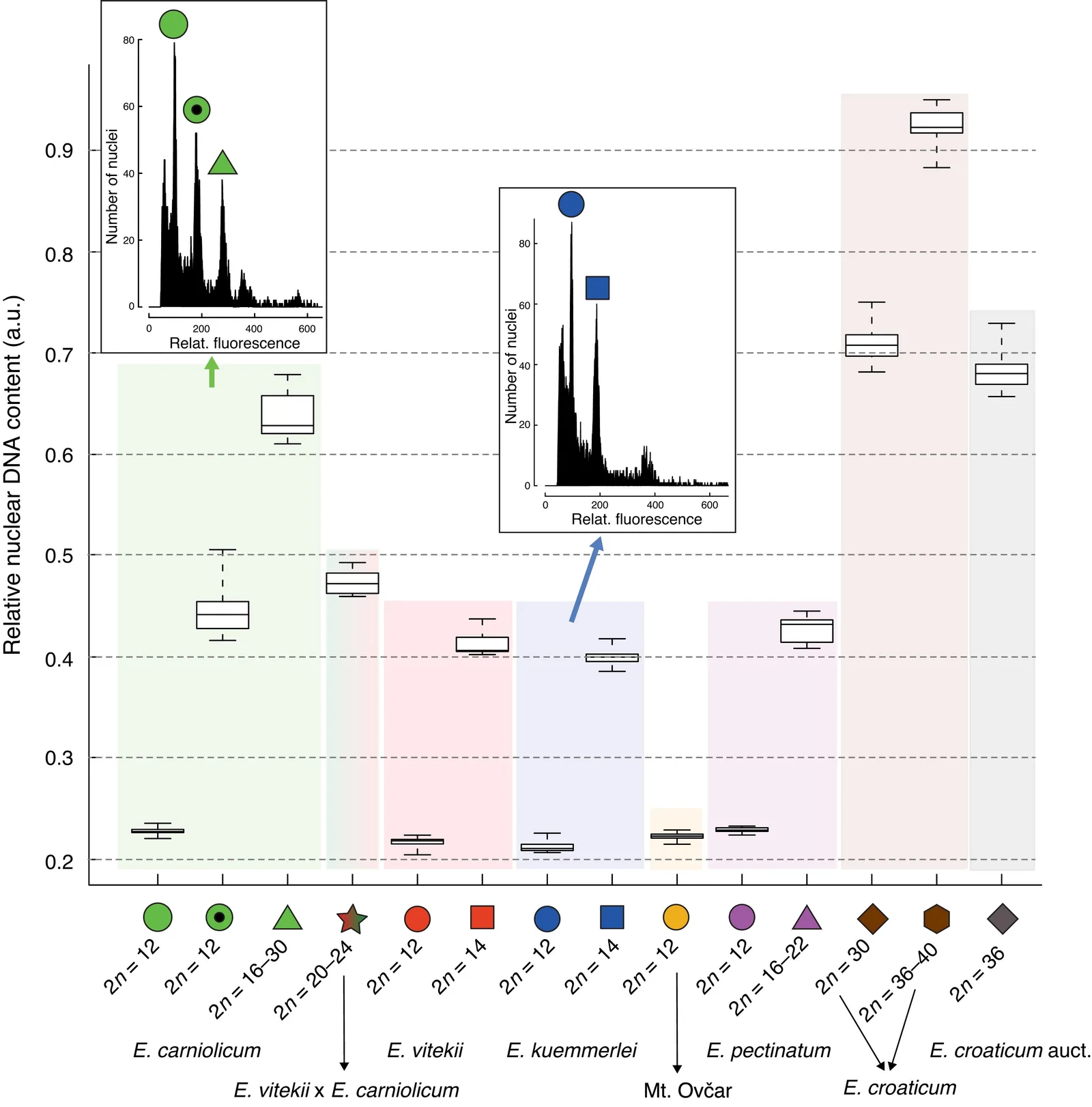
Variation in nuclear DNA content [sample/standard ratio, with *Solanum lycopersicum* used as the primary standard, given in arbitrary units (a.u.)] of the target *Erysimum* species as assessed by flow cytometry. The boxes represent the interquartile range, the horizontal lines within the boxes are the median values, and the whiskers extend to the minimum and maximum values. Symbols indicate different cytotypes (colours refer to species or entities, and shapes refer to cytotypes within the species differing in chromosome numbers and/or nuclear DNA content), as also depicted in Fig. 1. Insets show simultaneous flow cytometric analyses of three and two accessions of different cytotypes within *E. carniolicum* (accessions DURM, TUSBR and ZLAT) and *E. kuemmerlei* (accessions SHTR and Tomorrit; see Supplementary Data Table S1), respectively.

### Determination of the number of chromosomes

Chromosome counts were performed on mitotic spreads. Depending on material availability, mitotic spreads were prepared either from anther tapetum (young anthers collected and fixed either in the field or from plants grown from seeds) or from root tips (harvested from seedlings or cultivated plants) in a total of 98 accessions from 31 populations (Supplementary Data Table S1; Fig. S1). For each accession, 20 mitotic figures were examined, and one representative image was selected for presentation (Fig. 1; Supplementary Data Fig. S1). All chromosome preparations followed the protocol of Mandáková and Lysak (2016), after which slides were stained with DAPI (2 mg mL^−1^) in Vectashield antifade. Preparations were examined using a Zeiss Axioimager Z2 epifluorescence microscope equipped with a CoolCube camera (MetaSystems).

### Next-generation sequencing library preparation

Two next-generation sequencing methods were used: double-digest RAD sequencing (ddRADseq; Andrews *et al.*, 2016) and Hyb-Seq, which combines target enrichment with genome skimming (Weitemier *et al.*, 2014). Applying both methods in parallel has proved effective for elucidating recent, highly reticulate evolutionary histories involving polyploids (Karbstein *et al.*, 2022; Bačák *et al.*, 2026). RADseq provides numerous short loci and large numbers of genome-wide single-nucleotide polymorphisms (SNPs), whereas Hyb-Seq yields alignments of longer loci that allow allele phasing, homeologue identification and subgenomic tree reconstruction. For the target species, the same populations (Fig. 1A) were analysed with both methods, although RADseq included more individuals per population (two to five individuals) than Hyb-Seq (one or two individuals). For the additional, non-target species, only a subset of species and populations was included in Hyb-Seq, guided by preliminary RADseq results and excluding some species outside the main study area (for details, see Supplementary Data Table S1). Overall, the RADseq dataset comprised 234 individuals, and the Hyb-Seq dataset included 88 individuals. Both datasets integrated newly generated sequences with data previously published by Bačák *et al.* (2026), the latter including 92 RADseq and 30 Hyb-Seq samples.

Total genomic DNA was extracted from silica gel-dried leaves using the Exgene Plant SV kit (GeneAll, Seoul, Korea) and purified with 0.4× AMPure XP beads (Beckman Coulter, CA, USA). The ddRADseq libraries were prepared according to the published protocol (Peterson *et al*., 2012), with some modifications as described in detail by Šlenker *et al.* (2024). The Hyb-Seq libraries were prepared using the NEBNext Ultra II FS DNA Library Prep Kit and NEBNext Multiplex Oligos Kit for Illumina (New England Biolabs, MA, USA) following the manufacturer’s protocols, with some modifications as described by Bačák *et al.* (2026). For the target enrichment step, we used a custom set of Hyb-Seq probes developed for *Erysimum*, targeting 1004 exons from 955 genes, ranging in length from 695 to 1197 bp. The probe design followed the protocols outlined by Weitemier *et al.* (2014) and Schmickl *et al.* (2016), with details provided by Bačák *et al.* (2026). Both Hyb-Seq and RADseq libraries were sequenced with 150 bp paired-end reads on Illumina systems (MiSeq at BIOCEV, Czechia for Hyb-Seq, and NovaSeq at Novogene, UK for RADseq).

### RADseq data processing

The RADseq reads were demultiplexed using FASTX-Toolkit v.0.0.14 (http://hannonlab.cshl.edu/fastx_toolkit/index.html), quality-filtered using fastp v.0.20.1 (Chen, 2023) and deduplicated with clumpify.sh (BBTools; https://archive.jgi.doe.gov/data-and-tools/software-tools/bbtools/). The processed reads were mapped to the genome of *Erysimum cheiranthoides* (GenBank: GCA_011420285.1) using BWA v.0.7.5a (Li, 2013). Variant calling and filtering were performed using GATK v.4.4.0.0 (McKenna *et al.*, 2010), applying hard-filtering thresholds following GATK Best Practices (Van der Auwera *et al.*, 2013). Only biallelic SNPs with a minimum sequencing depth of 8× and ≤60 % missing genotypes were retained.

Phylogenetic relationships were reconstructed using a maximum likelihood (ML) approach implemented in RAxML-NG v.0.9.0 (Kozlov *et al.*, 2019), based on inputs in PHYLIP format with heterozygous sites encoded using IUPAC ambiguities. The GTR substitution model with Felsenstein’s ascertainment bias correction was applied. Conversion of the VCF file to PHYLIP format was carried out with the vcf2phylip.py script (Ortiz, 2019), and invariant sites were removed using the ascbias.py script (https://github.com/btmartin721/raxml_ascbias). Branch support was evaluated using 500 bootstrap replicates and the quartet sampling method (Pease *et al.*, 2018). Quartet sampling differentiates low support attributable to insufficient phylogenetic signal from that caused by phylogenetic discordance and further quantifies asymmetry in discordance that might indicate introgression. Additional insight into the overall genetic structure was obtained using NeighbourNet (NN) constructed in SplitsTree4 (Huson and Bryant, 2006) based on Nei’s genetic distances (Nei, 1972) calculated in the R package (Pembleton *et al.*, 2013) using R v.4.4.0 (R Core Team, 2024). Distances were estimated from a pseudo-diploidized VCF file retaining both homozygous and heterozygous genotypes.

Potential introgression events between the studied species were examined using the ABBA–BABA and related statistics (Durand *et al.*, 2011) as implemented in Dsuite (Malinsky *et al.*, 2021). Because the topology of the ML tree, particularly its backbone, showed low support (see Results), these tests were restricted to the clade comprising the target species. To generate the required input tree, we first identified RADseq loci spaced ≥1000 bp apart using the identifiRadLoci.workflow (Šlenker, 2024). For each RADseq locus and sample, FASTA sequences were generated with the FastaAlternateReferenceMaker tool in GATK, encoding heterozygous SNPs with IUPAC ambiguity codes. We selected RADseq loci ≥80 bp in length and containing a minimum of six phylogenetically informative sites (7168 loci in total) to infer gene trees in RAxML-NG (as described above). These gene trees were subsequently summarized into a species tree using ASTRAL-III (Zhang *et al.*, 2018). Two populations from Montenegro inferred to be of allotetraploid origin (see Results) were excluded.

To investigate possible origins of the polyploid populations from the target species (*E. croaticum* s.l. and the two tetraploid populations identified in Montenegro), the relatedness coefficients between individuals from these polyploids and all other sampled species were calculated, using the method-of-moments estimator implemented in PolyRelatedness (Huang *et al.*, 2014). Relatedness patterns were visualized using violin plots (R Core Team, 2020; Adler and Kelly, 2021).

### Hyb-Seq data processing

The Hyb-Seq reads were processed with HybPiper v.2.2.0 (Johnson *et al.*, 2016) to extract consensus sequences of the targeted exons and flanking regions (supercontigs). Potentially paralogous sequences were identified with HybPhaser (https://github.com/LarsNauheimer/HybPhaser) and excluded when the proportion of SNPs exceeded 5 %. This filtering resulted in a final dataset of 914 supercontigs. Consensus sequences were aligned using MAFFT v.7.450 (Katoh and Standley, 2013). Flanking regions and sites with gaps in >25 % of sequences were trimmed using the R package ips (Heibl, 2008) in R v.4.4.0 (R Core Team, 2024). For species-tree inference, gene trees were initially reconstructed in RAxML-NG using the best-fitting substitution models selected by the ModelFinder function in IQ-TREE v.1.6.12 (Chernomor *et al.*, 2016; Kalyaanamoorthy *et al.*, 2017) under the Bayesian information criterion. Branch support was assessed with 500 bootstrap replicates, and internal branches with bootstrap support ≤20 % were collapsed using Newick-Utilities v.1.6 (Junier and Zdobnov, 2010). The species tree was then inferred under the multispecies coalescent model implemented in ASTRAL-III (Zhang *et al.*, 2018), with branch support evaluated using local posterior probabilities (Sayyari and Mirarab, 2016). A NeighbourNet network was constructed following the same procedure as for the RADseq data, except that Nei’s genetic distances were calculated from VCF files generated by remapping Hyb-Seq reads to consensus exon sequences.

In addition, to gain insights into the polyploid genomes of *E. croaticum* s.l. and two tetraploid populations from Montenegro, their supercontig sequences were subjected to read-backed phasing to infer allele sequences, following the procedure described by Šlenker *et al.* (2021). Two complementary approaches were then used to identify distinct homeologues and determine their phylogenetic placement, thereby revealing the most likely parental species or lineages: EPA-ng (Barbera *et al.*, 2019) and AlleleSorting (Šlenker *et al.*, 2021). EPA-ng is a re-implementation of the evolutionary placement algorithm (EPA) that places allelic sequences onto a reference phylogenetic tree using maximum likelihood. To avoid bias from unequal sampling, a single centroid sequence per species was selected using usearch (Edgar, 2010). Only placements with a likelihood weight ratio >0.9 were considered reliable and retained for further processing. Major homeologue types, defined as those representing ≥15 % of the polyploid genome, were subsequently used for subgenomic species tree inference in ASTRAL-III.

In the AlleleSorting approach (applicable only to tetraploid samples), alleles were sorted into two homeologues based on sequence divergence, then attributed to putative parental lineages [setting the threshold of between_homeolog_distance to 1 and between_parents_distance to 0.5; see Šlenker *et al.* (2021) and https://github.com/MarekSlenker/AlleleSorting for more details]. The labelled homeologues, representing the two subgenomes of each tetraploid, were treated as independent accessions in the ASTRAL-III species tree inference.

### Morphometric analyses

Morphometric measurements were conducted on field-collected individuals at the flowering stage that were preserved as herbarium specimens. From each plant, one flower was detached. Its parts were spread out and fixed to a sheet of paper with transparent tape to maintain their original size and shape. In total, 31 vegetative and floral characters were measured, and 24 ratio characters were derived. One original character used in the ratio calculation was excluded from the final data matrix. To identify pairs of highly correlated traits (using a threshold of *r* > 0.95) that could bias discriminant analyses, non-parametric Spearman rank correlations were calculated for all characters. When such correlations were detected, one character from each pair was removed from the final matrices. A detailed list of all characters is provided in Table 1. Sheets with floral parts were scanned and measured using ImageJ v.1.53e software (Schneider *et al.*, 2012). Trichomes were examined and measured with a stereomicroscope (Olympus SZ61) and QuickPHOTO Micro v.3.2 software. The final data matrix for multivariate analyses comprised 42 characters and 495 specimens representing *E. carniolicum*, *E. kuemmerlei*, *E. pectinatum*, *E. vitekii* and *E. croaticum* s.l. (Supplementary Data Table S1). Subsets of this matrix were used for specific partial analyses (see Results). Cluster analyses (based on population means) and canonical discriminant analyses (based on individual plants) were performed using the MorphoTools2 package (Šlenker *et al*., 2022) in R v.4.0.0 (R Core Team, 2020). In addition to the characters included in the multivariate analyses, we recorded the presence of different trichome types (trichomes with two to six rays) on petals, stems, leaves and siliques from the same set of herbarium specimens.

**Table 1.** List of morphological characters measured or scored in the present study.

***Floral characters***	
*LP1*	Length of petal (including claw and blade)
*LP2^a^*	Length of petal blade
*LP3*	Length from the widest point of the petal blade to its tip
*WPmax*	Max. width of petal blade
*LS*	Length of sepal
*LSty*	Length of style
*WSti*	Width of stigma
*LF*	Length of longer filaments
***Ratios of floral characters:*** *LP2/LP1, LP2/WPmax, LP3/LP2*	
***Stem and leaf characters***	
*LL1*	Length of lower stem leaf (including petiole if present)
*LL2*	Length of middle stem leaf
*LL3^a^*	Length of upper stem leaf
*WLmax1^a^*	Max. width of lower stem leaf
*WLmax2^a^*	Max. width of middle stem leaf
*WLmax3^a^*	Max. width of upper stem leaf
*LLw1^a^*	Length from the widest part of the lower stem leaf to its tip
*LLw2^a^*	Length from the widest part of the middle stem leaf to its tip
*LLw3^a^*	Length from the widest part of the upper stem leaf to its tip
*NL^b^*	Number of stem leaves in the upper half of the leafy part of stem
*NT1^a^*	Number of teeth on one side of the lower stem leaf margin
*NT2^a^*	Number of teeth on one side of the middle stem leaf margin
*NT3^a^*	Number of teeth on one side of the upper stem leaf margin
*DT1*	Max. depth of teeth on lower stem leaf margin
*DT2*	Max. depth of teeth on middle stem leaf margin
*DT3*	Max. depth of teeth on upper stem leaf margin
*NTF1^c^*	Number of primary teeth bearing secondary teeth on lower stem leaf margin
*NTF2^c^*	Number of primary teeth bearing secondary teeth on middle stem leaf margin
*NTF3^c^*	Number of primary teeth bearing secondary teeth on upper stem leaf margin
*HStem^a^*	Height of the leafy part of the stem
***Ratios of stem and leaf characters:*** *LL1/LL2, LL2/LL3, NL/HStem, LL1/HStem, LL2/HStem, LL3/HStem, LL1/WLmax1, LL2/WLmax2, LL3/WLmax3, LLw1/LL1, LLw2/LL2, LLw3/LL3, NT1/LL1, NT2/LL2, NT3/LL3, DT1/WLmax1, DT2/WLmax2, DT3/WLmax3, NTF1/NT1^c^, NTF2/NT2^c^, NTF3/NT3^c^*	
***Trichome characters***	
*NrTrL*	Number of trichomes on the upper surface of the middle stem leaf per area 0.5 × 0.5 mm^2^
*LTrRay^d^*	Max. length of the trichome ray on the upper surface of the middle stem leaf (μm), measured for ≥3-fid trichomes
*LTrRay2^d^*	Max. length of the trichome ray on the upper surface of the middle stem leaf (μm), measured for 2-fid trichomes

^a^Characters excluded from the matrix owing to a high correlation with another character (ρ > 0.95) and/or used only for computing ratios.

^b^Lower stem leaves can be shed at the flowering stage; therefore, the total number of stem leaves could not be determined reliably, and only those in the upper stem half were counted.

^c^Secondary teeth are absent on the leaves of *E. croaticum*, *E. croaticum* auct., *E. odoratum* and *E. witmannii*; therefore, these characters were omitted from the analyses including these species.

^d^Measured within a single randomly selected visual field of the stereomicroscope. Because the rays of 2-fid trichomes are substantially longer than those of trichomes with three or more rays (≥3-fid trichomes), these two trichome types were treated separately. In *E. carniolicum*, *E. vitekii*, *E. kuemmerlei*, *E. pectinatum*, *E. odoratum* and *E. witmannii*, which possess 2- to 5(6)-fid trichomes but predominantly ≥3-fid forms, only the latter were measured (character LTrRay). In contrast, for *E. croaticum* s.l., only the rays of 2-fid trichomes were measured (character LTrRay2), because these are dominant and 3-fid trichomes occur only rarely.

Finally, we examined the morphological differentiation of the target western Balkan species from *E. odoratum* and *E. witmannii*, given the reported ambiguity in distinguishing *E. odoratum* from *E. carniolicum* (see Introduction). For this purpose, the data matrix generated here was supplemented with eight population samples of *E. witmannii* and eight population samples of *E. odoratum* from the previous study (Bačák *et al.*, 2026), selected to represent different intraspecific lineages.

## RESULTS

### Nuclear DNA content and chromosome numbers

Flow cytometric estimates of nuclear DNA content, together with chromosome counts, revealed substantial variation across all focal species, *E. carniolicum*, *E. kuemmerlei*, *E. pectinatum*, *E. vitekii* and *E. croaticum* s.l., with each species comprising at least two distinct cytotypes (Figs 1 and 2; Supplementary Data Fig. S1).

In *E. carniolicum*, relative nuclear DNA content (relative genome size, RGS) values clustered into three well-separated size groups (0.23 ± 0.004, 0.44 ± 0.019 and 0.64 ± 0.023 RGS units; Fig. 2). The two smaller-genome cytotypes, despite clearly distinct RGS values, shared the same number of chromosomes (2*n* = 12; Fig. 1; Supplementary Data Table S1; Fig. S1). The largest-genome cytotype displayed chromosomal variation, with 2*n* = 16 in the Jasik and MGLC populations and with 2*n* = 18 and 2*n* = 30 in the ZLAT population (Supplementary Data Table S1; Fig. S1). All three cytotypes occurred in the southern part of the species range (Serbia, Montenegro, Bosnia and Herzegovina), whereas only one (2*n* = 12; 0.44 RGS units) was observed in northern populations in Slovenia and Croatia (Fig. 1).

Both *E. vitekii* and *E. kuemmerlei* comprised two cytotypes differing in genome size (0.21–0.22 vs. 0.40–0.41 RGS units) and number of chromosomes (2*n* = 12 vs. 2*n* = 14; Figs 1 and 2; Supplementary Data Fig. S1). No geographical structure was apparent in *E. vitekii*, whereas *E. kuemmerlei* showed a clear north–south pattern (Fig. 1).

In *E. pectinatum*, two cytotypes were also detected (0.23 ± 0.003 vs. 0.43 ± 0.013 RGS units). The smaller-genome cytotype had 2*n* = 12 chromosomes, and the larger-genome cytotype exhibited either 2*n* = 16 (population PTER) or 2*n* = 22 (population MSVN; Supplementary Data Fig. S1). Their spatial distribution indicates a north–south pattern with overlap in the central Peloponnese (Fig. 1).

The taxonomically unassigned population from Mt. Ovčar (Ovčarsko-Kablarska klisura gorge, western Serbia) showed a single cytotype (0.22 ± 0.004 RGS units; 2*n* = 12). In contrast, two Montenegrin populations (codes OBGL and VRMAC), shown to be of hybrid origin (see below), displayed a cytotype with higher genome size (0.47 ± 0.011 RGS units) and variable numbers of chromosomes (2*n* = 20, 22 and 24; Fig. 1; Supplementary Data Table S1; Fig. S1).

Finally, *E. croaticum* from Croatia and Bosnia and Herzegovina comprised two cytotypes differing in genome size (0.71 ± 0.017 vs. 0.92 ± 0.022 RGS units) and number of chromosomes (2*n* = 30 vs. 2*n* = 36, 38 and 40). Populations from Kosovo (*E. croaticum* auct.) had a single cytotype (0.68 ± 0.019 RGS units; 2*n* = 36; Fig. 1; Supplementary Data Table S1; Fig. S1).

### Sequencing data characteristics

Sequencing of the RADseq libraries yielded an average of 5 509 109 (1 024 913–22 985 571; Supplementary Data Table S2) quality-filtered and deduplicated read pairs per sample. Mapping rates to the reference genome ranged from 63.69 to 97.43 %, with a mean of 90.00 %. The final VCF file contained 763 107 SNPs distributed across 18 076 putatively unlinked genomic regions (RADseq loci).

Sequencing of the Hyb-Seq libraries yielded an average of 708 902 (341 667–1 250 979) quality-filtered and deduplicated read pairs per individual. Of these, an average of 56.12 % (43.56–61.78 %; Supplementary Data Table S2) mapped to the 1004 targeted nuclear loci. Targeted loci were assembled using the HybPiper2 pipeline (Johnson *et al.*, 2016). To minimize missing data, we retained only loci recovered in >96 % of sampled individuals, corresponding to 94 % of the total targeted loci. Sequence recovery across these loci was high, reaching 99.9 %. After excluding outlier sequences with excessive variability, 914 sequences were retained, with lengths ranging from 774 to 1414 bp and a mean length of 1076.45 bp.

### Phylogenetic relationships and intraspecific genetic patterns

The three western Balkan endemics examined here, *E. carniolicum*, *E. kuemmerlei* and *E. vitekii*, formed a well-supported phylogenetic clade in both RADseq- and Hyb-Seq-based inferences [Fig. 3A, B; Supplementary Data Fig. S2A; bootstrap support (BS) = 92 %; quartet concordance (QC) = 0.4; and local posterior probability (LPP) = 0.88]. The taxonomically unassigned population from Mt. Ovčar in Serbia was consistently recovered as a separate lineage placed within this clade, strongly supported as sister to *E. kuemmerlei* (BS = 100 %, QC = 0.63 and LPP = 0.98). The placement of *E. vitekii* within this western Balkan clade, however, remained ambiguous. Depending on the dataset, it was inferred as sister to either *E. carniolicum* (RADseq; Fig. 3A; Supplementary Data Fig. S2A) or *E. kuemmerlei* (including the Ovčar branch; Hyb-Seq; Fig. 3B). However, both alternative topologies received weak support (BS = 27 %, QC = 0.29 and LPP = 0.6), and the low quartet differential score (QD = 0.11; Supplementary Data Fig. S2A) indicates an imbalance in the frequencies of the two discordant topologies. Additionally, the Alpine–Dinaric *E. sylvestre* auct. was nested within this western Balkan clade (Fig. 3A, B), appearing closest to *E. carniolicum*. Yet, its position was poorly supported in the RADseq tree (BS = 43 % and QC = −0.09) and was associated with a QD score of zero (Supplementary Data Fig. S2A).

**Fig. 3.**
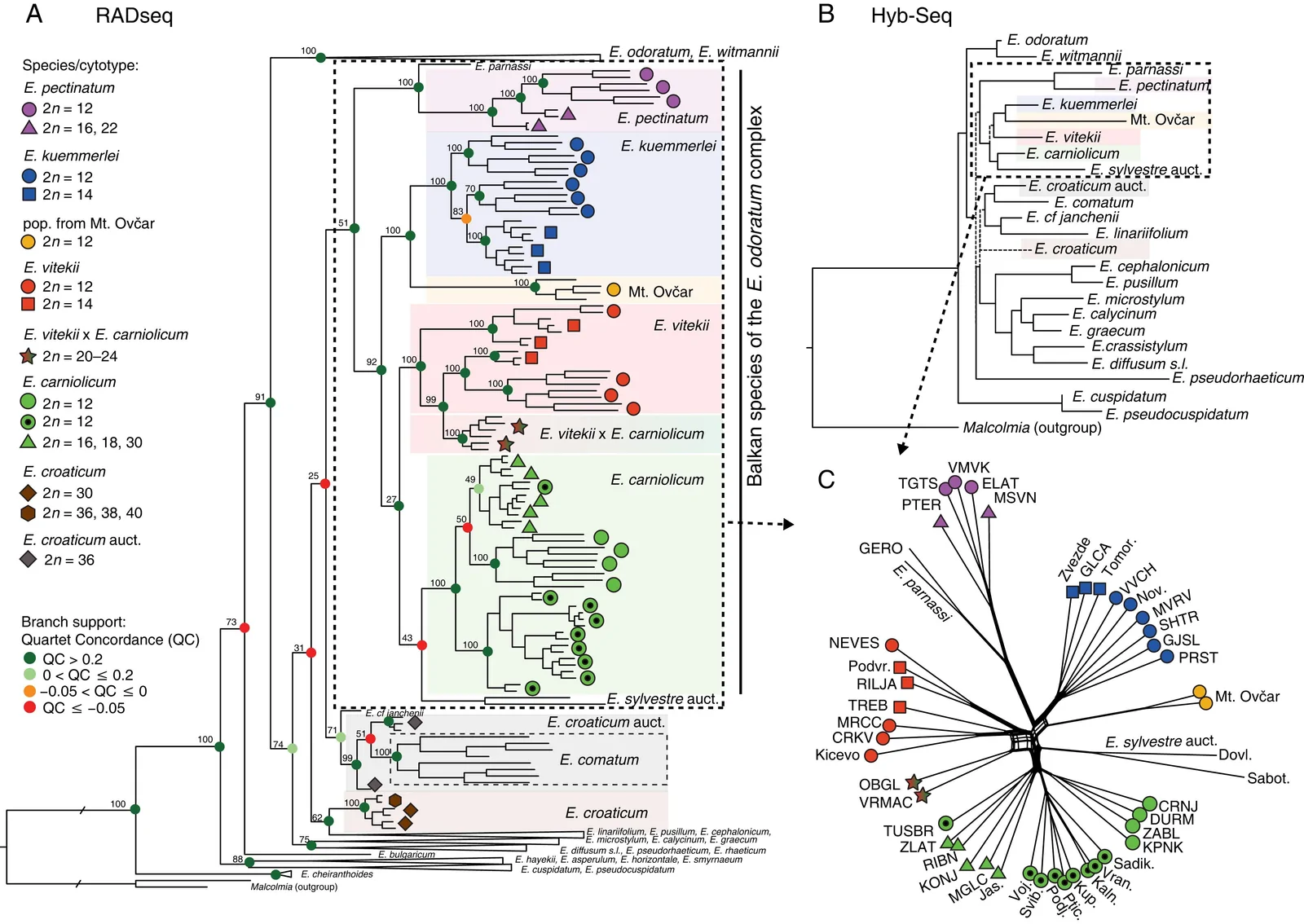
Phylogenetic relationships and genetic structure of the target *Erysimum* species (highlighted with colours and/or symbols, following Figs 1 and 2) and relatives. (A) Maximum likelihood tree inferred in RAxML based on RADseq data. Clades of non-target species are shown collapsed. See Supplementary Data Fig. S2 for the complete tree including all terminal labels. Branch support (quartet concordance symbols in the nodes and bootstrap values above the branches) is shown, except for the terminal intraspecific clades. (B) Species tree inferred in ASTRAL-III from 914 gene trees based on Hyb-Seq sequences, omitting the allotetraploid accessions of *E. vitekii* × *E. carniolicum* (OBGL, VRMAC, shown in the subgenomic tree in Fig. 4B). Branches with low support (LPP < 0.8) are depicted with dotted lines. (C) NeighbourNet analysis of the target clade (outlined in A and B), including the allotetraploids (OBGL and VRMAC), based on Hyb-Seq data. Population codes follow Supplementary Data Table S1.

The Peloponnese endemic *E. pectinatum* formed a clade clearly distinct from *E. kuemmerlei* (Fig. 3). It was resolved as sister to *E. parnassi*, an endemic of Mt. Parnassos, and together these two lineages were connected to the western Balkan clade described above. However, this relationship was supported only weakly in both RADseq (Fig. 3A; Supplementary Data Fig. S2; BS = 51 %, QC = 0.29 and QD = 0.11) and Hyb-Seq trees (Fig. 3B; LPP = 0.42). Finally, *E. croaticum* s.l. was divided into two distinct lineages, neither of which showed a close affinity to the other western Balkan endemics. Populations from Croatia (including the type locality) and Bosnia and Herzegovina, representing *E. croaticum* s.s., formed a well-supported clade whose phylogenetic placement remained uncertain (Hyb-Seq; Fig. 3B) or appeared sister to a clade comprising several other Balkan species (RADseq; Fig. 3A). In contrast, populations from Kosovo (*E. croaticum* auct.) formed a different, strongly supported clade together with the Carpathian–Balkan species *E. comatum* (BS = 99 %, QC = 0.26 and LPP = 0.97; Fig. 3).

All four western Balkan endemics, *E. carniolicum*, *E. kuemmerlei*, *E. vitekii* and *E. pectinatum*, formed well-supported, species-specific clades, despite pronounced intraspecific variation in genome size and number of chromosomes (see above). Within each species, both RADseq and Hyb-Seq analyses reveal marked intraspecific genetic structure (Fig. 3A, C; Supplementary Data Fig. S2B), which corresponded primarily to geographical origin and only secondarily to cytotype patterns. In *E. carniolicum*, three clades/clusters aligned with distinct geographical regions as follows: (1) northern populations from Slovenia and Croatia (0.44 RGS units, 2*n* = 12); (2) populations from Montenegro together with a geographically distant population from Mt. Kopaonik in Serbia but bearing the same cytotype (all 0.23 RGS units and 2*n* = 12); and (3) populations from the border region of Serbia, Montenegro, and Bosnia and Herzegovina. This last group included populations with the largest genomes (0.64 RGS units, 2*n* = 16–30) but also the geographically close population (code TUSBR, western Serbia), despite possessing the cytotype typical for northern populations (0.44 RGS units, 2*n* = 12). In *E. vitekii*, three clades/clusters likewise corresponded to the three sampled geographical regions but only partly to cytotype variation. Two geographically proximate populations in Bosnia and Herzegovina (population codes RILJA and NEVES) clustered together despite differing in cytotypes (0.21 vs. 0.40 RGS units). Finally, in *E. kuemmerlei*, the three clades/clusters resolved matched three geographical regions, with the two northern groups sharing the same cytotype (0.22 RGS units, 2*n* = 12).

### Assessing polyploid origins and the presence of interspecific gene flow

Two populations from Montenegro (codes OBGL and VRMAC; Supplementary Data Table S1) possessed a unique cytotype (0.47 ± 0.011 RGS units, 2*n* = 20–24) that clearly distinguished them from the nearby populations of *E. vitekii* and *E. carniolicum* (all with 0.21–0.23 RGS units, 2*n* = 12). In the ML tree inferred from RADseq data, these populations were placed within the clade of *E. vitekii* (Fig. 3A), yet the NeighbourNet analyses of both RADseq (Supplementary Data Fig. S2B) and Hyb-Seq (Fig. 3B) data consistently indicated an intermediate position between the clusters of *E. vitekii* and *E. carniolicum*. This pattern, combined with their ∼2-fold DNA content and number of chromosomes relative to adjacent populations, suggests a possible allotetraploid origin, which we tested explicitly. The relatedness estimator (PolyRelatedness) based on RADseq data showed the highest pairwise values between these Montenegrin populations and both *E. carniolicum* and *E. vitekii* (Fig. 4A). Hyb-Seq-based analyses using the EPA-ng and AlleleSorting approaches each identified two distinct homeologs in their genomes, phylogenetically placed as sister to *E. carniolicum* and *E. vitekii*, respectively, confirming their allotetraploid origins (Fig. 4B). More specifically, EPA-ng unambiguously classified 32–34 % of alleles, of which 29–33 % were assigned to the ‘vitekii’ type and 20–24 % to the ‘carniolicum’ type. The remaining alleles were distributed at low frequencies (<10 %) across a broad set of species (Supplementary Data Table S3). In the AlleleSorting analysis, 26–28 % of exon sequences passed the filtering thresholds (see the Materials and Methods), and their alleles could be sorted unambiguously into homeologue A (labelling of the alleles with shorter distance to the branch of *E. vitekii*, one of the suspected progenitors based on the topology of the RADseq tree; Fig. 3A) and homeologue B. In the ASTRAL species tree, homeologue A was resolved as sister to *E. vitekii*, whereas homeologue B was placed as sister to *E. carniolicum* (Fig. 4B).

**Fig. 4.**
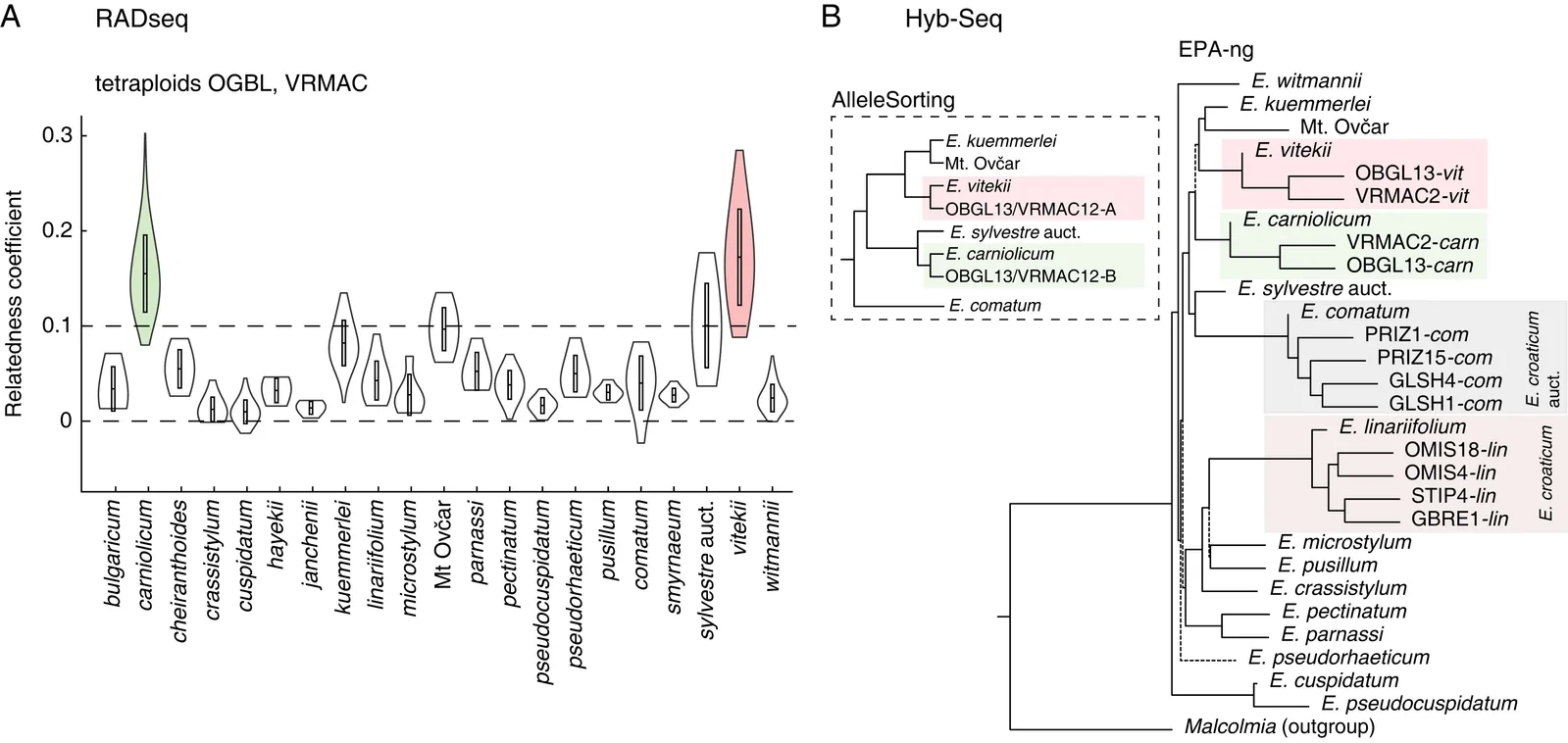
Origin of polyploids assessed from RADseq (A) and Hyb-Seq (B) data. (A) Violin plots showing the distribution of pairwise relatedness values between the tetraploids from Montenegro (populations VRMAC and OBGL; see Fig. 1A; Supplementary Data Table S1) and the sampled species (only those with relatedness mean values >0.0 are shown) computed from RADseq data using the PolyRelatedness tool. The boxes within the violin plots represent the s.d., and the horizontal lines within the boxes are the mean values. Violin plots for the polyploids of *E. croaticum* s.l. are shown in Supplementary Data Fig. S3. (B) Species tree inferred in ASTRAL-III from subgenomic gene trees constructed from Hyb-Seq alignments of diploid species and the homeologues identified in the polyploid accessions (tetraploid accessions OBGL and VRMAC, accessions of *E. croaticum* and *E. croaticum* auct.), labelled and assigned to subgenomes using the phylogenetic placement algorithm implemented in EPA-ng. The homeologues are labelled as *vit*, *carn*, *lin* and *com* according to their phylogenetic positions. Only the major homeologue types with a frequency of >15 % were processed (see Supplementary Data Table S3). Branches with low support (LPP < 0.8) are depicted with dotted lines. The inset in frame shows the clade of the ASTRAL-III species tree inferred following homeologue sorting and labelling using the AlleleSorting tool (applicable to tetraploids only).

We further investigated the polyploid (presumably hexaploid) origin of *E. croaticum* s.l. using both the relatedness estimator in PolyRelatedness based on RADseq data and the EPA-ng approach applied to Hyb-Seq sequences. The relatedness estimator yielded equivocal results (Supplementary Data Fig. S3): elevated relatedness values were detected with several Balkan species. For accessions of *E. croaticum* from Croatia and Bosnia and Herzegovina, these included the polyploids *E. cephalonicum*, *E. diffusum* s.l., *E. graecum* and *E. janchenii*, in addition to the diploid *E. linariifolium*. For accessions from Kosovo, elevated relatedness was observed with the polyploids *E. cephalonicum*, *E. diffusum* s.l. and *E. janchenii* and with the diploid *E. comatum*. EPA-ng analysis of Hyb-Seq data provided a clearer signal. For *E. croaticum* accessions from Croatia and Bosnia and Herzegovina, 23–25 % of alleles were classified, of which 18–22 % were assigned to the ‘linariifolium’ type, and the remaining alleles were distributed among other species at lower frequencies (<11 %; Supplementary Data Table S3). For *E. croaticum* auct. from Kosovo, 27–30 % of alleles were classified, of which 28–35 % were assigned to the ‘comatum’ type, with the rest assigned to other species at lower frequencies (<15 %; Supplementary Data Table S3). The subgenomic species tree (Fig. 4B) indicated that *E. croaticum* s.l. does not share a single polyploid origin. Instead, the data support independent autopolyploid origins from different progenitors in the Adriatic (Croatia, Bosnia and Herzegovina) vs. inland (Kosovo) lineages.

Next, we used ABBA–BABA tests to investigate signals of interspecific gene flow within the clade comprising the target species *E. carniolicum*, *E. kuemmerlei*, *E. pectinatum* and *E. vitekii*, including the unassigned population from Mt. Ovčar but excluding the two allotetraploid populations from Montenegro (see above). The f-branch statistics (Supplementary Data Fig. S4) revealed strong introgression signals between *E. sylvestre* auct. and *E. carniolicum*, particularly involving the southern populations of the latter, which is consistent with the quartet differential (QD) score of zero in the ML tree (see above). Introgression was also identified between the unassigned population from Mt. Ovčar and *E. carniolicum*, especially involving the populations of the same cytotype, characterized by the lowest genome size and a chromosome number of 2*n* = 12, distributed in Montenegro and in the Kopaonik Mts. in Serbia. Furthermore, strong signals of interspecific gene flow were detected between *E. vitekii* and *E. kuemmerlei* and between *E. vitekii* and *E. carniolicum* (notably their Montenegrin populations). These results are congruent with the uncertain phylogenetic placement of *E. vitekii*, its low QD score in the ML tree (see above) and the existence of allotetraploid populations involving the latter two species (see above).

### Multivariate morphometrics

Cluster analysis based on population means of 30 morphometrically analysed populations revealed three major clusters, corresponding to: (1) *E. pectinatum* together with *E. kuemmerlei*; (2) *E. croaticum* s.l.; and (3) *E. carniolicum* and *E. vitekii*, along with the two populations of allotetraploid origin (see above) and the taxonomically unassigned population from Mt. Ovčar (Fig. 5A). CDA of 495 individual specimens, with seven *a priori* defined groups (excluding allotetraploids), identified *E. croaticum* s.l. as the most morphologically distinct (Fig. 5B). Canonical structure coefficients indicate that this distinctiveness is mainly attributable to long, narrow leaves with sparse and shallow marginal teeth (Supplementary Data Fig. S5B; see also Fig. 6G). A subsequent CDA excluding *E. croaticum* s.l. resolved three clearly distinct clusters of *E. carniolicum*, *E. vitekii* and *E. kuemmerlei* intermingled with *E. pectinatum* (Fig. 5C). Specimens of *E. kuemmerlei* and *E. pectinatum* are characterized by conspicuously pinnatisect leaves (densely and deeply dentate leaf margins, with secondary teeth) and comparatively small floral parts (Fig. 6D, E; Supplementary Data Fig. S5C). *Erysimum carniolicum* and *E. vitekii* were separated from each other along the second canonical axis, mainly reflecting differences in floral size (larger in *E. carniolicum*), density of teeth on leaf margins (higher in *E. carniolicum*) and traits such as number of leaves, the length of leaves and trichome rays on leaf surface (all higher in *E. vitekii*; Supplementary Data Fig. S5C; see also Fig. 6A, B). Specimens from the two allotetraploid populations, passively projected into this CDA, occupied intermediate positions between *E. carniolicum* and *E. vitekii*. Finally, the specimens from Mt. Ovčar overlapped mostly with *E. vitekii*, consistent with the placement of this population in the clustering analysis (Fig. 5A). A separate CDA (Fig. 5D) including only *E. vitekii*, *E. kuemmerlei* and specimens from Mt. Ovčar, supported the distinct morphology of this taxonomically unassigned population. Relative to *E. vitekii*, plants in this population have narrower petals and wider basal leaves.

**Fig. 5.**
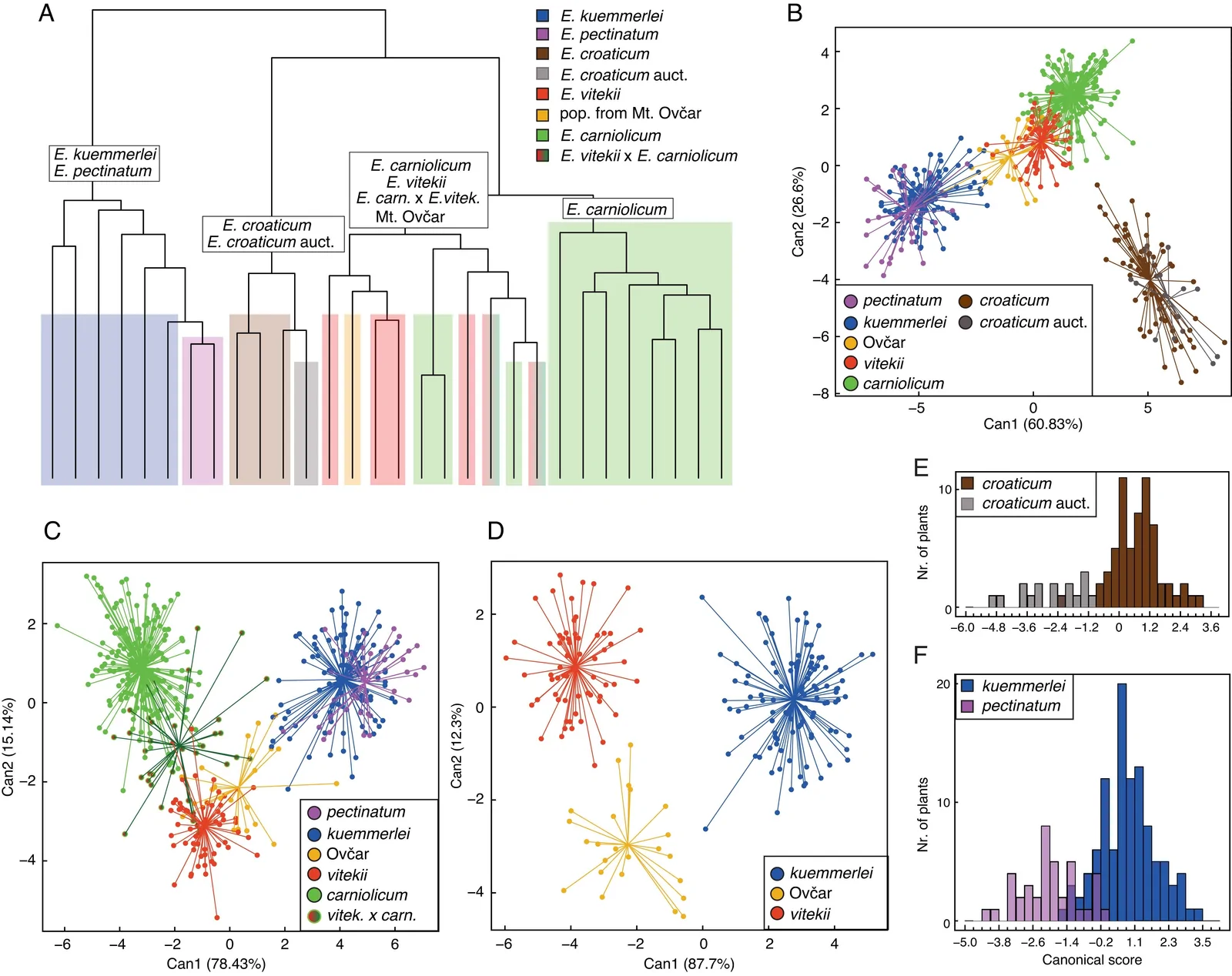
Morphometric analyses of Balkan species of the traditionally defined *Erysimum odoratum* complex. (A) UPGMA cluster analysis based on the matrix of 30 sampled populations (listed in Supplementary Data Table S1) and population means of 41 characters (excluding the LTrRay and LTrRay2 characters; see Table 1). (B) Canonical discriminant analysis (CDA) based on 495 specimens and 41 characters (as in A), with seven *a priori* defined entities as indicated (excluding the tetraploid populations of *E. vitekii* × *E. carniolicum*). (C) CDA based on 451 specimens and 42 characters (including the LTrRay character), with six entities as indicated. Unlike the previous CDA in B, the most distinct *E. croaticum* and *E. croaticum* auct. were omitted here, and specimens of *E. vitekii* × *E. carniolicum* were included as passively projected objects. (D) CDA based on 192 specimens and 42 characters (as in C), with three defined entities as indicated. The taxonomically unassigned population from Mt. Ovčar is differentiated here from the morphologically closest species. In the graphs in B–D, the specimen points are connected to the centroid of the given group, producing a spider-like diagram. (E) CDA based on 79 specimens and 42 characters (including the LTrRay2 character), with two entities, *E. croaticum* (Croatia, Bosnia and Herzegovina) and *E. croaticum* auct. (Kosovo). (F) CDA based on 146 specimens and 42 characters (including the LTrRay character), with two entities, *E. kuemmerlei* and *E. pectinatum*. Total canonical structure coefficients, expressing the contributions of characters to the axes, are shown in Supplementary Data Fig. S5.

**Fig. 6.**
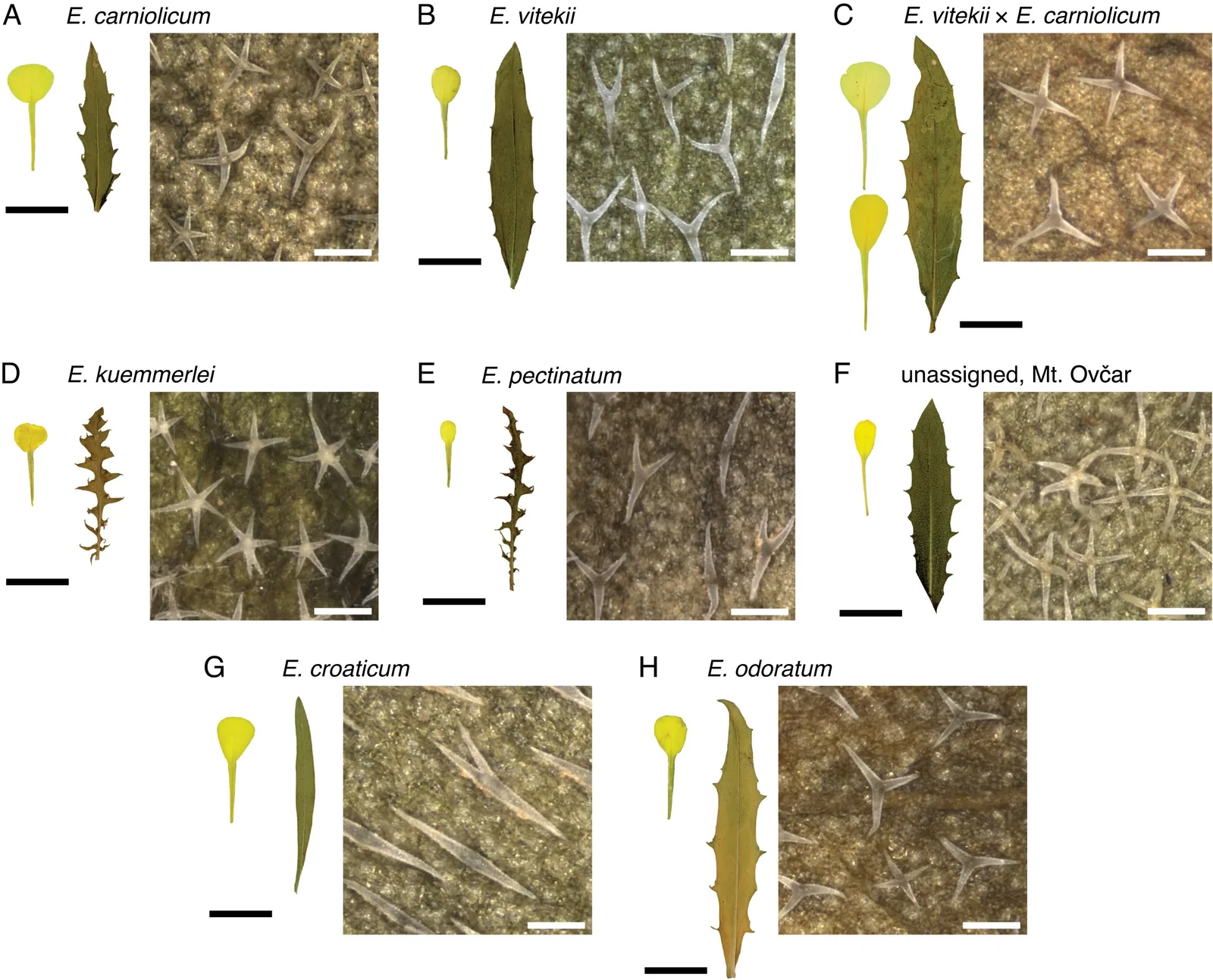
Scans and photographs of selected morphological features of the Balkan species and entities recognized here within the traditionally defined *Erysimum odoratum* complex, including petals, middle stem leaves, and trichomes on the upper surface of middle stem leaves. Scale bars: 1 cm for petals and leaves, 0.25 cm for trichomes. (A) *Erysimum carniolicum*, population ZLAT. (B) *Erysimum vitekii*, population Podvranić. (C) *Erysimum vitekii* × *E. carniolicum*, population OBGL, two petals are shown to illustrate large variation in their shape. (D) *Erysimum kuemmerlei*, population GJSL. (E) *Erysimum pectinatum*, population PTER. (F) Taxonomically unassigned entity from Mt. Ovčar, population OVCA. (G) *Erysimum croaticum*, population Omis. (H) *Erysimum odoratum*, populations Zukici (petals, middle stem leaf), V.Ucka (trichomes). Population codes follow Supplementary Data Table S1.

Two additional CDAs examined differentiation within two species pairs that appeared intermingled in the above analyses: *E. croaticum* (Croatia, Bosnia and Herzegovina) vs. *E. croaticum* auct. (Kosovo) and *E. kuemmerlei* vs. *E. pectinatum*. CDA of *E. croaticum* s.l. (Fig. 5E; Supplementary Data Fig. S5E) showed that the population from Kosovo differs by narrower petals, longer upper stem leaves and shorter trichome rays on leaves. In the comparison of *E. kuemmerlei* and *E. pectinatum* (Fig. 5F; Supplementary Data Fig. S5F), CDA showed that *E. kuemmerlei* can be distinguished by having more stem leaves, higher trichome density on leaf surface and larger floral parts (see also Fig. 6D, E).

Variation in trichome types on stems, leaves, siliques and petals also contributed to species differentiation (Fig. 6; Supplementary Data Table S4). *Erysimum croaticum* s.l. is distinct in possessing exclusively 2- and 3-fid trichomes (Fig. 6G) on leaves and siliques, and predominantly 2-fid trichomes on petals and stems (with 3-fid trichomes occurring only rarely). In contrast, *E. carniolicum*, *E. vitekii* and *E. kuemmerlei* bear 2- to 5(6)-fid trichomes (Fig. 6A, B, D) on leaves, siliques and petals, and 2- to 4-fid trichomes on stems. *Erysimum pectinatum* differs by having only 2- and 3-fid trichomes on siliques, stems and petals (thus resembling *E. croaticum* s.l.), while having 2- to 4-fid trichomes on leaves (Fig. 6E; Supplementary Data Table S4).

Finally, we also assessed morphological differentiation of *E. carniolicum* and *E. vitekii* relative to two other species of the traditional *E. odoratum* complex, *E. odoratum* and *E. witmannii* (Supplementary Data Fig. S6). *Erysimum witmannii* (Carpathian diploids) differs from both Balkan species particularly by its longer, narrower stem leaves; from *E. carniolicum* additionally by less dentate leaves; and from *E. vitekii* by wider petals and longer sepals. *Erysimum odoratum* (widespread polyploids) is clearly separable from *E. carniolicum* by its smaller floral parts, less dentate leaves, more stem leaves and longer trichome rays on the leaf surface. Although *E. odoratum* and *E. vitekii* showed some morphological overlap, *E. odoratum* typically has more leaves, less dentate leaf margins, smaller upper stem leaves relative to middle stem leaves (LL2/LL3 ratio), longer sepals and a wider stigma (Supplementary Data Fig. S6).

An identification key to the members of the traditional *E. odoratum* complex in the Balkans is presented. In addition, Fig. 6 illustrates some of the diagnostic characters that aid species identification, namely variation in trichome types, variably dentate margins of middle stem leaves, and differences in petal size and shape. Descriptive statistics for all quantitative characters measured (Table 1) per species are presented in Supplementary Data Table S5.

## DISCUSSION

### Taxonomic perspectives: morphological and genetic distinction of the studied Balkan endemics

Our results show that the three Balkan species traditionally assigned to the *Erysimum odoratum* complex, *E. carniolicum*, *E. kuemmerlei* and *E. pectinatum*, together with the more recently described *E. vitekii* (Polatschek, 2013), represent distinct, separately evolving lineages, supporting their recognition as species. In our analyses, all four lineages were resolved as monophyletic and morphologically distinguishable (Figs 3, 5 and 6; see also Identification key). Published occurrence data (Strid, 1989; Jalas and Suominen, 1994; Polatschek and Snogerup, 2002; Polatschek, 2013) and our present sampling (Fig. 1) further indicate largely allopatric ranges, with only narrow zones of contact or sympatry. Such geographical separation generates extrinsic reproductive barriers, which, however, may break down in sympatry. This was observed between *E. carniolicum* and *E. vitekii*, but such events appear rare, spatially restricted, and in this case led to a new allopolyploid entity, rather than eroding species integrity through extensive introgression. No clear differentiation among species was observed in chromosome numbers and genome size variation, because several cytotypes (2*n* = 12, 14 and 16) were shared across species. In contrast, we detected substantial variation within species, highlighting pronounced genome size and chromosomal dynamics in the genus, and particularly in this species complex (see below). Overall, following the unified species concept (de Queiroz, 2007; Karbstein *et al.*, 2024), these four lineages exhibit several properties (lines of evidence) to consider them as separately evolving metapopulation lineages and to treat them as distinct species. Finally, *E croaticum* s.l. appears to comprise two independently arisen, genetically divergent lineages despite their morphological resemblance. Additional sampling and analyses are required to resolve them taxonomically (see also below).

Despite earlier ambiguities and misidentifications (Jalas and Suominen, 1994; Vassilev *et al.*, 2012, 2016, 2018), we have demonstrated that *E. carniolicum*, the most widespread of these Balkan endemics, is well differentiated from the broadly distributed and parapatric *E. odoratum*. Several diagnostic characters reported for *E. carniolicum* (Polatschek, 2013), such as large petals, absence of sterile axillary shoots, persistence of lower stem leaves during flowering and mostly glabrous petals (see Supplementary Data Table S4), are supported by our observations. Additional differences in the number of stem leaves, leaf margin and trichome-ray length (Supplementary Data Table S5; Fig. S6) further distinguish these two species. We assume that past confusion between *E. carniolicum* and *E. odoratum* might have resulted from two main factors: their parapatric distribution and the distinct species *E. vitekii*, described only relatively recently from Montenegro (Polatschek, 2013). Range overlap between *E. carniolicum* and *E. odoratum* occurs mainly in north-western Croatia (Polatschek, 2013; Nikolić, 2015), where they may co-occur even within the same locality (e.g. in the Učka Mts., Istria; see Supplementary Data Table S1). In Bosnia and Herzegovina and in Serbia, their distributions seem to be separated in a north–south direction (Jalas and Suominen, 1994; Polatschek, 2013; Stupar *et al.*, 2018; Bačák *et al.*, 2026; and present records), with some zone of sympatry but with elevational differentiation: *E. carniolicum* typically grows above 1000 m a.s.l., whereas *E. odoratum* inhabits lower elevations. Our southernmost record of *E. odoratum* in Serbia is from the Kavlar peak (790 m a.s.l.; Supplementary Data Table S1), previously misidentified as *E. carniolicum* (Lakušić, 2019).

In Montenegro, confusion in classification has probably been caused by populations later described as *E. vitekii* (Polatschek, 2013), which are shown here to be morphologically closest to *E. odoratum* (Supplementary Data Fig. S6). The previously proposed absence of trichomes on petals in *E. vitekii* (Polatschek, 2013) was found to be an unreliable trait (see Supplementary Data Table S4). Both *E. odoratum* and *E. vitekii* typically bear 2- and 3-fid trichomes on petals, but differ in other characters, including the number of stem leaves, leaf margin and size of some floral parts (Supplementary Data Table S5; Fig. S6; see also Identification key). Despite their overall morphological similarity, these two species are phylogenetically divergent and allopatric. *Erysimum vitekii* appears confined to Montenegro and the southernmost parts of Bosnia and Herzegovina, reported also from southern Croatia (Pelješac) and northern Albania (Polatschek, 2013; and present records).

The geographically disjunct *E. kuemmerlei* and *E. pectinatum* are morphologically similar, as previously noted, and sometimes even merged into a single species (Josifović *et al.*, 1972; Vangjeli, 2017). However, we identified differences in the number of stem leaves, trichome density on leaf surface, trichome type frequency and size of floral parts (Supplementary Data Table S4; Fig. S5) and, importantly, proved their phylogenetic divergence, finally supporting their recognition as two separate species. Their resemblance, particularly in pinnatifid to pinnatisect stem leaves (Fig. 6D, E), might reflect parallel evolution, perhaps by selection on ancestral standing genetic variation (Thompson *et al.*, 2019; James *et al.*, 2021).

The morphological differentiation presented and reported here was based on field-collected plants, which might partly reflect environmentally induced plastic variation. However, our recent parallel datasets of cultivated and field-collected samples in *E. odoratum* and *E. witmannii* (Bačák *et al.*, 2026) indicated that traits of the floral organs (shape and size), trichomes, and leaf shape and margin are generally stable, whereas the number of leaves and the size of vegetative structures (stems and leaves) can be affected by environmental conditions. Growing plants in a common garden would therefore be valuable in future work for the Balkan endemics, as it would help to disentangle environmentally driven variation from genetically based morphological differences.

### Phylogenetic resolution, impact of interspecific gene flow, and major evolutionary drivers

Our phylogenetic reconstruction provides initial insights into the evolutionary relationships among Balkan *Erysimum* species, although it covers only part of the species diversity in the peninsula (Supplementary Data Fig. S2). Several internal nodes have low support, as indicated by low bootstrap and quartet concordance values, along with both high and low quartet differential (QD) values. This suggests that incomplete lineage sorting and reticulate evolution both contribute to these patterns, consistent with previous phylogenetic studies in the genus (Züst *et al*., 2020). For the five focal species, the western Balkan endemics *E. carniolicum*, *E. kuemmerlei*, *E. vitekii* and *E. croaticum* s.l. and the Peloponnese endemic *E. pectinatum*, our results largely confirm the phylogenetic patterns inferred in an earlier study based on limited sampling (one to three populations per species; Bačák *et al.*, 2026). A notable exception concerns *E. vitekii*. Its position was previously well resolved as sister to *E. kuemmerlei* based on the single known cytotype (2*n* = 12), but with the discovery of an additional cytotype (2*n* = 14) and inclusion of both cytotypes in the present analyses, its placement becomes ambiguous (Fig. 3). Low QD values, together with ABBA–BABA tests (Supplementary Data Fig. S4), point towards introgression rather than incomplete lineage sorting as a likely explanation for conflicting branching patterns, although weak phylogenetic support limits the confidence with which these processes can be distinguished. Novel and well-supported phylogenetic findings of the present study include the identification of a taxonomically unrecognized population from Mt. Ovčar and two distinct lineages within *E. croaticum* s.l. (see below).

Although gene flow was inferred between several of the species studied here, their mostly allopatric distributions currently limit opportunities for hybridization and might reinforce their genetic divergence. Geographical distance and biogeographical barriers have repeatedly been shown to shape both intra- and interspecific differentiation in this genus (Moazzeni *et al.*, 2014; Züst *et al*., 2020; Bačák *et al.*, 2026). The topographically complex landscapes of the Balkans facilitate geographical structuring, a pattern also documented in other plant groups (Španiel and Rešetnik, 2022). *Erysimum carniolicum* and *E. kuemmerlei* are clearly allopatric, with their ranges separated by the well-known biogeographical barrier formed by the Drin river basin and Kosovo Polje, which separates the Dinaric mountains from the Scardo-Pindhic range (Griffiths *et al.*, 2004; Stevanović *et al.*, 2009). This barrier marks distribution limits for many Balkan species (Španiel and Rešetnik, 2022) and also separates subspecies (e.g. *Cerastium decalvans*, Niketić *et al.*, 2022) or intraspecific lineages (*Salvia officinalis*, Rešetnik *et al.*, 2016*a*; *Knautia drymeia*, Rešetnik *et al.*, 2016*b*; *Euphorbia epithymoides* s.l., Đurović *et al.*, 2024). Ecological adaptation, whether climatic, elevational or edaphic, may also contribute to speciation in the target complex, but probably in concert with geographical isolation. The range of *E. vitekii* appears restricted to the southern Dinaric mountains adjacent to the Adriatic coast, which resembles the distribution of some other, mostly thermophilous western Balkan endemics (*Euphorbia hercegovina*, Sharovikj Ivanova *et al.*, 2025) or intraspecific lineages (*Edraianthus tenuifolius*, Surina *et al.*, 2011; *Viola suavis*, Mereďa *et al.*, 2011; *Salvia officinalis*, Rešetnik *et al.*, 2016a). Populations of *E. vitekii* rarely ascend above 1000 m, which separates them from the high-elevation species *E. carniolicum*. Furthermore, *E. vitekii* grows on calcareous bedrock, whereas both *E. kuemmerlei* and *E. carniolicum* have been recorded from calcareous, siliceous and serpentine substrates (Polatschek, 2013).

Polyploidy has also been a significant evolutionary driver in the study complex, including autopolyploidization inferred for *E. croaticum* s.l. and allopolyploidy (*E. carniolicum* × *E. vitekii*) confirmed in populations from Montenegro (Fig. 4). This latter case illustrates how allopolyploids can arise in secondary contact zones of related species or genetic lineages, a scenario also documented in other plant groups in the Balkans (see e.g. Zozomová-Lihová *et al.*, 2020; López-González *et al.*, 2021; Lazarević *et al.*, 2022; Šlenker *et al.*, 2024). A comparable parapatric distribution along the elevational gradient resulting in a hybrid zone was observed in *Erysimum* in the Sierra Nevada in south-eastern Spain; however, in that case hybridization occurred without an increase in ploidy level and was characterized by extensive backcrossing (Abdelaziz *et al.*, 2021). In contrast, the tetraploid level of the hybrids in our study appears to act as a reproductive barrier to the diploid parental species, preventing the formation of hybrid swarms. The allopolyploid plants occupy a relatively large area, spreading a few kilometres along the roadside, and produce viable seeds (J. Zozomová-Lihová, personal observation), which indicate successful establishment and the potential for long-term persistence and allopolyploid speciation (Soltis and Soltis, 2009; Edger *et al.*, 2025).

### The diversity hotspot and cryptic diversification in the southern Dinarides

The results presented here reveal a pronounced diversity hotspot in the southern Dinarides, spanning south-western Serbia, Kosovo, southern Bosnia and Herzegovina, and Montenegro (Fig. 1A). This region forms a major contact zone where several of the studied species meet and multiple cytotypes co-occur, including previously unrecognized cryptic entities. We recorded a heterogeneous, polyphyletic *E. croaticum* s.l., allotetraploids derived from *E. vitekii* and *E. carniolicum*, and a taxonomically unassigned population from Mt. Ovčar. At the cytotype level, all three cytotypes of *E. carniolicum* and both cytotypes of *E. vitekii* occur here. These findings are consistent with other cases of remarkable and often cryptic diversity in the region, documented at the genetic, cytotypic or species levels (Kučera *et al.*, 2010; Kutnjak *et al.*, 2014; Španiel *et al.*, 2017; Grdiša *et al.*, 2019; Đurović *et al.*, 2021; Kuzmanović *et al.*, 2021; Sharovikj Ivanova *et al.*, 2025). All these patterns suggest that the southern Dinarides provided favourable conditions for long-term diversity accumulation. The environmentally complex landscapes forming a mosaic of diverse habitats support high species richness, while strong topographic barriers restrict migration and gene flow, fostering genetic divergence. Estimates of divergence times in the genus *Erysimum* (Moazzeni *et al.*, 2014; Huang *et al.*, 2020; Hendriks *et al.*, 2023) allow us to link speciation and diversification of the focal species to the Pleistocene epoch. Therefore, we also suggest that the moderate impact of Pleistocene glaciations in this area and the climatic buffering effect of the nearby Adriatic Sea contributed to environmental stability and population persistence in (micro)refugia (Griffiths *et al.*, 2004; Hughes *et al.*, 2006; Španiel and Rešetnik, 2022). This concentration of local diversity, in turn, increased opportunities for secondary contacts, hybridization and polyploidization events (e.g. Lakušić *et al.*, 2009; Kučera *et al.*, 2010; Šlenker *et al.*, 2024).

One of the cryptic entities identified here is the population from Mt. Ovčar in the Ovčarsko-Kablarska klisura gorge in western Serbia, previously recorded and treated by Polatschek (2013) as *E. carniolicum*. Our results show that this population is morphologically closest to *E. vitekii* (Fig. 5), yet phylogenetically it is placed as sister to *E. kuemmerlei* (Fig. 3). Despite this discrepancy, a hybrid origin appears unlikely: the population is diploid, it exhibits high seed set and germination rates (J. Zozomová-Lihová, unpublished research), and our attempts to identify different subgenomes from Hyb-Seq data (not shown) disproved them. Although *E. kuemmerlei* has been reported from south-western Serbia (Lakušić, 2019), our sampling shows that these records represent misidentifications of *E. carniolicum*. In contrast, the northernmost confirmed localities of *E. kuemmerlei* are in Kosovo (Polatschek, 2013; and present results). We therefore suggest that the entity from Mt. Ovčar might have arisen from a common ancestor with *E. kuemmerlei* either through past range fragmentation (vicariance) or through long-distance dispersal, followed by isolation and divergence; hypotheses that require further testing. Its morphological and genetic divergence, in addition to geographical separation, suggest its treatment as a distinct species, but given the limited data available on its distribution, we refrain from proposing a formal taxonomic treatment at this stage. So far, only a single population of this entity has been confirmed, but it occupies a relatively large area on the southern slopes of Mt. Ovčar at elevations ∼700–900 m a.s.l. The possibility that it represents a stenoendemic taxon should also be considered, as illustrated by *Edraianthus canescens* D. Lakušić, a recently described stenoendemic restricted to the calcareous cliffs of the Ovčarsko-Kablarska klisura (Lakušić *et al.*, 2013).

Another previously unrecognized entity is represented by the tetraploids found along the roadside in the Sinjajevina Mts. in Montenegro. Both genomic and karyological data support their allotetraploid origin (Figs 2 and 4), consistent with their morphological intermediacy (Fig. 5C) and their location in the contact zone between populations of *E. carniolicum* and *E. vitekii*. These plants also document remarkable chromosomal dynamics and rapid reduction in the number of chromosomes, which appears characteristic of the entire species complex. Here, we found counts of 2*n* = 20 and 2*n* = 22 occurring alongside the regular tetraploid number 2*n* = 24, while maintaining a uniform genome size (Fig. 2; Supplementary Data Fig. S1).

Finally, our results support Polatschek’s (2013) treatment of *E. croaticum* as a distinct entity, in addition to morphological differentiation between populations from Kosovo and the Adriatic coast. Although the morphological differences are subtle (Fig. 5), the phylogenetic divergence between these disjunct populations is likely to reflect independent polyploidization events (Fig. 4B), supporting separate taxonomic treatment. Further sampling in western Kosovo, however, is needed, particularly to clarify the distinction from *E. janchenii* Fritsch, which was proposed as a distinct tetraploid species extending to western Kosovo (Polatschek, 2013) but otherwise synonymized with diploid *E. linariifolium* (Jalas and Suominen, 1994; Caković and Stešević, 2021).

### Intraspecific divergence: geographical structuring and remarkable cytotype variation

The pronounced genetic structuring observed within the studied Balkan endemics reflects both geographical regions and cytotype variation. This suggests that intraspecific divergence is shaped by a combination of extrinsic gene flow barriers, such as geographical distance, biogeographical barriers or historical colonization patterns, as documented for other plant species in the Balkans (reviewed by Španiel and Rešetnik, 2022), and intrinsic barriers associated with differences in chromosome number and genome size. Nevertheless, the geographical and cytotype patterns were not fully congruent. This is particularly evident in the southern Dinarides, where we identified cytotypically mixed genetic lineages in both *E. vitekii* and *E. carniolicum* (Figs 1 and 3). A key to understanding these patterns is likely to lie in the origin and evolution of these cytotypes; however, discrepancies between genome sizes and chromosome numbers point to complex patterns of chromosomal changes. Within species and among close relatives, genome size generally increases proportionally with chromosome number and ploidy level (Sliwinska *et al.*, 2022) and has therefore been widely used as a ploidy estimator (e.g. Suda *et al.*, 2007; Balao *et al.*, 2009; Kobrlová *et al.*, 2024). However, this relationship is not always straightforward. In polyploids, a reduction in monoploid genome size, known as genome downsizing, has frequently been documented and is associated with the gradual process of diploidization (Leitch and Bennett, 2004; Mandáková and Lysak, 2018). Moreover, the activity of repetitive DNA can modify genome size in both directions, further decoupling genome size from chromosome number or ploidy level (McCann *et al.*, 2020; Pellicer *et al.*, 2021). As a result, the relationship between chromosome number and genome size can become complex, because both traits can evolve independently across different phylogenetic lineages of a genus (Macas *et al.*, 2015; Campos-Domínguez *et al.*, 2022). Such complexity is also evident in our target species, where we observe discrepancies between chromosome number and genome size. What is particularly striking is that the degree of mismatch we detect would typically be expected among different phylogenetic clades, yet in our case it occurs among closely related species and even within them.

The discrepancy between chromosome number and genome size is absent in the lowest genome-size category (sample/standard ratio ≈ 0.2 RGS units; Fig. 2), where all examined species (*E. carniolicum*, *E. vitekii*, *E. kuemmerlei* and *E. pectinatum*) consistently exhibit 2*n* = 12. In contrast, discrepancies emerge at genome size values ≈ 0.4–0.5 RGS units, where we observe 2*n* = 12 (in *E. carniolicum*), 2*n* = 14 (*E*. *vitekii* and *E. kuemmerlei*) and 2*n* = 16–22 (*E*. *pectinatum*). These patterns might reflect autotetraploidy undergoing rapid structural diploidization manifested by a reduction in the number of chromosomes (Mandáková and Lysak, 2018). The most extreme case occurs in *E. carniolicum*, where some populations share the same number of chromosomes but differ 2-fold in genome size (Fig. 2). This highlights the need for future cytogenomic and genomic approaches to determine whether this mismatch between genome size and chromosome number results from recent autopolyploidy followed by accelerated reduction in the number of chromosomes, great variation in repetitive DNA content or a combination of both processes operating in parallel.

Nevertheless, whether arising from polyploidy, chromosomal rearrangements, repetitive DNA evolution or their combination, the co-existence of multiple cytotypes within the study species might limit gene flow between populations (Ramsey and Schemske, 1998; Kolář *et al.*, 2017). When such populations are also geographically separated, such as in *E. kuemmerlei* and *E. carniolicum*, both extrinsic and intrinsic barriers may operate together, potentially accelerating divergence and promoting speciation. Nevertheless, different ploidy levels or cytotypes do not always constitute complete reproductive barriers (Kolář *et al.*, 2017), as also shown in Iberian *Erysimum* (Osuna-Mascaró *et al.*, 2023). Furthermore, here we also detected unique cases of intrapopulational cytotype variation. Some populations (Supplementary Data Table S1) comprise individuals with different chromosome number yet exhibit uniform genome size. This represents an unusual pattern of intraspecific dysploidy, similar to that documented in *Cardamine pratensis* (Melichárková *et al.*, 2020) and recently reported in *E. witmannii* (Bačák *et al.*, 2026). Taken together, these findings reinforce the view that *Erysimum* is among the most cytogenetically dynamic and variable genera in the Brassicaceae (Moazzeni *et al.*, 2014).

### Conclusion

Our study of Balkan *Erysimum* endemics revealed predominantly allopatric patterns and congruence between genetic and morphological data, clarifying species boundaries while also uncovering interspecific gene flow and cryptic diversity. The southern Dinarides emerged as a prominent diversity hotspot, where we found multiple cytotypes, inferred both auto- and allopolyploidization events and identified a previously unrecognized diploid entity. Overall, these findings underscore how geographical isolation, polyploidy and dynamic chromosomal evolution interact to drive diversification. This combination of processes helps to explain the exceptional diversity and complex evolutionary histories characteristic of the Balkan flora.

## IDENTIFICATION KEY TO THE MEMBERS OF THE *ERYSIMUM ODORATUM* COMPLEX IN THE BALKANS

1a. Trichomes on leaves 2-fid (trichomes with two rays)^1^, occasionally also 3-fid (trichomes with three rays), but never ≥4-fid; stem leaves narrow: middle stem leaves (2–)3(–4) mm wide, 9–23-times longer than wide, and long relative to stem height (reaching 1/5 to 1/3 of stem height), with only sparse and shallow teeth on the leaf margin … *E. croaticum* s.l.^2^

1b. Trichomes on leaves of multiple types, including 2-, 3-, 4-, 5-fid^1^, and occasionally also 6-fid; stem leaves wider: middle stem leaves (4–)7(–12) mm wide, three to eight times longer than wide, and shorter relative to stem height (reaching 1/8 to 1/5 of stem height), with several conspicuous teeth on the leaf margin … 2

2a. Stem leaves with deeply dentate leaf margins^1^, with primary teeth reaching up to the midrib (pinnatisect leaves) and with secondary teeth commonly present¹; petals (9–)12(–15) mm long^3^ and (2–)4(–6) mm wide … 3

2b. Stem leaves with conspicuous teeth on the leaf margin^1^, reaching up to one-third of the leaf width, rarely with secondary teeth present; petals larger, (13–)19(–25) mm long^3^ and (3–)7(–10) mm wide … 4

3a. Trichomes on leaves 3-, 4- and 5-fid^1^, occasionally also 2-fid; trichomes on siliques 3-, 4- and 5-fid, rarely also 6-fid; number of stem leaves in the upper stem half^4^ (5–)10(–17); petals ≤16 mm long^3^ and 6 mm wide … *E. kuemmerlei* (Albania, Kosovo, North Macedonia)

3b. Trichomes on leaves 2-, 3- and 4-fid^1^; trichomes on siliques only 2- and 3-fid; number of stem leaves in the upper stem half^4^ (4–)7(–11); petals ≤13 mm long^3^ and 4 mm wide … *E. pectinatum* (Peloponnese)

4a. Petals (16–)21(–25) mm long^3^ and (6–)8(–10) mm wide; stems mostly simple, rarely branched … *E. carniolicum*

4b. Petals (13–)16(–19) mm long^3^ and (3–)5(–7) mm wide; stems mostly branched … 5

5a. Petal blades oblong or narrowly obovate^1^, (5–)6(–7) mm long and (3–)4(–5) mm wide, ratio of petal blade length/width (1.3–)1.6(–2.1); lower stem leaf margin with (4–)6(–9) teeth on one side, occasionally with secondary teeth present … taxonomically unassigned population from the Ovčarsko-Kablarska klisura gorge (Mt. Ovčar) in Serbia

5b. Petal blades obovate^1^, (5–)7(–8) mm long and (4–)6(–7) mm wide, ratio of petal blade length/width (0.9–)1.2(–1.5); lower stem leaf margin with (2–)5(–7) teeth on one side, secondary teeth absent … 6

6a. Sepals (7–)8(–9) mm long, style (8–)10(–12) mm long, stigma (0.6–)0.9(–1.2) mm wide; number of stem leaves in the upper stem half^4^ (6–)13(–23); lower stem leaf margin with teeth ≤4.5 mm long, those on the middle stem leaf margin ≤3 mm long; upper stem leaves (3–)6(–10) mm wide, middle stem leaves ∼1.5 times longer than upper stem leaves … *E. vitekii* (southern Bosnia and Herzegovina^5^, southern Croatia (Pelješac), Montenegro, northern Albania)

6b. Sepals (7–)9(–11) mm long, style (8–)11(–15) mm long, stigma (0.7–)1(–1.6) mm wide; number of stem leaves in the upper stem half^4^ (9–)20(–31); lower stem leaf margin with teeth ≤3.5 mm long, those on the middle stem leaf margin ≤2.5 mm long; upper stem leaves (2–)4(–8) mm wide, middle stem leaves ∼2 times longer than upper stem leaves … *E. odoratum* (widespread, extending to southern Bosnia and Herzegovina^5^ and central Serbia, but absent from Montenegro and Albania)

*Notes*. *Erysimum witmannii*, a diploid endemic of the Carpathians that does not extend to the Balkans, is not included in the key. The allotetraploids (*E. carniolicum* × *E. vitekii*), which combine the characters of the parental species and show a large range of variation in some characters (see petal shape variation in Fig. 6C), are also excluded. In contrast, the taxonomically unassigned population from the Ovčarsko-Kablarska klisura gorge is included in the key but requires further chorological and nomenclatural study to resolve its taxonomic treatment. For quantitative characters, means with 5th and 95th percentiles in parentheses are shown.

^1^Trichomes, leaves and petals of selected herbarium specimens are shown in Fig. 6.

^2^Including *E. croaticum* s.s. (Croatia, Bosnia and Herzegovina) and *E. croaticum* auct. from Kosovo (see Polatschek, 2013), because the latter requires further sampling and study to reach a taxonomic conclusion and is therefore not treated separately here.

^3^Length of petals including blade and claw.

^4^The number of stem leaves is given only for the upper half of the stem owing to unreliable counting in its lower part; lower stem leaves can be shed at the flowering stage (see Table 1).

^5^The distribution ranges of *E. vitekii* and *E. odoratum* could marginally overlap in southern Bosnia and Herzegovina (for distribution records of *E. odoratum*, see Bačák *et al*., 2026), but their precise range limits still need to be determined.

## Supplementary Material

mcag229_Supplementary_Data

## Data Availability

The Illumina sequencing reads generated for this study are available from the NCBI Sequence Read Archive under BioProject PRJNA1284580, with BioSample accession numbers specified in Supplementary Data Table S1.

## Supplementary Data

Supplementary data are available at *Annals of Botany* online and consist of the following. Figure S1: mitotic chromosomes of selected *Erysimum* accessions. Figure S2: maximum likelihood tree with quartet sampling and bootstrap support, and NeighbourNet based on RADseq data. Figure S3: violin plots showing the distribution of pairwise relatedness values between the polyploid *Erysimum croaticum* s.l. and other sampled species, computed from RADseq data using the PolyRelatedness tool. Figure S4: results of f-branch analysis computed from RADseq data in Dsuite. Figure S5: morphometric analyses as shown in Fig. 5, but also depicting the total canonical structure coefficients, expressing the contributions of characters to the canonical axes. Figure S6: canonical discriminant analyses depicting morphological differentiation between Balkan species *Erysimum carniolicum* and *E. vitekii*, and the core species of the *E. odoratum* complex, *E. odoratum* and *E. witmannii*. Table S1: list of the *Erysimum* populations analysed in present study, including relative nuclear DNA content values and chromosome numbers. Table S2: number of quality-filtered and deduplicated read pairs per sample, and percentage of reads mapped for Hyb-Seq and RADseq data. Table S3: assignment of alleles obtained from phased exon sequences (Hyb-Seq data) in the polyploids to diploid species using the EPA-ng algorithm. Table S4: variation in trichome types on petals, stems, leaves and siliques in the studied *Erysimum* species. Table S5: descriptive statistics for quantitative morphological characters measured in the studied *Erysimum* species.
