# Supplementary material for: Cryptic diversity, polyploidy and intraspecific divergence in Erysimum (Brassicaceae) species endemic to the western Balkans
Source: Ann Bot. 2026 Jul 30;138(5):1804–24. doi: 10.1093/aob/mcag229 (PMC13715005; doi:10.1093/aob/mcag229)

SUPPLEMENTARY MATERIALS

Table S1. List of the *Erysimum* populations analysed in present study, including relative nuclear DNA content values and chromosome numbers. **…see separate file TableS1_A3.pdf**

Table S2. Number of quality-filtered and deduplicated read pairs per sample, and percentage of reads mapped for Hyb-Seq and RADseq data.

Table S3. Assignment of alleles obtained from phased exon sequences (Hyb-Seq data) in the polyploids to diploid species using the EPA-ng algorithm.

Table S4. Variation in trichome types on petals, stems, leaves, and siliques in the studied *Erysimum* species.

Table S5. Descriptive statistics for quantitative morphological characters measured in the studied *Erysimum* species. **…see separate file TableS5_A3.pdf**

Figure S1. Mitotic chromosomes of selected *Erysimum* accessions.

Figure S2. Maximum-likelihood tree with quartet sampling and bootstrap support, and NeighbourNet based on RADseq data.

Figure S3. Violin plots showing the distribution of pairwise relatedness values between the polyploid *E. croaticum* s.l. and other sampled species, computed from RADseq data using the PolyRelatedness tool.

Figure S4. Results of f-branch analysis computed from RADseq data in Dsuite.

Figure S5. Morphometric analyses as shown in Figure 5, but depicting also the total canonical structure coefficients, expressing the characters' contributions to the canonical axes.

Figure S6. Canonical discriminant analyses depicting morphological differentiation between Balkan species *E. carniolicum* and *E. vitekii*, and the core species of the *E. odoratum* complex, *E. odoratum* and *E. witmannii*.

Table S2. Number of quality-filtered and deduplicated read pairs per sample, and percentage of reads mapped for Hyb-Seq and RADseq data. Population codes follow Table S1.

**Population code**	**Hyb-Seq**				**RADseq**		
	sample code	percentage of reads mapped to the targeted sequences	number of quality-filtered and deduplicated read pairs	number of retrieved sequences, quality filtered	sample code	percentage of reads mapped to the reference genome	number of quality-filtered and deduplicated read pairs
**Balkan species of the *Erysimum odoratum* species complex**							
E. carniolicum							
Kalnik	Kalnik4	55.57%	625610	914	Kalnik4	89.91%	1945630
					Kalnik5R	91.77%	5188275
Vranilac	Vranilac9	55.50%	618573	914	Vranilac9	87.85%	4687508
					Vranilac11R	88.54%	3122861
Kupec	Kupec8	56.49%	769966	914	Kupec8	91.19%	6100924
					Kupec10R	92.28%	5686812
Podjelak	Podjelak2	56.87%	775713	913	Podjelak2	90.57%	1522334
					Podjelak8	91.10%	1726907
Pticji	Pticji21	52.92%	683336	914	Pticji21	87.10%	3197492
					Pticji22	90.99%	2573162
Sadikovac	Sadikovac2	55.19%	694612	914	Sadikovac2	91.15%	3396642
					Sadikovac6R	92.79%	3214877
Vojak_c	Vojakc2	55.17%	595448	914	Vojak_c2	91.06%	3502511
					Vojakc10R	88.16%	4969508
MGLC	MGLC6	60.14%	576881	914	MGLC6	89.28%	5844403
					MGLC4	88.62%	3346978
Jasik	Jasik1	56.06%	627598	912	Jasik1	87.40%	4100284
					Jasik3	87.44%	9197486
DURM	DURM1	54.98%	722509	914	DURM1	91.12%	7896601
					DURM2	89.70%	6506941
CRNJ	CRNJ2	56.35%	575508	913	CRNJ2	91.15%	4669398
					CRNJ3	89.49%	2828639
Zabljak	Zabljak2	56.42%	1034953	914	Zabljak2	91.36%	2778145
					Zabljak1	92.40%	3203992
KPNK	KPNK23	59.72%	761389	914	KPNK23	90.15%	4626974
					KPNK26	90.21%	3264515
					KPNK31	91.08%	3515074
KONJ	KONJ19	60.90%	732202	913	KONJ19	90.41%	4550090
					KONJ14	91.16%	1222942
TUSBR	TUSBR2	56.04%	778152	913	TUSBR2	87.41%	3745997
					TUSBR1	91.74%	3326912
RIBN	RIBNs2	56.79%	761870	913	RIBNs2	90.25%	6344632
					RIBNs1	91.70%	2458663
ZLAT	ZLAT2	55.03%	341667	903	ZLAT2	90.57%	5501588
					ZLAT1	90.07%	8756633
Svibno	Svibno7	60.71%	730516	914	Svibno7	90.62%	2118798
					Svibno1	88.59%	2931299
E. croaticum							
Gbre	GBre1	61.22%	930158	914	CROA_GBRE_1	92.68%	3199885
					GBre2	92.37%	10099051
Omis	Omis18	56.79%	619392	913	Omis18	92.76%	1516531
	Omis4	53.97%	631658	914	Omis4	92.45%	3844727
Stipanici	Stipanici4	55.08%	549563	914	Stipanici4R	90.46%	8105826
					Stipanici1	89.88%	2057673
***E. croaticum* auct. (Kosovo)**							
GLSH	GLSH1R	55.98%	815555	914	GLSH1	90.81%	11188553
	GLSH4	56.38%	862706	914			
PRIZ	PRIZs1	57.69%	608639	914	PRIZs1	91.91%	3203679
	PRIZs15R	55.11%	814533	913	PRIZs15R	92.25%	5000263
					PRIZs12	90.61%	2947247

Population code	Hyb-Seq				RADseq		
	sample code	percentage of reads mapped to the targeted sequences	number of quality-filtered and deduplicated read pairs	number of retrieved sequences, quality filtered	sample code	percentage of reads mapped to the reference genome	number of quality-filtered and deduplicated read pairs
E. kuemmerlei							
SHTR	SHTRs3	60.22%	675788	913	SHTRs3	90.41%	6286738
					SHTR12	85.24%	2858154
					SHTR24_1	89.86%	7180415
Zvezde	ZvezdeP1	56.77%	783937	913	ZvezdeP1	91.94%	13917642
					ZvezdeP2	85.11%	4528430
Tomorrit	TomorritT7	59.71%	763610	913	TomorritT7	92.40%	2889571
					TomorritT1	91.53%	3406198
					TomorritT4	91.74%	8470099
					TomorritT6	92.39%	22985571
MVRV	MVRVs7	56.05%	607736	914	MVRVs7	84.89%	7978966
					MVRVs1	86.88%	2571867
GLCA	Galicica3	61.07%	812965	914	GalicicaEry1_1	87.53%	10137507
					GLCA2	90.72%	11015606
					GLCA6	80.71%	6030147
VVCH	VVCH1	60.10%	720916	914	VVCH1	86.64%	4061206
					VVCH2	87.68%	5881619
NovakE1	Novak1_11	59.04%	842163	914	NovakEry1_11	89.56%	6688151
					Novak_Ery1_12	86.51%	1129002
PRST	PRST2	54.75%	703915	914	PRST2	88.50%	5770235
					PRST1	88.10%	21361113
GJSL	GJSL1	55.14%	659414	914	GJSL1	92.39%	4075446
					GJSL2	91.57%	12032095
**population of ucertain taxonomic assignment, genetically close to *E. kuemmerlei***							
OVCA/Ovčar	OVCA24_2	55.54%	686395	913	OVCA24_2	83.33%	5594327
	OVCA24_3	55.33%	599996	914	OVCA24_3	87.20%	9912260
					OVCA2	85.20%	3204583
					OVCA3	89.38%	9180031
E. pectinatum							
TGTS	TGTS1	53.06%	1017103	913	TGTS1	90.80%	3218197
					TGTS2	91.38%	5957967
MSVN	MSVN1	61.66%	700575	914	MSVN1	92.07%	3326818
					MSVN2	91.73%	4293504
ELAT	ELAT1	53.23%	951351	914	ELAT1	89.69%	9095163
					ELAT2	86.73%	5376027
VMVK	VMVK2	57.23%	744826	914	VMVK2	86.93%	14177313
					VMVK1	91.26%	5454028
PTER	PTER1	54.53%	990948	914	PTER1R	90.22%	3884154
					PTER2R	89.07%	8729376
E. vitekii							
CRKV	CRKV3	60.99%	730369	914	CRKV3	85.44%	6443141
					CRKV1	90.42%	3190062
					CRKV24_2	91.79%	4360474
MRCC	MRCC2	53.69%	609513	913	MRCC2	83.59%	10270643
					MRCC7	86.24%	9619072
Kicevo	Kicevo1	54.39%	569062	914	Kicevo1	82.63%	18054660
					Kicevo2	84.01%	4456660
TREB	TREB1	59.91%	899857	914	TREB1	92.17%	2159814
					TREB2	92.55%	2174327
					TREB3R	92.53%	3261014
Podvranić	Podranic21	55.59%	659910	913	Podranic21	91.54%	2451715
					Podranic20	91.97%	2038795
					Podranic22R	92.33%	3796700
Neves	Neves1	55.30%	566141	914	Neves1	91.27%	8175729

Population code	Hyb-Seq				RADseq		
	sample code	percentage of reads mapped to the targeted sequences	number of quality-filtered and deduplicated read pairs	number of retrieved sequences, quality filtered	sample code	percentage of reads mapped to the reference genome	number of quality-filtered and deduplicated read pairs
					Neves2	91.60%	8999762
RILJA	RILJA2	60.26%	742087	912	RILJA2	90.62%	7403401
					RILJA1	89.89%	5097833

populations genetically admixed, intermediate between *E. vitekii* and *E. carniolicum* (suspected allopolyploids)

OBGL	OBGL13	52.31%	621760	914	OBGL13	84.30%	1645865
					OBGL12	89.13%	4466769
					OBGL5	87.56%	5960423
VRMAC	Vrmac2	50.88%	572721	913	Vrmac2	91.79%	19914647
					VRMAC1	92.11%	5562490
					VRMAC10	91.57%	3166708

***Erysimum odoratum* species complex in a stricter sense (following Bačák et al. 2026)**

E. odoratum

Bacin					Bacin2	91.81%	3031802
					Bacin3	92.38%	5277894
Maliscak	Maliscak9	59.99%	625460	912	Maliscak9	92.33%	6640672
					Maliscak1	91.62%	3494049
					Maliscak3	90.34%	1255991
CIUC	CIUC6R	58.32%	742341	914	CIUC6R	90.97%	2408633
					CIUC2	89.10%	4388443
					CIUC5	89.76%	3719929
RET	RET1s	52.87%	710288	914	RET1s	85.44%	4058210
					RET2s	86.99%	6340552
SCO	SCO3	55.34%	897656	914	SCO1s	90.01%	8429295
					SCO2s	88.99%	6201003
GLAV	GLAVs1	53.79%	674242	911	GLAVs1	92.20%	10294431
					GLAVs2	92.31%	6805768
Vgrad	VGrad1	54.70%	736116	913			
BREIT	BREITs1	60%	701956	914	BREITs1	93.08%	7696673
Zukici					Zukici13	91.14%	2552854
					Zukici14	91.71%	1746910
Busko					Busko22	92.31%	2310526
					Busko24	92.11%	1423164
Vojak_o					Vojak_o6	91.74%	4117551
V.Ucka					VUcka1	92.12%	7583707
M.Ucka					MUcka1	92.06%	4774243
Kavlar	Kavlar2	59.10%	615627	913	KAVLAR2	89.45%	5109507
					KAVLAR22	92.30%	6828443
Bacevci					Bacevci1	92.25%	3499644
					Bacevci2	92.26%	1024913

E. witmannii

GEMH	GEMHs1	59.33%	1016787	914	GEMHs1	91.59%	3372005
LES	LESc	60.27%	828422	914	LESc	91.57%	4450039
					witLESc3	88.95%	1711445
NASZ	NASZ8	56.57%	587128	914	NASZ10	92.57%	2395516
					NASZ4	92.56%	5369373
TUN	TUN2s	52.95%	908704	913	TUN2s	91.45%	3825210
					TUN1s	91.82%	7119901
ZARN	ZARN6s	61.78%	715125	913	ZARN6s	91.21%	4897224
					ZARN23_4s	89.54%	4610324
SUGA	SUGA1sm	59.88%	671175	914	SUGA1sm	85.24%	5503477
					SUGA23_4s	91.38%	9422264

Population code	Hyb-Seq				RADseq		
	sample code	percentage of reads mapped to the targeted sequences	number of quality-filtered and deduplicated read pairs	number of retrieved sequences, quality filtered	sample code	percentage of reads mapped to the reference genome	number of quality-filtered and deduplicated read pairs
**other included species**							
E. asperulum							
PTSK					PTSK1	92.20%	5515490
MTCH					MTCH1	92.34%	6260017
KRKL					KRKL1	92.40%	3678591
E. bulgaricum							
BALG					BALT1	83.97%	6572087
E. calycinum							
Drite					DRITE1	91.19%	4876391
					DRITE2	91.71%	2248110
LPSH					LPSH	93.14%	2596429
IZVR	IZVR1	58.82%	685015	914	IZVR1	92.29%	2760565
Tajmiste					Tajmiste2	89.13%	2357047
Botun					BOTUN1	90.76%	1948040
					BOTUN2	90.07%	5124815
NovakE2					Novak1_Ery2_1	91.37%	2623562
E. cephalonicum							
KSTR					KSTR1	92.20%	4253530
MTMA	MTMA1	55.30%	772984	912	MTMA1	92.05%	3906331
VMPS					VMPS1	91.51%	4856913
PRIL					PRIL1	91.47%	6287879
MOUS					MOUS2	91.57%	6416282
E. comatum							
ZvezdeP11					ZvezdeP11	91.53%	4835260
CAKR	CAKR14	52.47%	605405	914	CAKR14	89.50%	1094224
GLCC					GLCC1	91.49%	9190926
GalicicaEry2					GalicicaEry2_1	87.20%	2995602
MarkoviKuli					MarkoviKuli1	91.93%	4318105
CHEIA					CHEIA1sSax	89.94%	1552282
BUILA	BUILA1s	52.82%	536434	914	BUILA1s	74.06%	8835912
HER					HER1s	92.58%	9896674
E. cf cuspidatum							
BAND					Band2	90.09%	4496738
E. cuspidatum							
GLCH					GLCH2	92.23%	6947377
SPSH					SPSH1	89.81%	6688607
PEC					PEC1s	91.64%	5477476
					PEC2s	91.83%	5303758
SVIcus	SVI9s	55.62%	647063	914	SVI9s	92.74%	2905196
cusBRUS					BRUScusp1	85.18%	5415012
Hadic					Hadic1	92.12%	5201619
KONJ	KONJ14	55.39%	834552	914	KONJ_21	88.18%	2524404
OzrenCusp					OzrenCusp	91.92%	4089463
***E. diffusum* s.l. (*E. crassistylum*)**							
PTL					663PTL1	91.77%	9285518
RDU	652RDU2	55.25%	562340	912	652RDU2	91.61%	10489666
***E. diffusum s.l*. (*E. cf pirinicum*)**							
ORLK					ORLK5	91.69%	4561545
***E. diffusum* s.l. (*E. cf welchevii*)**							
Nise					Nise1	90.44%	5006210
Vrelo					Vrelo1	91.92%	5038797
***E. diffusum* s.l.**							
SVI					SVI2s	88.89%	4606521

**Population code**	**Hyb-Seq**				**RADseq**		
	sample code	percentage of reads mapped to the targeted sequences	number of quality-filtered and deduplicated read pairs	number of retrieved sequences, quality filtered	sample code	percentage of reads mapped to the reference genome	number of quality-filtered and deduplicated read pairs
					SVI3s	90.58%	22815642
					SVI7s	92.64%	3630339
COR					COR1s	91.67%	3864362
					COR2s	90.97%	4856324
					COR3s	91.39%	2260562
					COR4s	90.42%	3773362
BREH					BREH1	85.98%	1707724
LEV	LEV2	56.06%	700528	913	LEV2	90.03%	3163590
***E. diffusum* s.l. (*E. cf canum*)**							
DANK					DANK1	89.21%	4491317
GAB					GAB7	88.44%	3583637
CERN					CERN1	92.05%	4426055
E. graecum							
KRIA					KRIA1	92.01%	5834730
SLLS					SLLS1	92.25%	8858747
SKOT					SKOT1	92.79%	7793422
ACHR	ACHR1	55.14%	479626	913	ACHR1	92.59%	7110347
E. hayekii							
PHTS					PHTS1	91.24%	8006563
E. horizontale							
LESB					LESB1	92.26%	14224594
E. cheiranthoides							
Balaze					Balaze4	94.92%	7755084
GAJ					GAJ1	97.43%	2624315
E. cf janchenii							
Ozren	Ozren1	53.11%	552912	914	Ozren1	90.63%	3596622
	Ozren2R	57.79%	954829	914			
E. linariifolium							
TomorritT2					TomorritT2	89.97%	11104284
Gacko					Gacko1	91.47%	3890537
STOZ					STOZ3	92.33%	14798028
GORPO					Gopro1	85.49%	5603951
TUSI					Tusi1	90.99%	2780229
linKavlar	Kavlar1lin	56.05%	645150	914	Kavlar1linarif	82.23%	14409782
DUCA	DUCA8	54.53%	622648	914	DUCA8	90.61%	3996269
E. microstylum							
Alona	Alona1	55.60%	788476	914	Alona1	91.17%	4094206
E. parnassi							
Gero	GERO1	55.26%	642110	914	GERO1	91.68%	5605836
	GERO2	55.25%	743270	913			
E. pseudocuspidatum							
MNLN					MNLN1	92.66%	4891075
Vrondero	Vrondero3	54.35%	686683	913	Vrondero3	90.93%	3394871
					Vrondero4	91.58%	8577153
E. pseudorhaeticum							
642CAT2					642CAT2	91.29%	13085841
639CLB2	639CLB2	53.69%	735266	914	639CLB2	90.99%	2065585
640SIV2					640SIV2	90.00%	5116497
Vent					VENT10	85.64%	3596217
E. pusillum							
PROF	PROF1R	54.42%	616709	914	PROF1R	90.99%	6894645
E. rhaeticum							
PFDS					PFDS21	91.49%	5074391
					PFDS22R	89.82%	3481729

Population code	Hyb-Seq				RADseq		
	sample code	percentage of reads mapped to the targeted sequences	number of quality-filtered and deduplicated read pairs	number of retrieved sequences, quality filtered	sample code	percentage of reads mapped to the reference genome	number of quality-filtered and deduplicated read pairs
CDLA					CDLA1R	93.93%	4355527
GRNG					GRNG2	92.00%	6451509
LLDN					LLDN2	90.34%	5416144
BRUS					BRUS1	92.21%	2144444
JOLI					JOLI1	91.82%	6650388
E. smyrnaeum							
AGDI					AGDI1	89.60%	4217618
DKES					DKES1	90.38%	10518587
***E. sylvestre* auct.**							
Dovlici	Dovlici1	55.68%	513575	914	Dovlici1	85.32%	1438275
Sab/Sabotin	Sabotin11	53.14%	1250979	913	SABOTIN11	84.35%	2037773
***Malcolmia graeca* (outgroup)**							
Malcolmia_II	MalcolmiaII1	43.56%	419408	899	MalcolmiaII1	64.39%	3808684
Malcolmia_I	MalcolmiaI1	44.72%	484646	900	MalcolmiaI1	63.69%	8457535

Table S3. Assignment of alleles obtained from phased exon sequences (Hyb-Seq data) in the polyploids of *Erysimum croaticum* (*croa*, Croatia, Bosnia and Herzegovina), *E. croaticum* auct. (*croa* auct, Kosovo), and tetraploid populations from Montenegro (VRMAC and OBGL) to diploid species using the EPA-ng algorithm. Only the allelic placements with likelihood weight ratio greater than 0.9 were retained. Numbers in bold highlight the percentage values above 15%, which was used as the filtering threshold for subgenomic tree construction. Population codes follow Table S1.

Species	Accession (pop. code)	Allele types obtained (%)											Total No of alleles classified	Total No of alleles classified [%]	Total No of alleles
		carniolicum	*crassistylum*	*kuemmerlei*	*linariifolium*	*microstylum*	Pop. Ovčar	*pseudorhaeticum*	*pusillum*	*comatum*	*sylvestre* auct	*vitekii*			
croa	GBre1	4.71%	8.72%	4.56%	**20.74%**	6.21%	4.63%	7.93%	5.89%	7.93%	5.18%	9.82%	1273	23.21%	5484
croa	Omis4	5.64%	10.66%	4.34%	**21.10%**	5.79%	5.64%	7.84%	6.78%	7.31%	4.49%	8.23%	1313	23.94%	5484
croa	Omis18	6.59%	11.21%	4.18%	**21.54%**	6.15%	6.01%	7.25%	6.45%	6.52%	6.37%	7.03%	1365	24.92%	5478
croa	Stipan.4	3.78%	9.19%	5.33%	**17.99%**	5.56%	4.79%	8.49%	8.26%	7.34%	4.79%	8.65%	1295	23.61%	5484
croa auct	GLSH1	3.56%	10.07%	3.49%	13.97%	4.93%	3.84%	4.18%	3.42%	**28.70%**	5.41%	3.90%	1460	26.62%	5484
croa auct	GLSH4	4.22%	9.87%	3.74%	14.50%	5.79%	3.40%	5.04%	3.95%	**27.50%**	4.63%	3.61%	1469	26.79%	5484
croa auct	PRIZ1	4.12%	6.95%	5.35%	10.02%	4.06%	5.47%	2.95%	5.78%	**34.81%**	5.10%	5.10%	1626	29.65%	5484
croa auct	PRIZ15	2.63%	7.32%	5.72%	14.45%	4.43%	4.11%	3.15%	4.95%	**33.98%**	7.06%	3.40%	1557	28.42%	5478
	OBGL13	**20.41%**	1.80%	5.63%	3.18%	1.14%	8.65%	2.12%	2.12%	3.76%	7.35%	**33.22%**	1225	33.51%	3656
	VRMAC2	**24.12%**	2.50%	6.03%	4.05%	1.46%	7.84%	2.67%	2.58%	3.70%	7.49%	**29.46%**	1161	31.79%	3652

Table S3 continued (all classifications below 5%):

Species	Accession (pop. code)	Allele types obtained (%)						Total No of alleles classi-fied	Total No of alleles classi-fied [%]	Total No of alleles
		cuspidatum	*parnassi*	*pectinatum*	*pseudocuspidatum*	*witmannii*	*Malcolmia*			
croa	GBre1	1.89%	3.14%	2.59%	2.91%	2.99%	0.16%	1273	23.21%	5484
croa	Omis4	1.90%	2.89%	1.68%	1.14%	4.34%	0.23%	1313	23.94%	5484
croa	Omis18	0.88%	3.30%	2.05%	1.32%	2.86%	0.29%	1365	24.92%	5478
croa	Stipan.4	1.93%	3.55%	3.09%	2.32%	4.86%	0.08%	1295	23.61%	5484
croa auct	GLSH1	4.32%	2.74%	1.71%	2.12%	3.49%	0.14%	1460	26.62%	5484
croa auct	GLSH4	2.79%	2.93%	2.04%	2.18%	3.68%	0.14%	1469	26.79%	5484
croa auct	PRIZ1	1.60%	3.32%	1.85%	1.05%	2.46%	0.00%	1626	29.65%	5484
croa auct	PRIZ15	1.54%	1.54%	1.41%	0.90%	3.02%	0.39%	1557	28.42%	5478
	OBGL13	1.22%	1.88%	1.39%	0.98%	4.57%	0.57%	1225	33.51%	3656
	VRMAC2	0.86%	1.81%	0.95%	0.69%	3.62%	0.17%	1161	31.79%	3652

Table S4 Variation in trichome types on petals, stems, leaves, and, when available, siliques in the studied *Erysimum* species. Values represent the proportion of individuals within each species or entity that possess a given trichome type (2-6-fid, i.e., bearing 2-6 rays). Note that multiple trichome types commonly co-occur on the same plant and organ. * refers to two populations from Montenegro (OBGL, VRMAC, see Table S1) with proven allotetraploid origin; ** *E. croaticum* refers to populations from Croatia and Bosnia and Herzegovina; *** *E. croaticum* auct. refers to a population from Kosovo.

**Species** (number of examined individuals, and populations)	**Trichomes on petals**					**Trichomes on stems**				**Trichomes on leaves**					**Trichomes on siliques**				
	none	2-fid	3-fid	4-fid	5-fid	2-fid	3-fid	4-fid	5-fid	2-fid	3-fid	4-fid	5-fid	6-fid	2-fid	3-fid	4-fid	5-fid	6-fid
E. carniolicum (n=193, 11 pops.)	0.58	0.13	0.28	0.07	0	1	0.70	0.03	0	0.78	1	0.91	0.18	0	0.82	0.99	0.67	0.08	0
E. vitekii (n=66, 4 pops.)	0.23	0.46	0.59	0.22	0	1	0.62	0.03	0	0.85	1	0.89	0.12	0	0.76	1	0.33	0	0
E. carniolicum x *E. vitekii** (n=35, 2 pops.)	0.56	0.44	0.06	0	0	1	0.60	0.03	0	0.86	1	0.91	0.14	0	1	1	0.16	0.03	0
E. kuemmerlei (n=112, 6 pops.)	0.15	0.16	0.53	0.51	0.20	0.99	0.92	0.13	0	0.29	0.90	0.99	0.89	0.15	0.05	0.85	0.97	0.81	0.13
E. pectinatum (n=40, 2 pops.)	0	0.58	1	0.08	0	1	0.30	0	0	0.9	1	0.4	0.03	0	1	1	0	0	0
population from Mt. Ovčar (n=28, 1 pop.)	0.11	0.11	0.43	0.75	0.36	0.86	1	0.29	0.04	0	0.68	1	1	0.25	0.04	0.93	1	0.89	0.07
*E. croaticum*** (n=62, 3 pops.)	0.69	0.29	0.05	0	0	1	0.05	0	0	1	0.39	0	0	0	1	0.36	0	0	0
E. croaticum auct.*** (n=17, 1 pop.)	0.65	0.29	0.18	0	0	1	0.18	0	0	1	0.47	0	0	0	1	0.75	0	0	0

Figure S1. Mitotic chromosomes of selected *Erysimum* accessions stained with DAPI and imaged under epifluorescence microscopy. Chromosome spreads were prepared either from root tips (indicated with ^{root} next to the accession code) or from anthers (indicated with ^{ant}). Population codes are as in Table S1, followed by the individual number (and, where applicable, chromosome plate identification codes in brackets). Scale bars: 5 µm.

Erysimum carniolicum 2*n*=12

CRNJ1^{ant} 2*n*=12

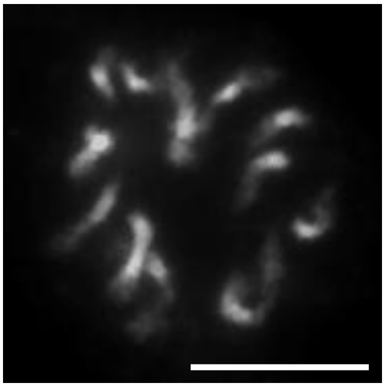

CRNJ4^{ant} 2*n*=12

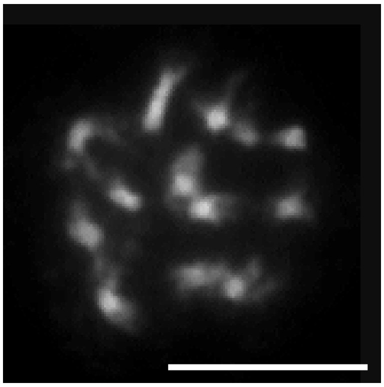

Durm^{ant} 2*n*=12

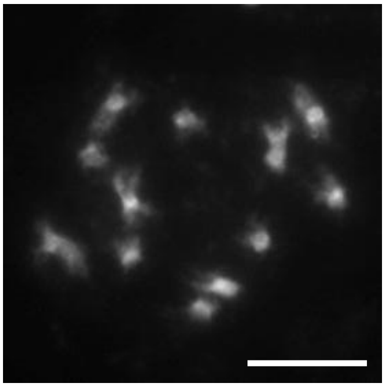

Kalnik11^{root} (5216) 2*n*=12

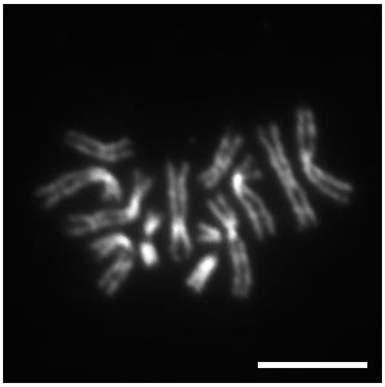

Kalnik12^{root} (5217) 2*n*=12

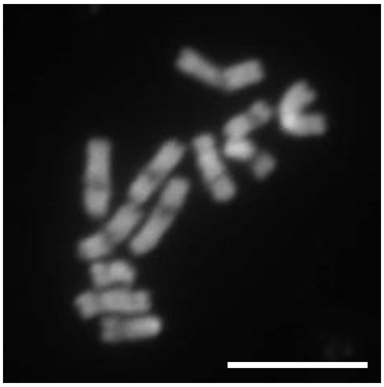

Kalnik12a^{ant} 2*n*=12

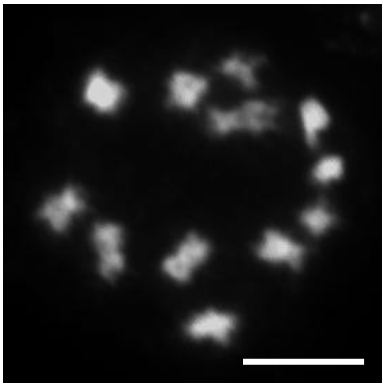

Kalnik11b-2^{ant} 2*n*=12

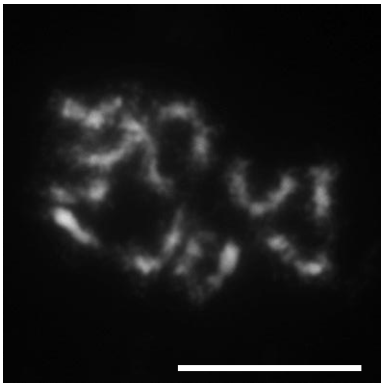

Kalnik11b^{ant} 2*n*=12

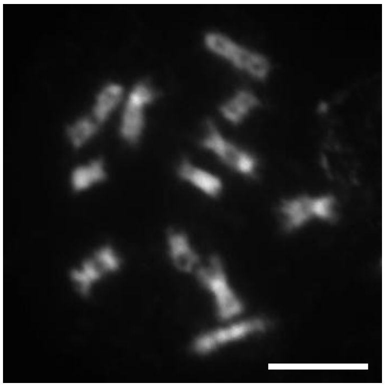

Kalnik12b-2^{ant} 2*n*=12

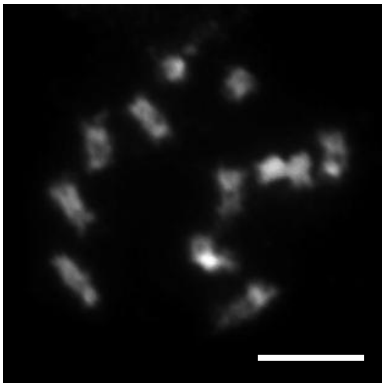

***Erysimum carniolicum* 2n=12**

Kalnik12-3^{ant} 2*n*=12

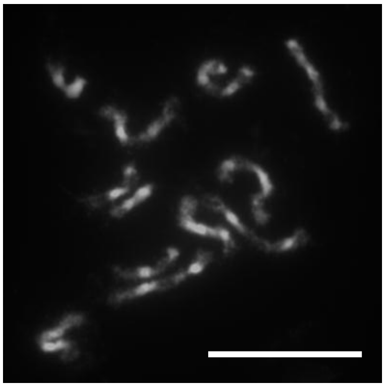

Kalnik12b^{ant} 2*n*=12

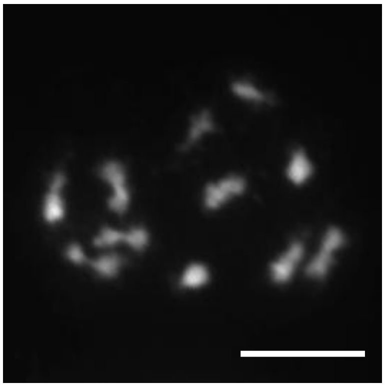

Podjelak11^{root} (5218) 2*n*=12

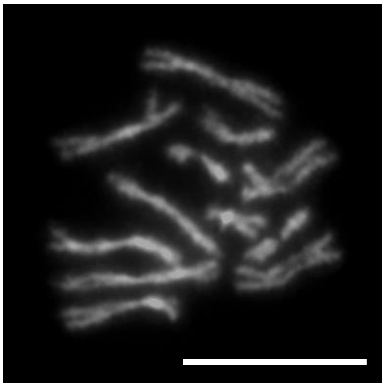

Podjelak12^{root} (5219) 2*n*=12

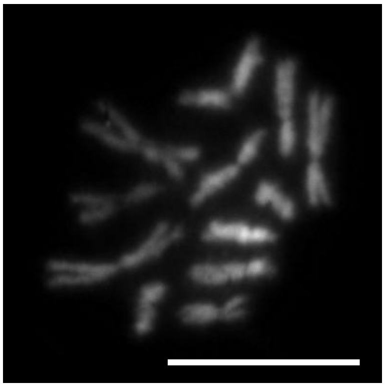

Podjelak12b^{ant} 2*n*=12

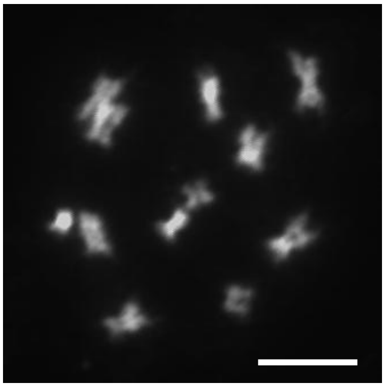

KPNK23/5^{root} (5606) 2*n*=12

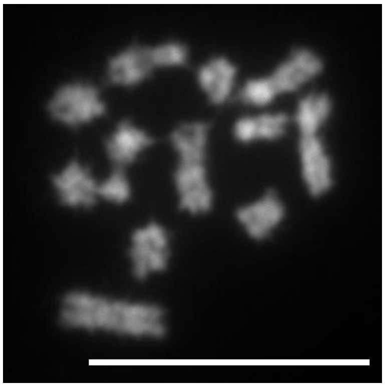

KPNK23/1^{root} (5604) 2*n*=12

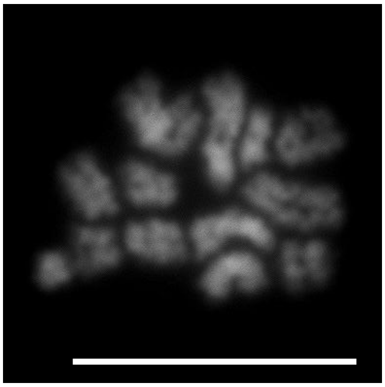

KPNK23/2^{root} (5605) 2*n*=12

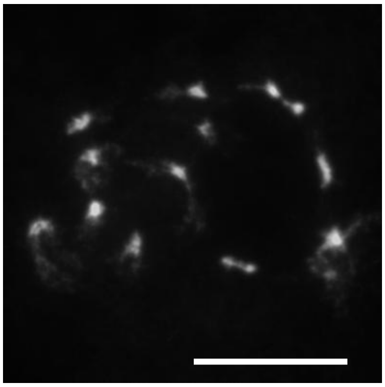

Svibno^{ant} (5603) 2*n*=12

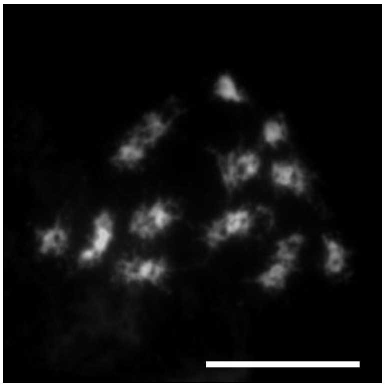

Erysimum carniolicum 2*n*=16

Jasik24/6 root (6408) 2*n*=16

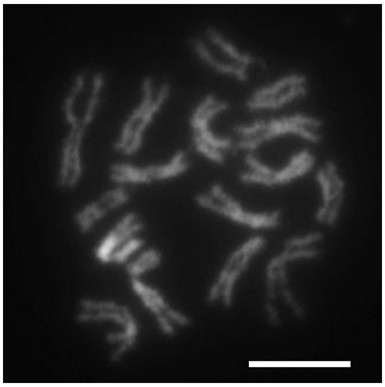

Jasik24/4root (6406) 2*n*=16

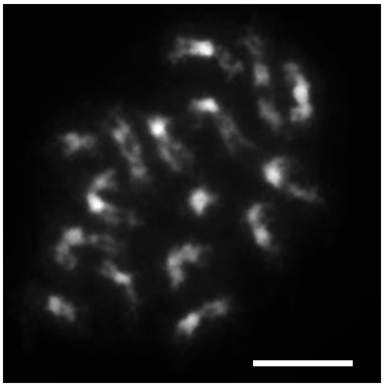

Jasik24/5root (6407) 2*n*=16

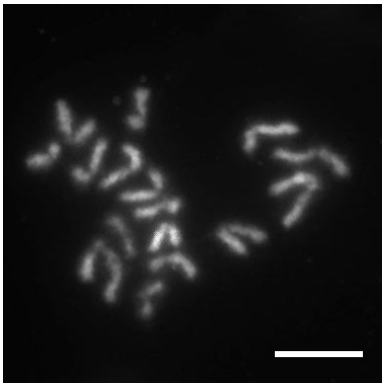

MGLC24/4root (6411) 2*n*=16

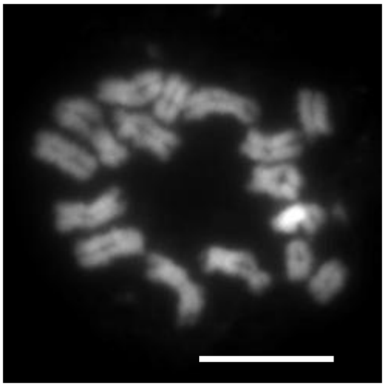

MGLC24/1root (6409) 2*n*=16

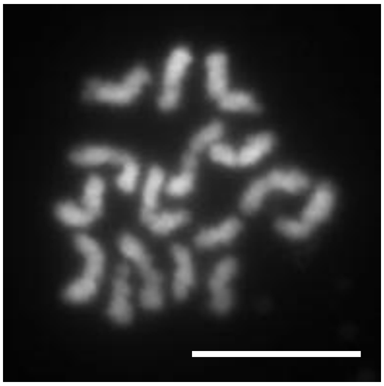

MGLC24/1root (6410) 2*n*=16

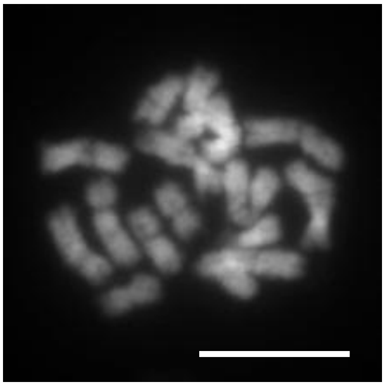

Erysimum carniolicum 2*n*=18, 2*n*=30

ZLAT4ant 2*n*=18

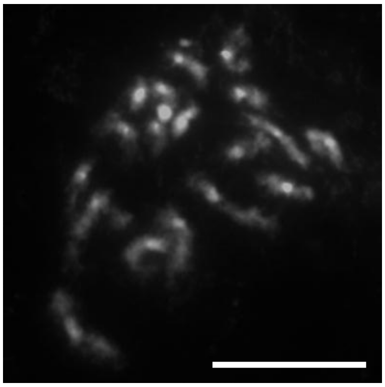

ZLAT2 ant 2*n*=30

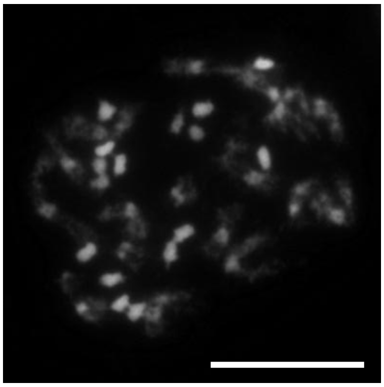

Erysimum kuemmerlei 2*n*=12

GJLS3^{ant} 2*n*=12

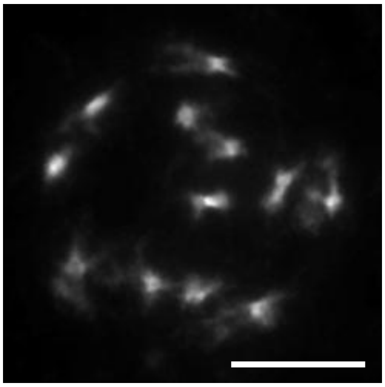

GJLS2^{ant} 2*n*=12

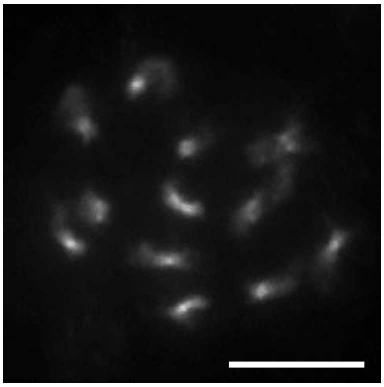

MVRV24/1^{root} (6403) 2*n*=12

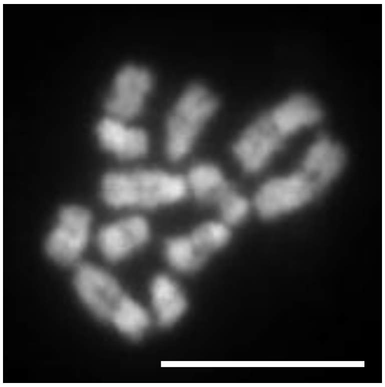

MVRV24/5^{root} (6405) 2*n*=12

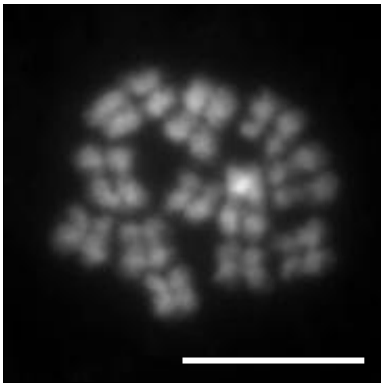

MVRV24/9^{root} (6404) 2*n*=12

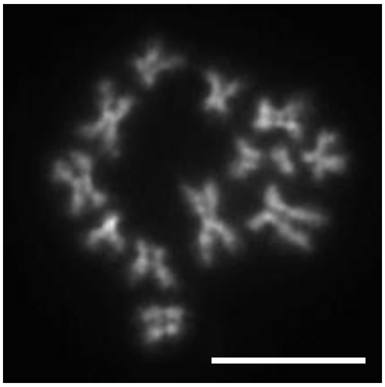

PRST2p ^{ant} 2*n*=12

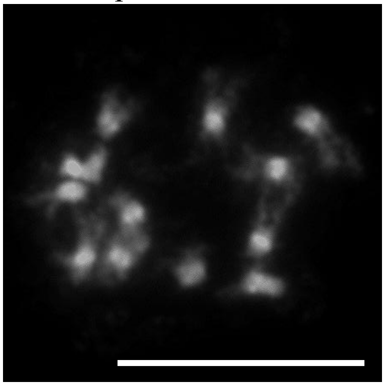

PRST5p ^{ant} 2*n*=12

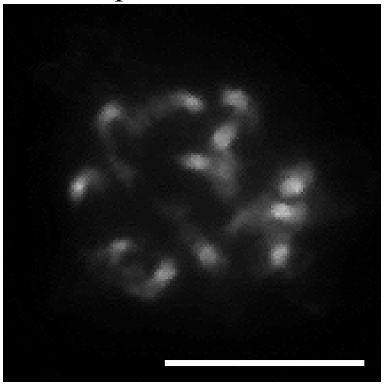

PRST6p ^{ant} 2*n*=12

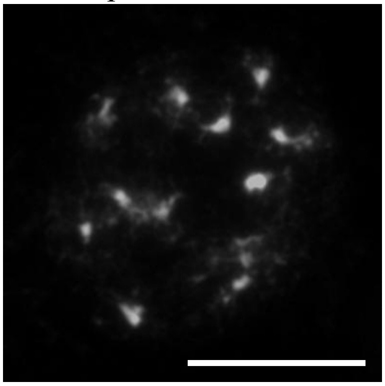

SHTR1 ^{ant} 2*n*=12

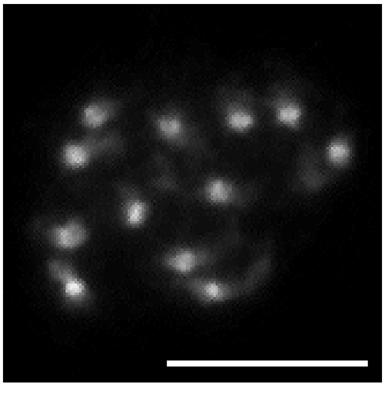

SHTR3 ^{ant} 2*n*=12

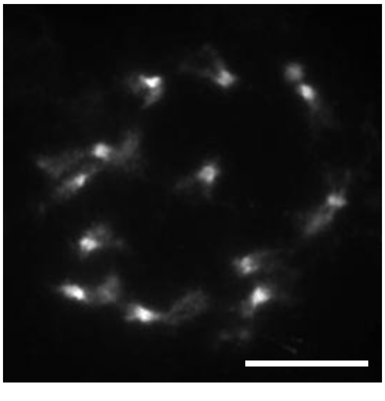

Erysimum kuemmerlei 2*n*=14

GLCA24/1^{root} (6412) 2*n*=14

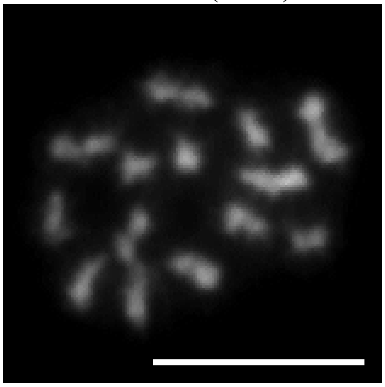

GLCA24/15^{root} (6414) 2*n*=14

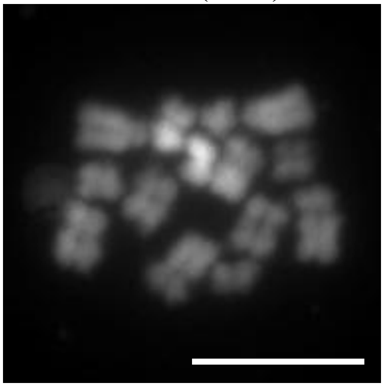

GLCA24/2^{root} (6413) 2*n*=14

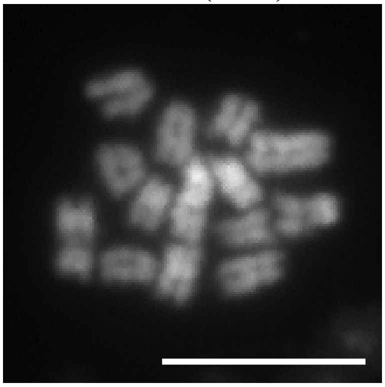

Taxonomically unassigned population from Mt. Ovčar, Serbia, 2*n*=12

OVCA11^{ant} (5602) 2*n*=12

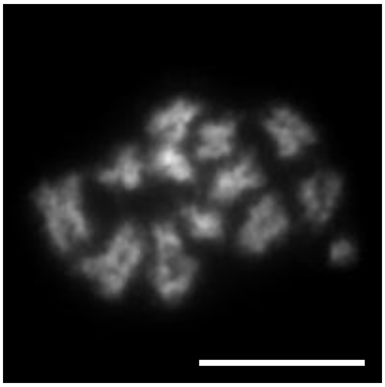

OVCA2^{root} (5598) 2*n*=12

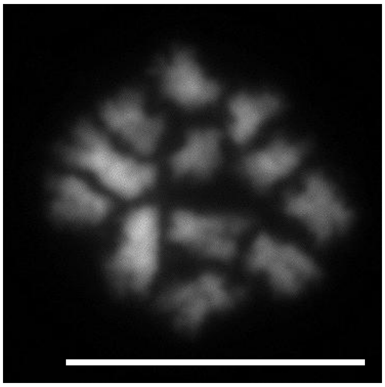

OVCA3^{root} (5599) 2*n*=12

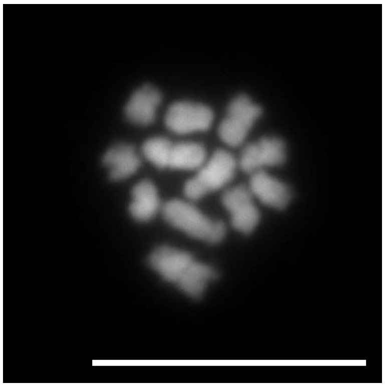

OVCA6^{ant} (5600) 2*n*=12

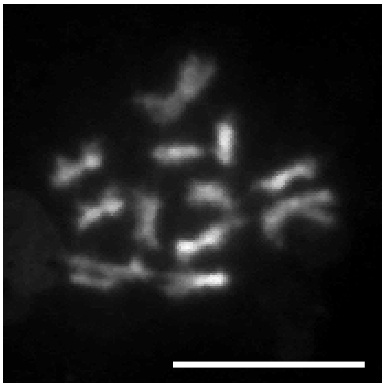

OVCA7^{root} (5601) 2*n*=12

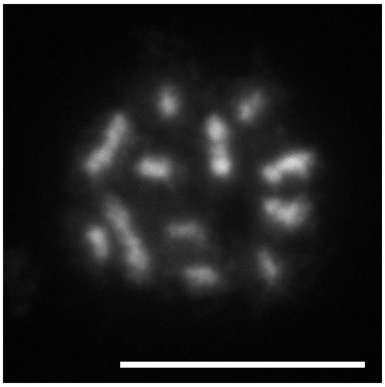

OVCA18^{ant} 2*n*=12

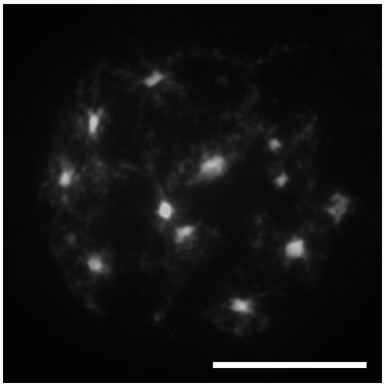

***Erysimum pectinatum* 2*n*=12**

VMVK2^{ant} 2*n*=12

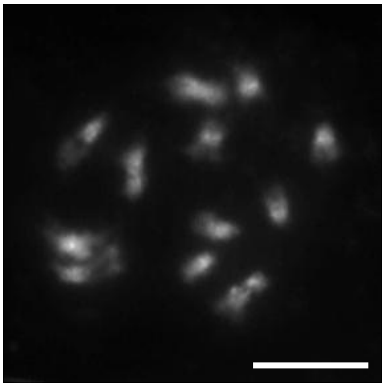

VMVK4^{ant} 2*n*=12

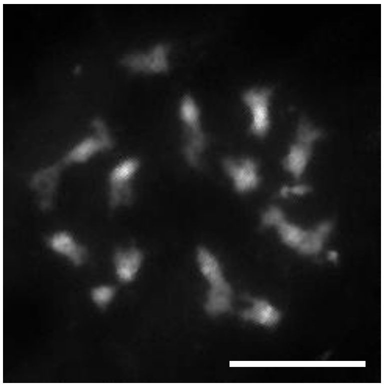

ELAT1^{ant} 2*n*=12

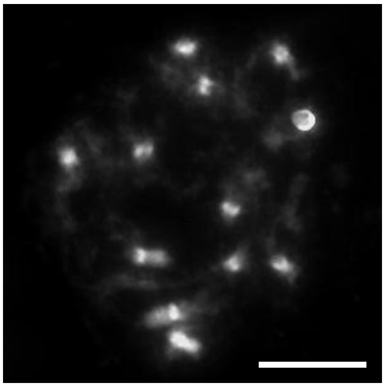

ELAT3^{ant} 2*n*=12

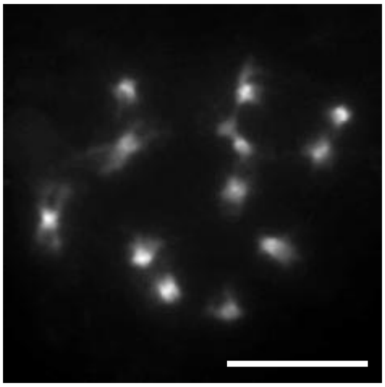

TGTS3^{ant} 2*n*=12

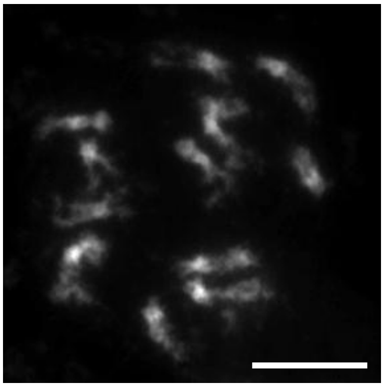

TGTS1^{ant} 2*n*=12

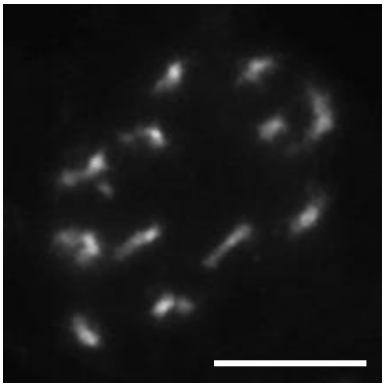

***Erysimum pectinatum* 2*n*=16**

PTER1^{ant} 2*n*=16

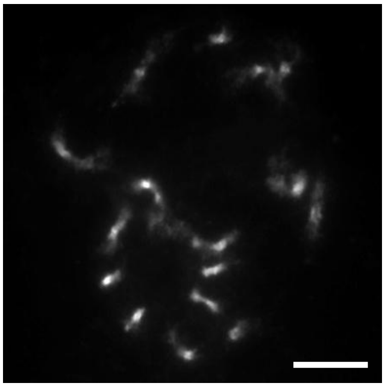

PTER5^{ant} 2*n*=16

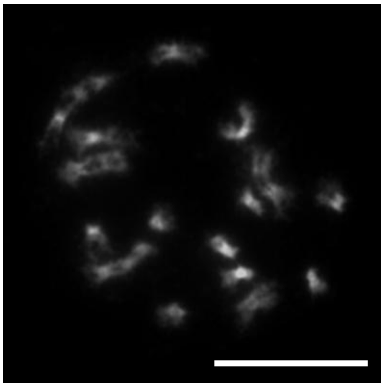

***Erysimum pectinatum* 2*n*=22**

MSVN1^{ant} 2*n*=22

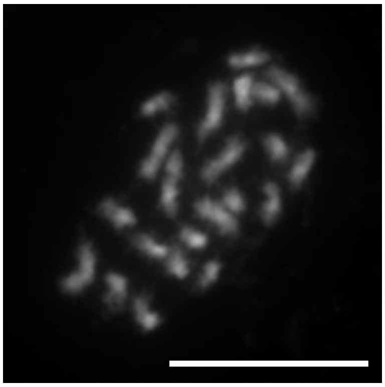

MSVN6^{ant} 2*n*=22

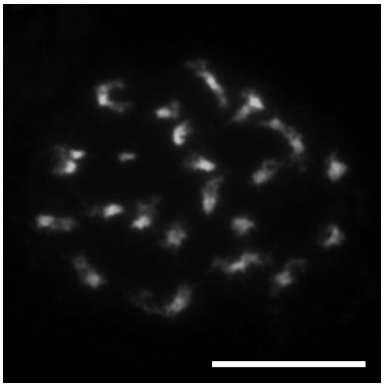

Erysimum croaticum 2*n*=30

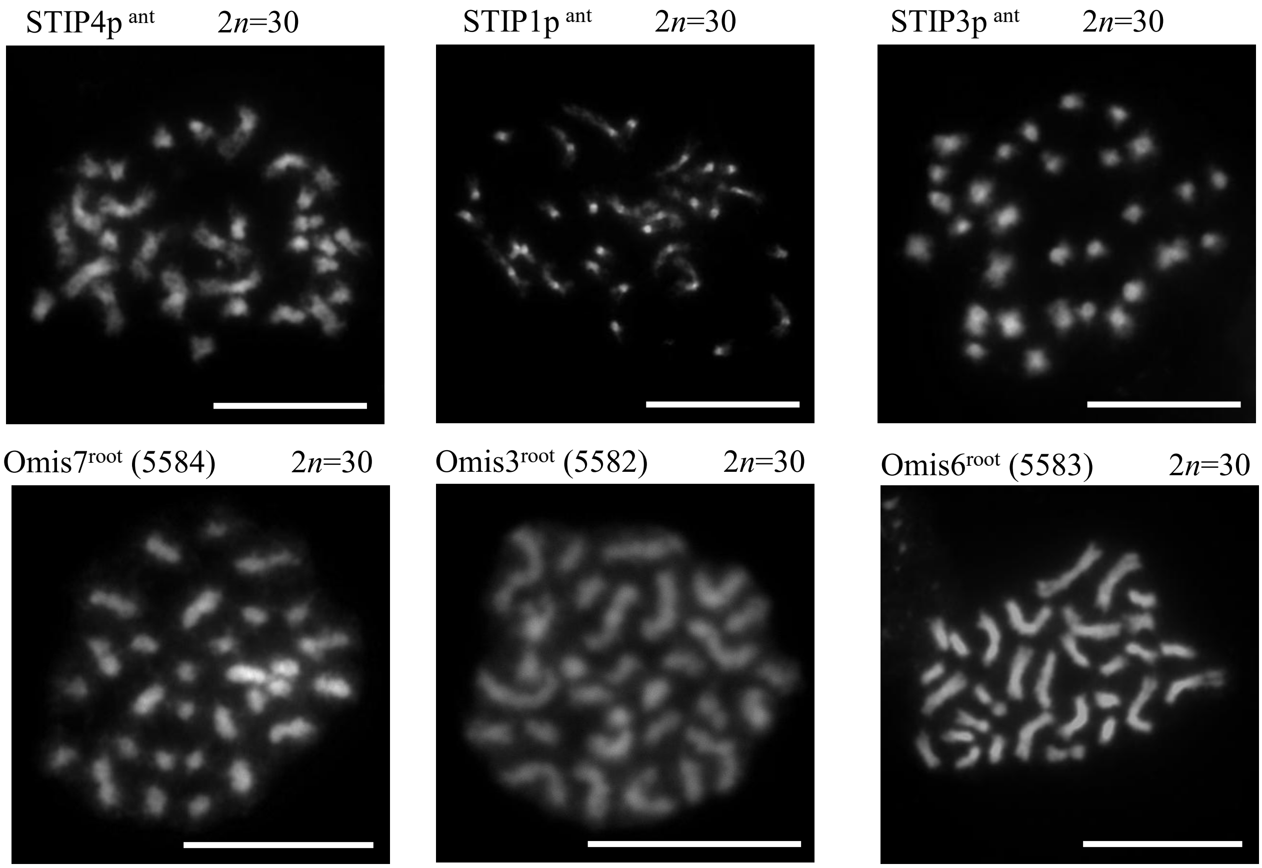

Erysimum croaticum 2*n*=36, 2*n*=38, 2*n*=40

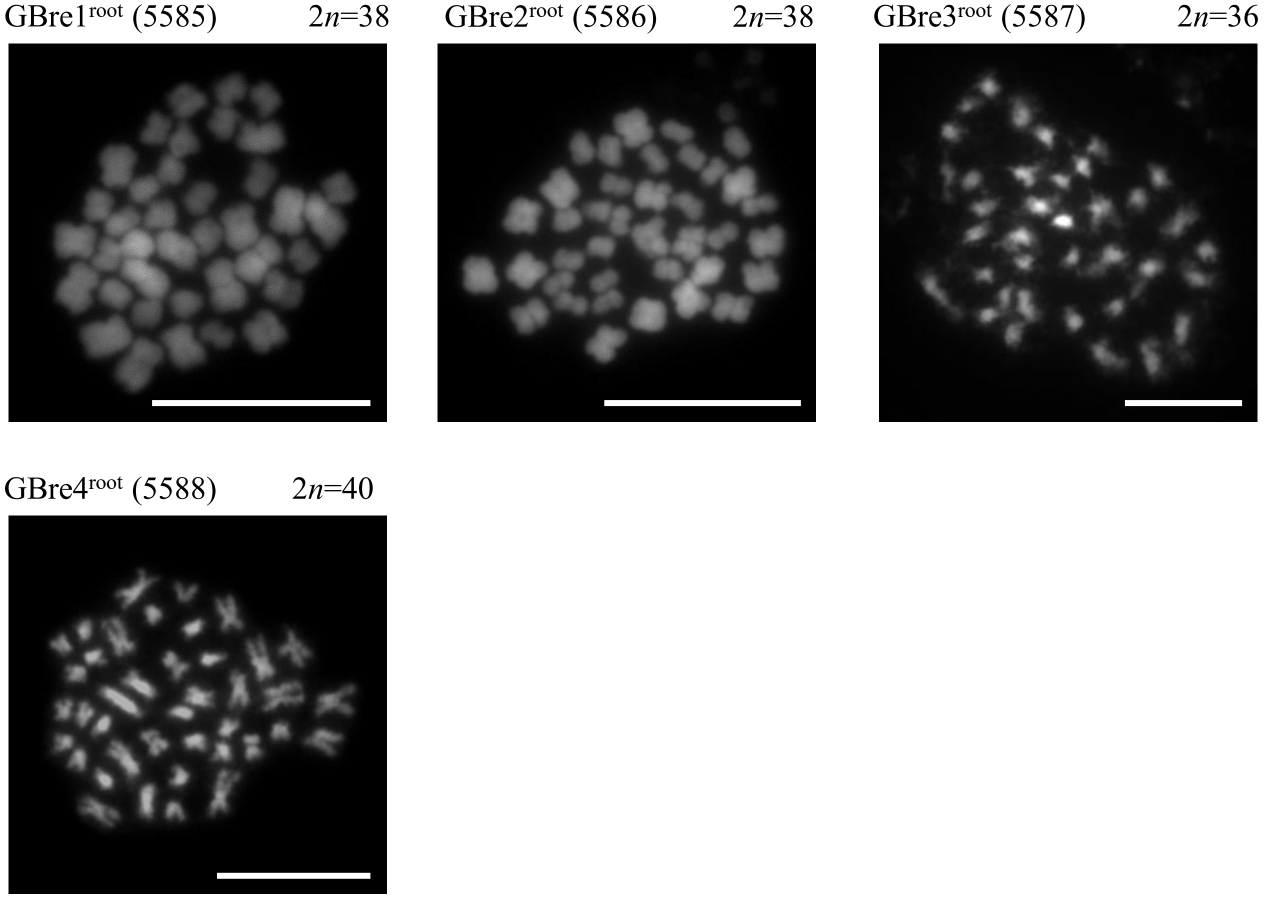

Erysimum croaticum **auct. 2*n*=36**

Prizren1^{root} (5162) 2*n*=36

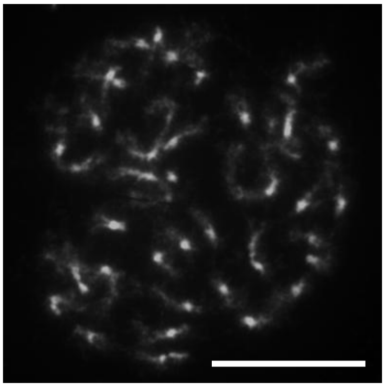

Prizren2^{root} (5163) 2*n*=36

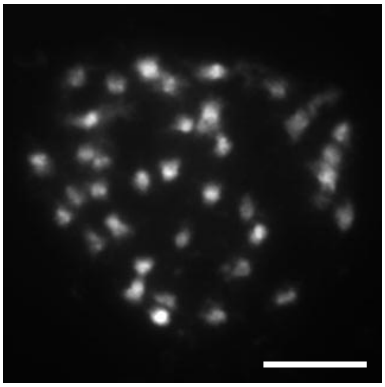

Prizren4^{root} (5164) 2*n*=36

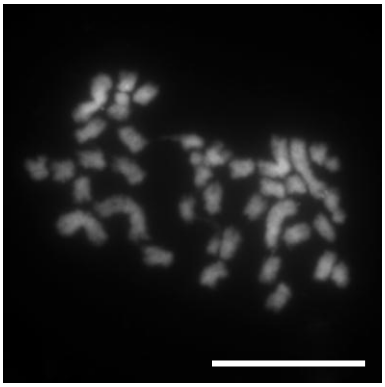

Prizren 5^{ant} 2*n*=36

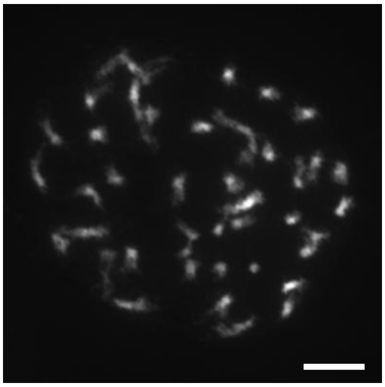

Prizren 6^{ant} 2*n*=36

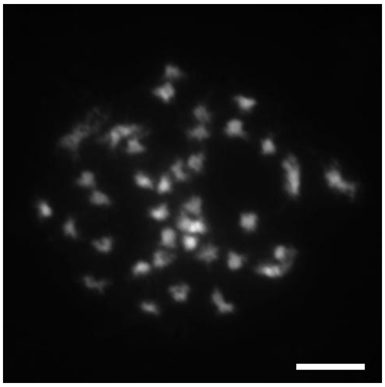

Prizren 13^{ant} 2*n*=36

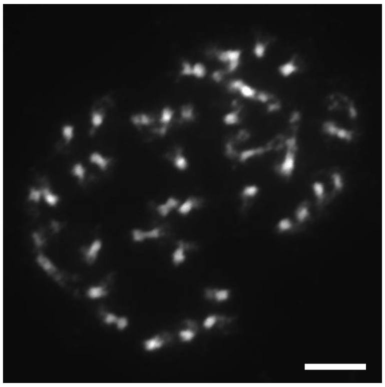

Prizren 11^{ant} 2*n*=36

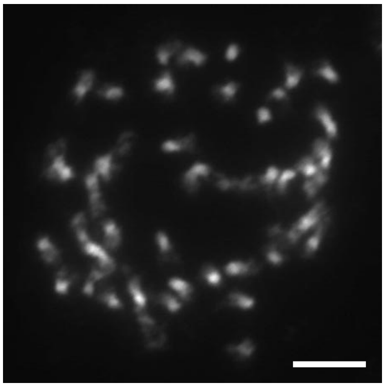

Erysimum vitekii 2*n*=12

CRKV^{root}2 (5595) 2*n*=12

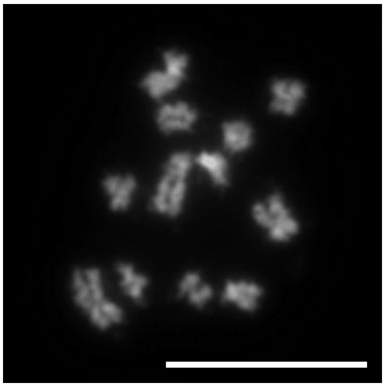

CRKV3^{root} (5596) 2*n*=12

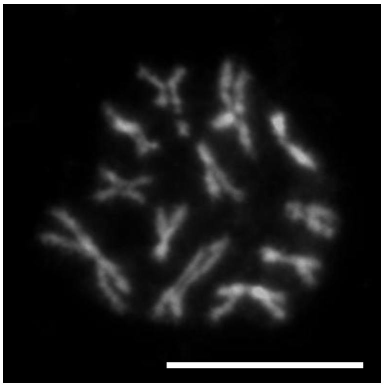

CRKV4^{root} (5597) 2*n*=12

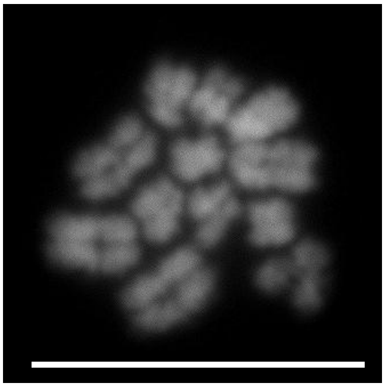

MRCC2^{root} (5589) 2*n*=12

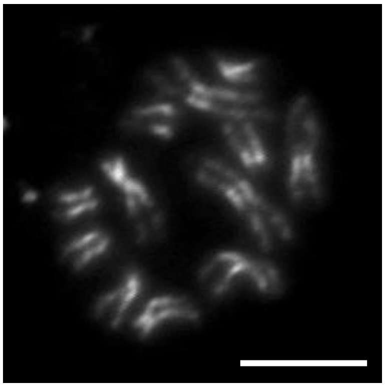

MRCC3^{root} (5590) 2*n*=12

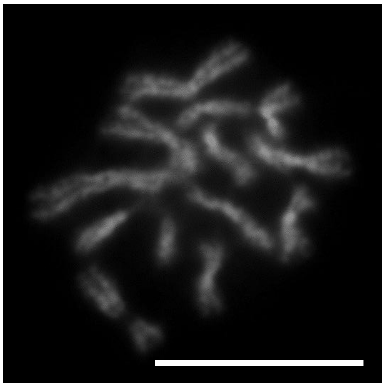

MRCC4^{root} (5591) 2*n*=12

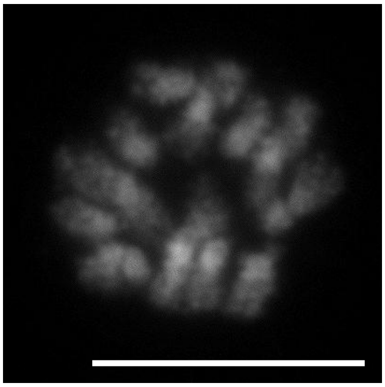

MRCC5^{root} (5592) 2*n*=12

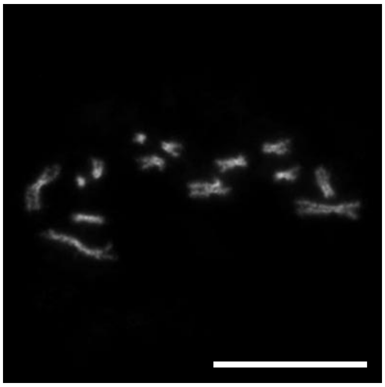

MRCC6^{root} (5593) 2*n*=12

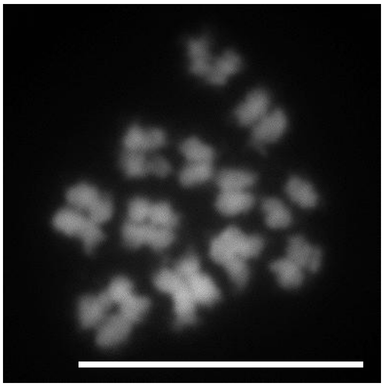

MRCC7^{root} (5594) 2*n*=12

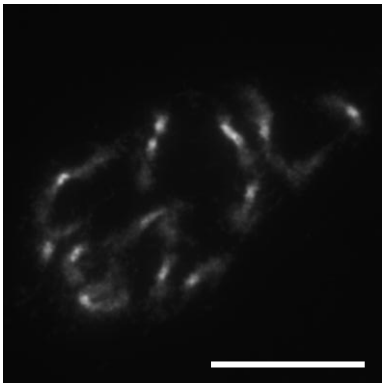

NEVES24/3^{root} (6421) 2*n*=12

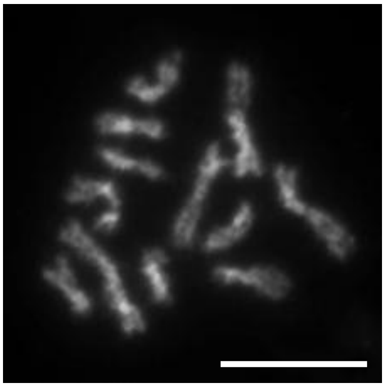

NEVES24/7^{root} (6422) 2*n*=12

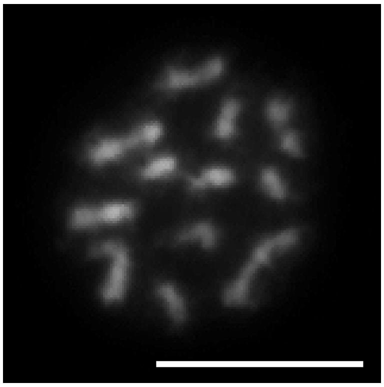

NEVES24/8^{root} (6423) 2*n*=12

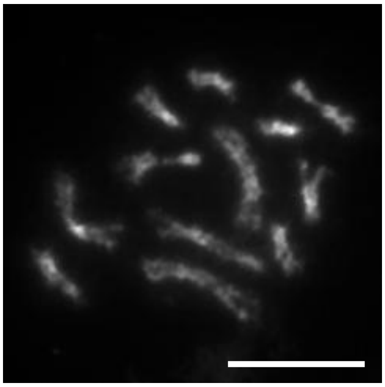

***Erysimum vitekii* 2*n*=14**

Podvranic24/7^{root} (6425) 2*n*=14

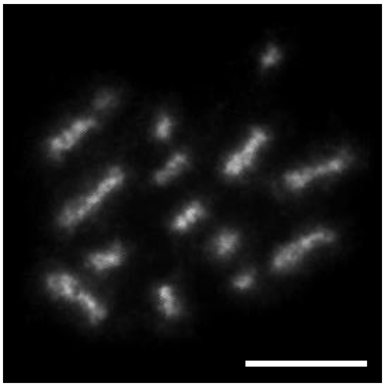

Podvranic24/9^{root} (6426) 2*n*=14

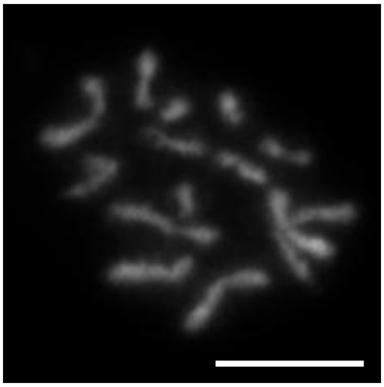

Podvranic24/5^{root} (6424) 2*n*=14

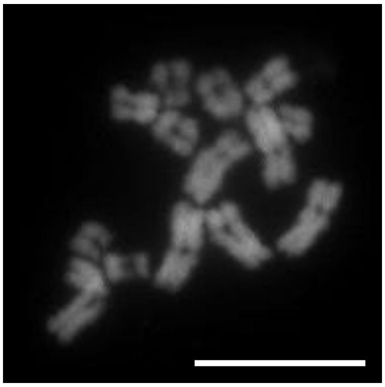

Rilja ^{ant} 2*n*=14

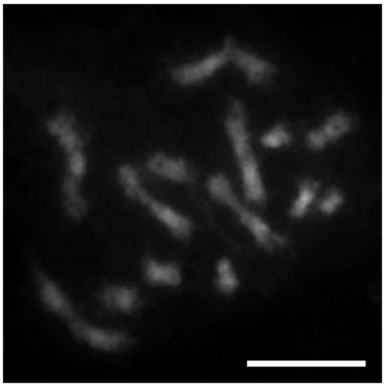

Rilja2 ^{ant} 2*n*=14

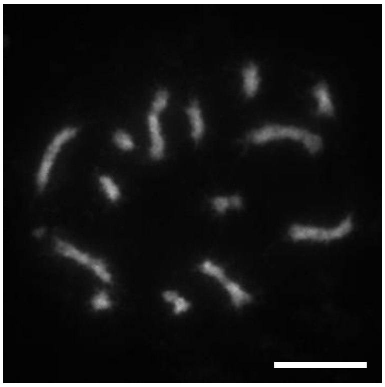

Erysimum carniolicum x *Erysimum vitekii* **2*n*=20-24**

OBGL4 ^{ant} 2*n*=20

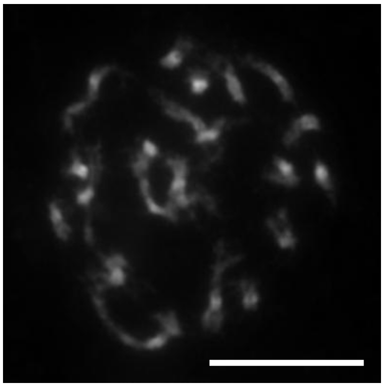

OBGL1 ^{ant} 2*n*=22

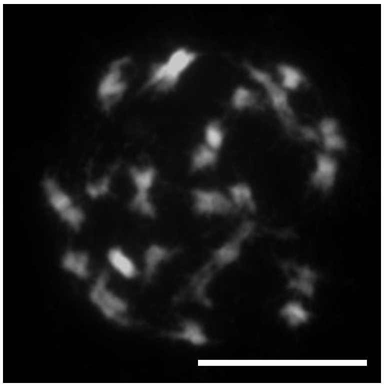

OBGL24/7^{root} (6420) 2*n*=22

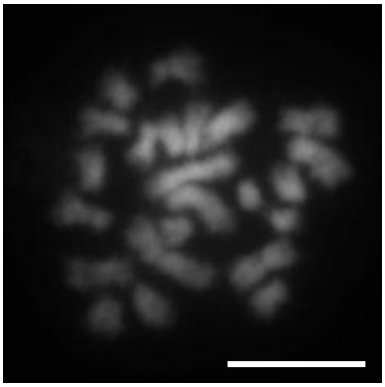

OBGL24/1^{root} (6418) 2*n*=22

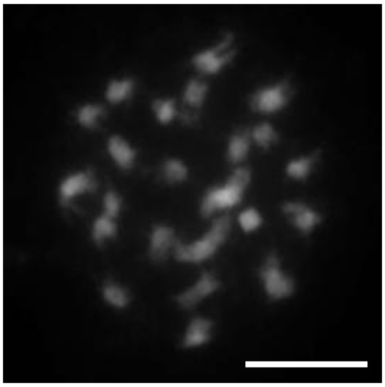

VRMAC24/3 ^{ant} (6415), 2*n*=22

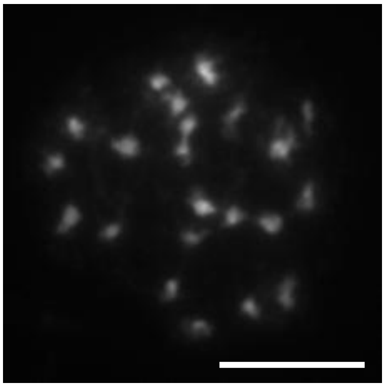

VRMAC2 ^{ant} 2*n*=22

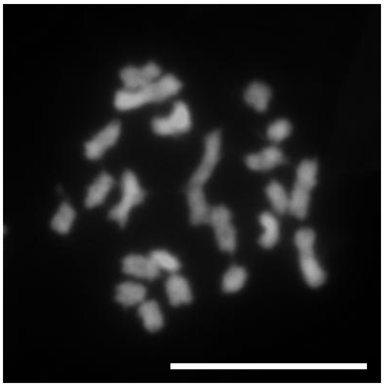

VRMAC3 ^{ant} 2*n*=22

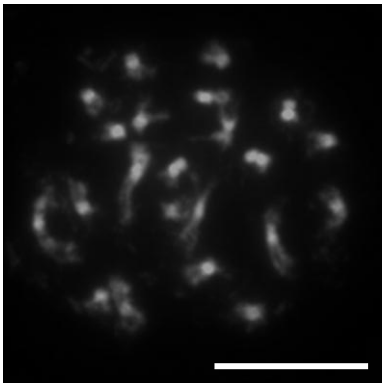

VRMAC24/6^{root} (6417) 2*n*=24

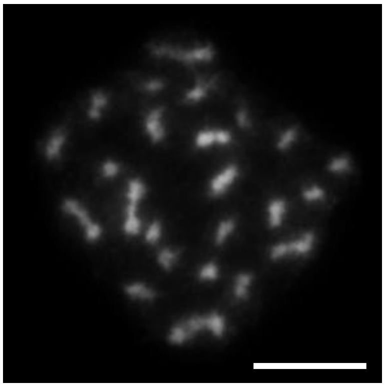

VRMAC24/5^{root} (6416) 2*n*=24

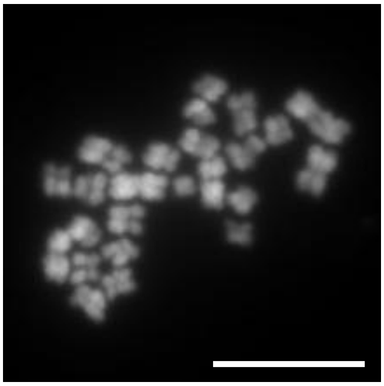

Figure S2 (A) Maximum-likelihood tree inferred in RAxML based on RADseq data. Branch support (quartet concordance symbols in the nodes, quartet concordance/quartet differential/quartet informativeness values below the branches, bootstrap values above the branches) is shown, except for the terminal intraspecific clades. Population codes follow Table S1.
(B) NeighbourNet analysis based on Nei's genetic distance of RADseq data.

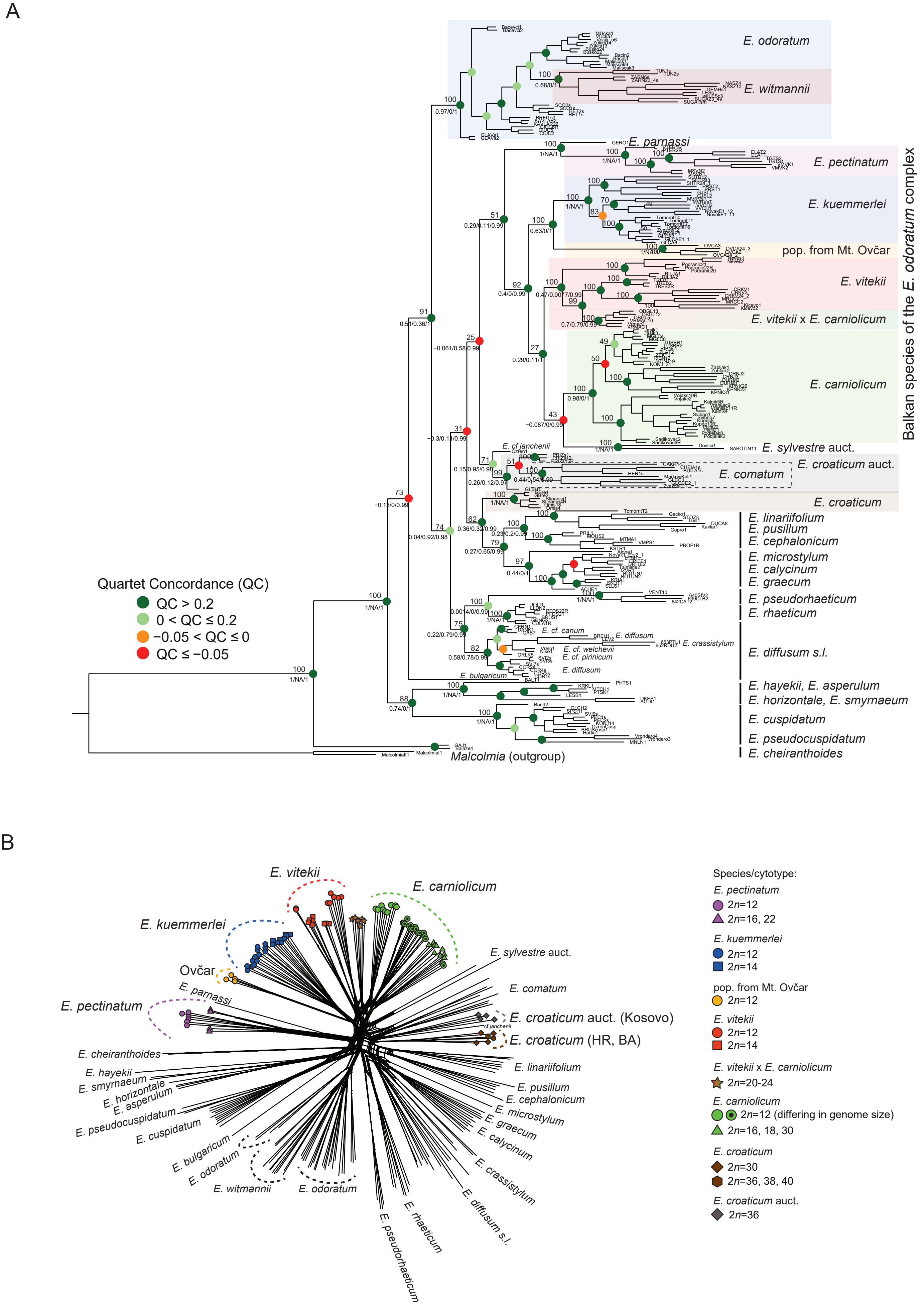

Figure S3 Violin plots showing the distribution of pairwise relatedness values between the polyploids *E. croaticum* (Croatia, Bosnia and Herzegovina) and *E. croaticum* auct. (Kosovo) and all other sampled species, computed from RADseq data using the PolyRelatedness tool. The boxes within the violin plots represent standard deviation, the horizontal lines within the boxes are mean values, and the red circles are median values.

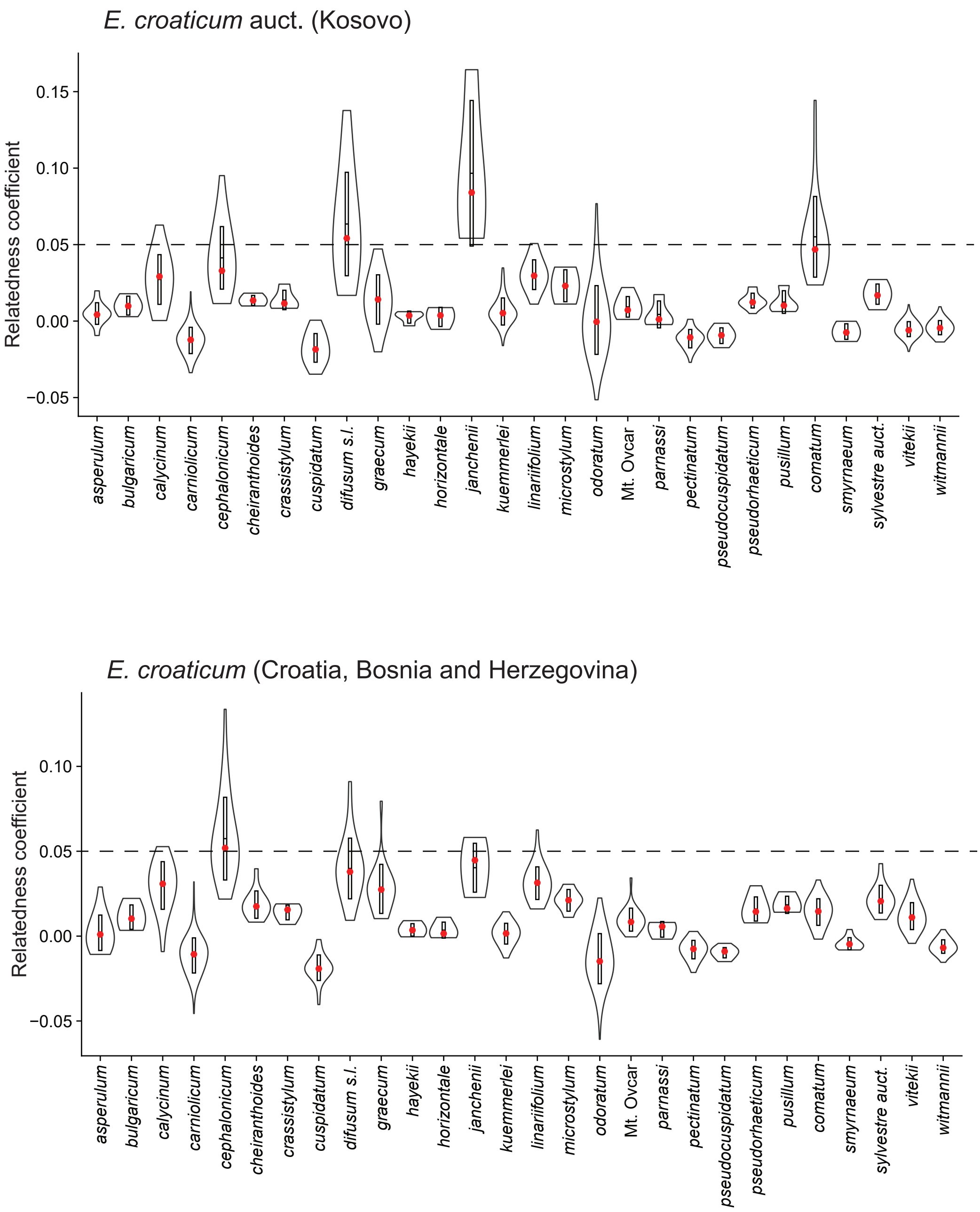

Figure S4 Results of f-branch analysis computed from RADseq data in Dsuite for the clade comprising the target species, *E. carniolicum, E. vitekii, E. kuemmerlei, E. pectinatum*, a distinct entity (taxonomically unassigned) from Mt. Ovčar, further including *E. sylvestre* auct. and *E. parnassi* (the clade as delimited in Fig. 3A, 3B), but excluding the proven allotetraploids (*E. vitekii* x *E. carniolicum*, accessions OBGL and VRMAC). The input tree was inferred in ASTRAL-III, summarizing 7168 maximum-likelihood gene trees constructed from RADseq loci at least 80 bp long and comprising at least six phylogenetically informative sites (7168 loci in total). Branches with low support (LPP < 0.8) are depicted with dotted lines. In the chart, coloured squares indicate a significant excess of SNPs sharing between the respective branches caused by gene flow. White and grey squares represent non-significant values or trios incompatible with the phylogenetic tree.

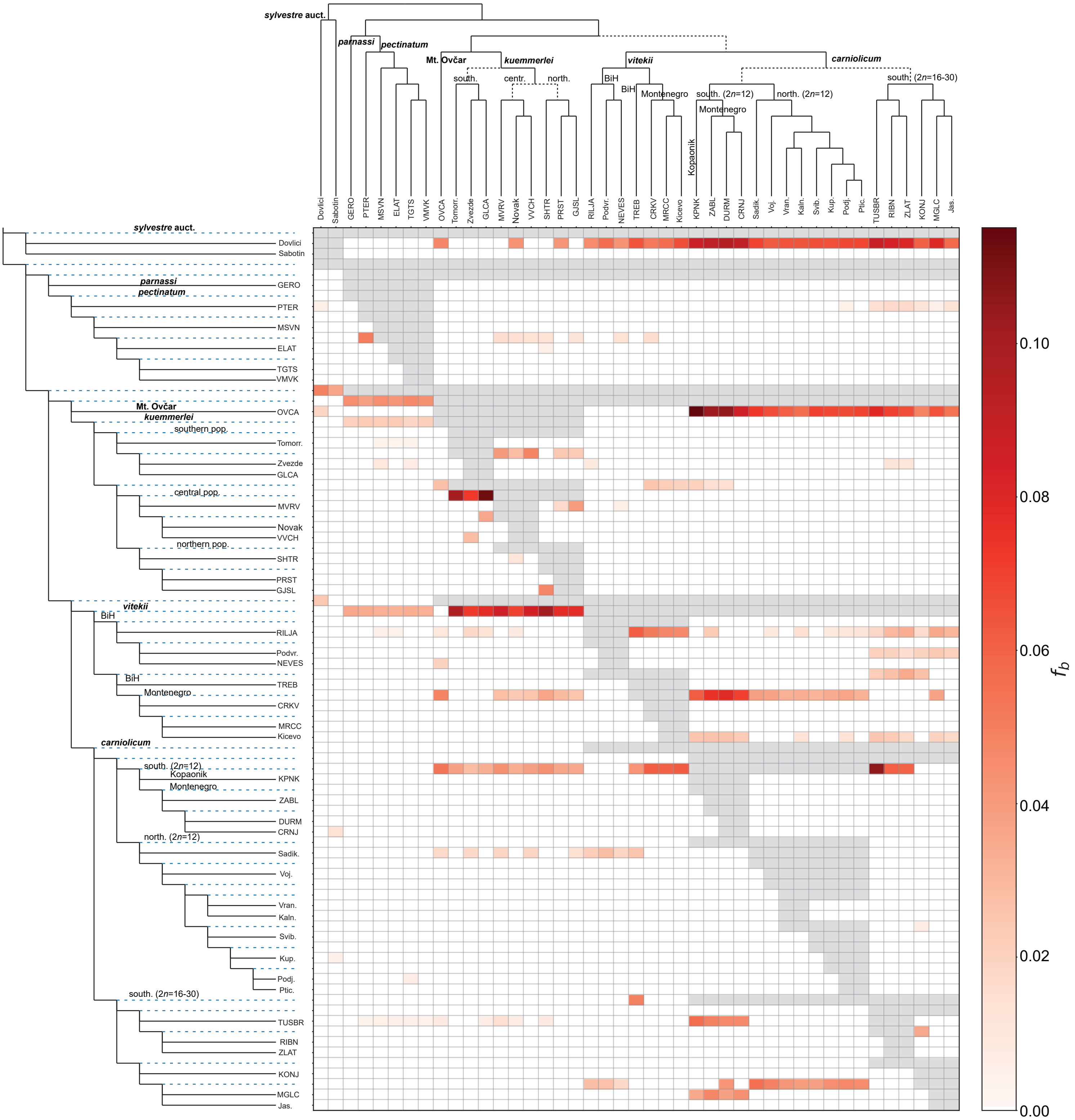

Figure S5 Morphometric analyses of Balkan species of the *Erysimum odoratum* complex, as shown in Fig. 5, here depicting also the total canonical structure coefficients, expressing the characters' contributions to the canonical axes.

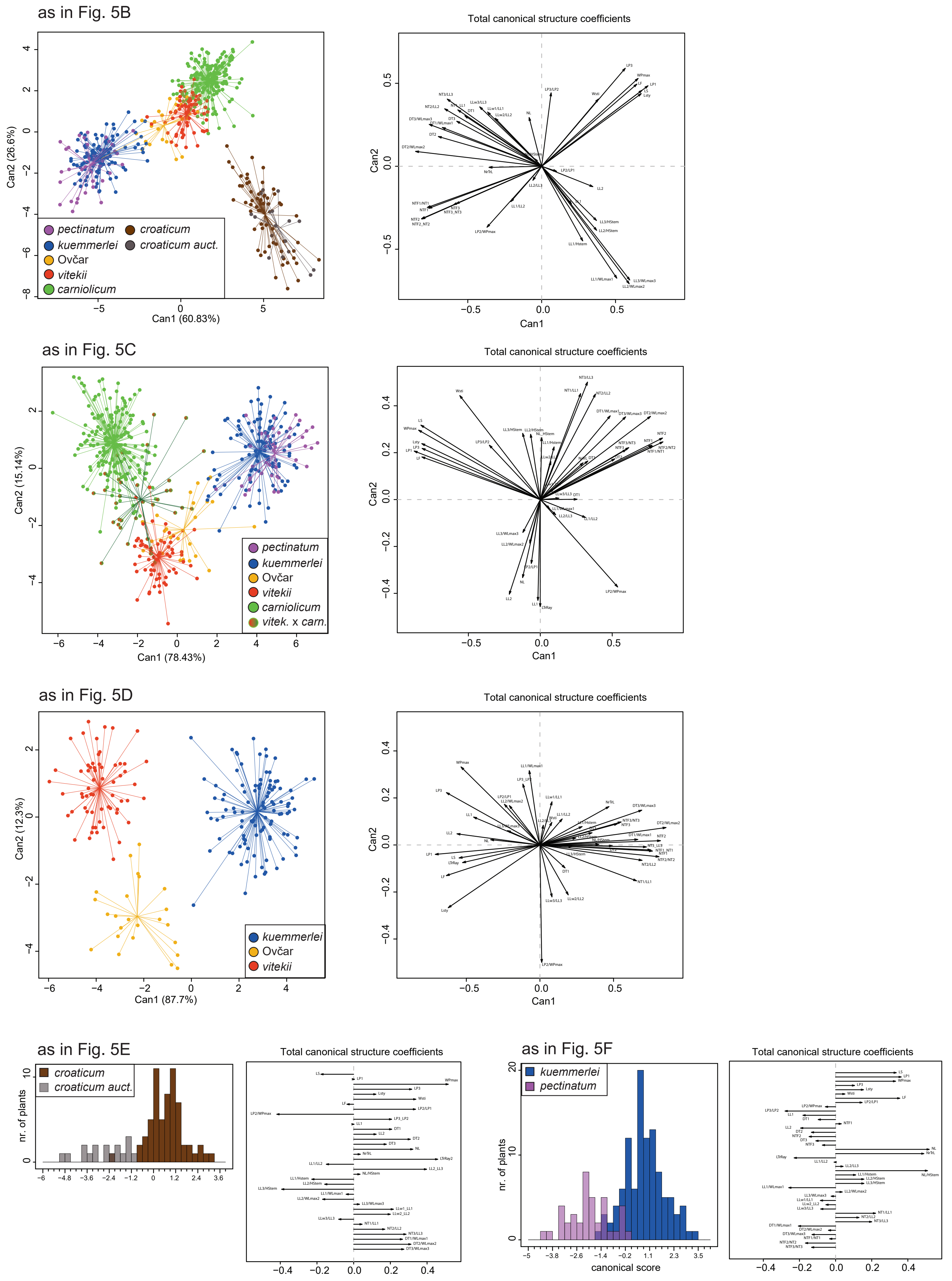

Figure S6 Canonical discriminant analyses depicting morphological differentiation between the here studied Balkan species *E. carniolicum* and *E. vitekii*, and the core species of the *E. odoratum* complex, *E. odoratum* and *E. witmannii*.

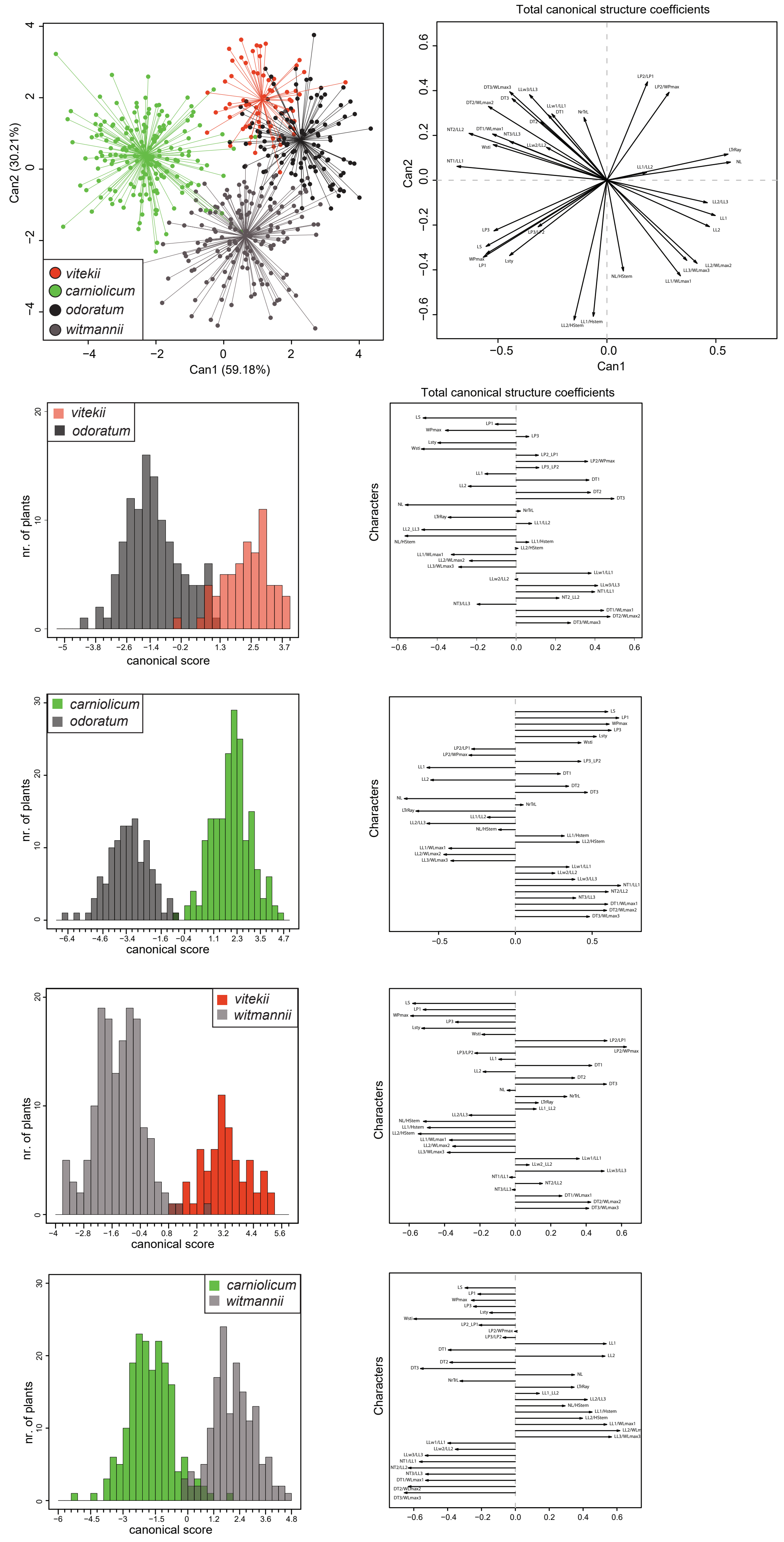

Supplement: Supplementary file 1 [file mcag229_supplementary_data.zip › SUPPLEMENTARY_MATERIAL.pdf]
