# Supplementary material for: Cryptic diversity, polyploidy and intraspecific divergence in Erysimum (Brassicaceae) species endemic to the western Balkans
Source: Ann Bot. 2026 Jul 30;138(5):1804–24. doi: 10.1093/aob/mcag229 (PMC13715005; doi:10.1093/aob/mcag229)
Supplement: Supplementary file 1 [file mcag229_supplementary_data.zip › TableS1_A3.pdf]

Table S1. List of the *Erysimum* populations analysed in present study. Asterisks in the column Populations code indicate type localities. The country abbreviations follow the international 2-letter ISO 3166-1 alpha-2 standard. Relative DNA content represents the sample/standard fluorescence ratio, using *Solanum lycopersicum* 'Stupické polní rané' (2C = 1.96 pg) as the primary standard, given in arbitrary units (mean ± standard deviation). Chromosome numbers in bold were determined in the present study (see Fig. S1), those with superscripts were published previously from the sampled localities. Flow-cytometric, RADseq and Hyb-Seq data taken from the previous study (Bačák *et al.* , 2026) are indicated by superscript [b] next to the population code and in the respective columns if applicable.

**Plant material information**							**Flow cytometry**		**Chromosome number**	**BioSample accession number**	**RADseq**	**Hyb-Seq**	**Morpho-metrics**
**Population code**	**Locality**	**Latit. N**	**Longit. E**	**Elevation (m a.s.l.)**	**Collected by**	**Collection date DD.M.YYYY**	**No. of analy-sed ind.**	**Relative DNA content - 2C**			**No. of analysed ind.**		
**Balkan species of the *Erysimum odoratum* species complex**													
E. carniolicum													
Kalnik	HR, Kalnik Mts, ruins of Veliki Kalnik castle	46.1331	16.4642	472	KS, JO	19.6.2022	4	0.463 ± 0.012	**2*n* =12**	SAMN55298468, SAMN55298469	2	1	
Vranilac	HR, Kalnik Mts, Mt. Vranilac	46.1313	16.4548	640	KS, JO	19.6.2022; 27.5.2023	4	0.446 ± 0.008		SAMN55298554, SAMN55298555	2	1	yes
Kupec	HR, Samobor Mts, N of the settlement Kupeć Dol	45.7363	15.6124	500	KS, JO	20.6.2022	4	0.439 ± 0.017		SAMN55298480, SAMN55298481	2	1	
Podjelak[b]	HR, Samobor Mts, Mt. Podjelak	45.7667	15.6247	632	KS, JO	20.6.2022	4	0.456 ± 0.034[b]	2*n* =12[b], **2*n* =12**	SAMN49725829, SAMN49725830	2[b]	1[b]	
Pticji	HR, Samobor Mts, Mt. Ptičji vrh	45.7678	15.6517	617	KS, JO	28.5.2023	4	0.452 ± 0.020		SAMN55298521, SAMN55298522	2	1	yes
Sadikovac	HR, Velebit Mts, Mt. Veliki Sadikovac	44.5052	15.1869	1,230	KS, JO	24.6.2022; 30.5.2023	8	0.441 ± 0.008		SAMN55298528, SAMN55298529	2	1	yes
Vojak_c	HR, Istria, Učka Mts, S slope of Mt. Vojak	45.2830	14.2018	1,340	KS, JO	22.6.2022; 29.5.2023	7	0.427 ± 0.009	2*n* =14[a]	SAMN55298552, SAMN55298553	2	1	yes
MGLC	BA, NP Sutjeska, W slope of Bukovi vrhovi peak	43.3167	18.7155	1,380	AK, MŠ; KS, JO, AB	21.6.2022	4	0.628 ± 0.029	**2*n* =16**	SAMN55298484, SAMN55298485	2	1	
Jasik[b]	BA, SE of Sarajevo, Jasik, settlement Bistrica	43.7762	18.5473	1,144	KS, JO; KS, JO, AB	1.6.2023	4	0.635 ± 0.013[b]	2*n* =16[b], **2*n* =16**	SAMN49725742, SAMN49725743	2[b]	1	yes
DURM	ME, Durmitor Mts, between Pašina voda and Sedlo	43.0972	19.0718	1,789	JZL, HM, EV	27.5.2024	4	0.229 ± 0.003	**2*n* =12**	SAMN55298448, SAMN55298449	2	1	yes
CRNJ	ME, Durmitor Mts, Crno Jezero	43.1449	19.0907	1,457	AK, MŠ; JZL, HM, EV	22.6.2022; 26.5.2024	4	0.231 ± 0.006	**2*n* =12**	SAMN55298442, SAMN55298443	2	1	yes
Zabljak	ME, Sinjajevina Mts, E of Žabljak, Tepačko Polje	43.1644	19.1630	1,350	VB, TK, FK	1.5.2024	5	0.228 ± 0.004		SAMN55298564, SAMN55298565	2	1	
KPNK	RS, Kopaonik Mts, E slopes of Velika gobelja peak	43.3194	20.8383	1,790	AK, MŠ; JZL, KM, MŠ, BŠ	24.6.2022; 9.7.2023	4	0.225 ± 0.003	**2*n* =12**	SAMN55298474-SAMN55298476	3	1	yes
KONJ	RS, Tara Mts, S of Konjska Reka	43.8938	19.4148	1,064	AK, MŠ	20.6.2022	4	0.630 ± 0.022		SAMN55298472, SAMN55298473	2	1	
TUSBR	RS, Zlatibor Mts, Tusto Brdo	43.5952	19.7104	1,002	JZL, HM, EV	25.5.2024	4	0.422 ± 0.005		SAMN55298546, SAMN55298547	2	1	
RIBN	RS, Zlatibor Mts, SW of the town of Zlatibor, towards Ribnicko jezero lake	43.6997	19.6718	954	JZL, HM, EV	24.5.2024	4	0.631 ± 0.006		SAMN55298524, SAMN55298525	2	1	yes
ZLAT	RS, Zlatibor Mts, S of the town of Zlatibor, SW slopes of Mt Kriva Breza	43.6926	19.6945	1,036	JZL, HM, EV	24.5.2024	4	0.669 ± 0.013	**2*n* =18, 2*n* =30**	SAMN55298566, SAMN55298567	2	1	yes
Svibno*[b]	SI, Posavsko Hribovje, Svibno, locus classicus of *E. carniolicum*	46.0664	15.0977	580	BŠ	20.6.2022	4	0.437 ± 0.007[b]	2*n* =12[b], 2*n* =14[a], **2*n* =12**	SAMN49725886, SAMN49725887	2[b]	1[b]	
E. croaticum													
GBre*[b]	HR, Splitsko-dalmatinska županija, between the villages of Gornja Brela and Zadvarje, locus classicus of *E. croaticum*	43.4008	16.9094	300	JZL, VC	3.5.2022	10	0.922 ± 0.022[b]	**2*n* =36, 38, 40**, 2*n* =42[a]	SAMN49725720, SAMN49725732	2[b]	1	yes
Omis	HR, Splitsko-dalmatinska županija, between the town of Omiš and Naklice	43.4511	16.6825	160	JZL, VC	2.5.2022	10	0.705 ± 0.021	**2*n* =30**	SAMN55298501, SAMN55298502	2	2	yes

Plant material information							Flow cytometry		Chromosome number	BioSample accession number	RADseq	Hyb-Seq	Morpho-metrics
**Population code**	**Locality**	**Latit. N**	**Longit. E**	**Elevation (m a.s.l.)**	**Collected by**	**Collection date DD.M.YYYY**	**No. of analy-sed ind.**	**Relative DNA content - 2C**			**No. of analysed ind.**		
Stipanici	BA, between the town of Tomislavgrad and Buško jezero, Stipanići	43.7064	17.1428	980	KS, JO	31.5.2023	7	0.711 ± 0.011	**2n =30**	SAMN55298534, SAMN55298535	2	1	yes
***E. croaticum* auct. (Kosovo)**													
GLSH	XK, Kosovo Polje Plain, NE of Mt. Golesh	42.5770	20.9866	870	AK	4.6.2024	5	0.669 ± 0.008		SAMN55298462, SAMN55315825	1	2	yes
PRIZ	XK, SE of the town of Prizren to the village of Reçan	42.2001	20.7644	535	AK, MŠ	25.6.2022	7	0.693 ± 0.018	**2n =36**, 2n =42[a]	SAMN55298517-SAMN55298519	3	2	
E. kuemmerlei													
SHTR*[b]	AL, Kukës region, between the villages of Shtrezë and Novosejë, locus classicus of *E. kuemmerlei*	41.9888	20.5556	1,240	AK, MŠ	25.6.2022	8	0.216 ± 0.007[b]	2n =12[b], **2n =12**	SAMN49725863, SAMN49725864, SAMN55298530	2[b]+1	1[b]	yes
Zvezde	AL, Korçë region, N of the village of Zvezdë	40.7487	20.8614	1,502	IT, PT	17.5.2024	4	0.404 ± 0.010		SAMN55298568, SAMN55298570	2	1	
Tomorrit	AL, Berat region, SE slopes of Mt. Tomorr	40.5988	20.1953	1,367	IT, PT	14.5.2024	4 / 1	0.403 ± 0.006 / 0.586		SAMN55298538, SAMN55298540-SAMN55298542	4	1	
MVRV[b]	MK, Mavrovo Mts, between the villages of Mavrovo and Galichnik	41.6413	20.6906	1,720	AK, MŠ	26.6.2022	4	0.211 ± 0.003[b]	2n =14[a], **2n =12**	SAMN49725802, SAMN49725803	2[b]	1	yes
GLCA	MK, Galičica Mts, E of the village of Trpejca, Baba	40.9540	20.8129	1,600	AK, MŠ; KS, SŠ	26.6.2022; 15.5.2022	8	0.396 ± 0.007	**2n =14**	SAMN55298453, SAMN55298458, SAMN55298459	3	1	yes
VVCH	MK, Jablanica Mts, NW of the village of Vevchani	41.2500	20.5816	1,280	AK	6.6.2024	4	0.210 ± 0.004		SAMN55298562, SAMN55298563	2	1	
NovakE1	MK, Strogovo Mts, W of Burinec	41.4031	20.5842	633	KS, SŠ; KS ,JO, AB	15.5.2022	4	0.213 ± 0.004		SAMN55298495, SAMN55298497	2	1	yes
PRST[b]	XK, Gollak Mts, E of the town of Pristina, NE of the peak of Maja me e larte e Butocit	42.6648	21.2371	1,080	AK	3.6.2024	4	0.210 ± 0.003[b]	**2n =12**	SAMN49725836, SAMN49725837	2[b]	1[b]	yes
GJSL	XK, Šar Planina Mts, between the villages of Gornje Selo and Prevallë	42.1832	20.9293	1,320	AK	4.6.2024	4	0.212 ± 0.003	2n =14[a], **2n =12**	SAMN55298456, SAMN55298457	2	1	yes
**population of ucertain taxonomic assignment, genetically close to *E. kuemmerlei***													
OVCA/Ovčar	RS, Ovčarsko-Kablarska klisura gorge, above the settlement of Dučalovići, SW of the Ovčar peak	43.8928	20.2109	874	AK, MŠ; JZL, KM, MŠ, BŠ; JZL, EV, HM	20.6.2022; 8.7.2023; 23.5.2024	8	0.223 ± 0.004	2n =14[a], **2n =12**	SAMN55298504-SAMN55298507	4	2	yes
E. pectinatum													
TGTS*[b]	GR, Taygetos Mts, Peloponnese, Messenia region, between the villages of Tripi and Artemisia, locus classicus of *E. pectinatum*	37.0778	22.2700	1,240	AK	2.5.2024	4	0.230 ± 0.003[b]	2n =12[b], **2n =12**	SAMN49725893, SAMN49725894	2[b]	1[b]	yes
MSVN	GR, Mainalos Mts, Peloponnese, Arkadía region, N of Mainalon ski center	37.6552	22.2662	1,600	AK	30.4.2024	3 / 2	0.439 ± 0.007 / 0.329 - 0.341	**2n =22**	SAMN55298490, SAMN55298491	2	1	
ELAT	GR, Mainalos Mts, Peloponnese, Arkadía region, W end of the village of Elati	37.6263	22.1465	1,170	AK	3.5.2024	3	0.227 ± 0.003	**2n =12**	SAMN55298450, SAMN55298451	2	1	
VMVK	GR, Parnonas Mts, Peloponnese, Arkadia region, SW of the village of Kastanitsa	37.2256	22.6133	1,530	AK	1.5.2024	4	0.230 ± 0.003	**2n =12**	SAMN55298550, SAMN55298551	2	1	
PTER[b]	GR, Panachaiko Mts, Peloponnese, Achaia region, NE of the village of Pteri	38.1570	22.0817	1,000	AK	4.5.2024	4	0.418 ± 0.011[b]	2n =14[a], 2n =16[b], **2n =16**	SAMN49725838, SAMN49725839	2[b]	1[b]	yes
E. vitekii													
CRKV*[b]	ME, W of the town of Kolašin, W of the Crkvine pass, locus classicus of *E. vitekii*	42.7963	19.4293	1,085	AK, MŠ; JZL, HM, EV	22.6.2022; 26.5.2024	4	0.219 ± 0.004[b]	2n =12[a], 2n =12[b], **2n =12**	SAMN49725717-SAMN49725719	3[b]	1[b]	yes

Plant material information							Flow cytometry		Chromosome number	BioSample accession number	RADseq	Hyb-Seq	Morpho-metrics
Population code	Locality	Latit. N	Longit. E	Elevation (m a.s.l.)	Collected by	Collection date DD.M.YYYY	No. of analy-sed ind.	Relative DNA content - 2C			No. of analysed ind.		
MRCC	ME, Morača river canyon, S of the Manastir Morača monastery	42.7562	19.3906	312	AK, MŠ	23.6.2022	4	0.211 ± 0.007	**2*n* =12**	SAMN55298488, SAMN55298489	2	1	yes
Kicevo	ME, between the towns of Kolašin and Šavnik, below Kičevo peak	42.8250	19.3674	533	JZL, HM, EV	26.5.2024	4	0.218 ± 0.000		SAMN55298470, SAMN55298471	2	1	
TREB	BA, SW of the town of Trebinje, between the settlements of Volujac and Ljubovo	42.6890	18.2698	290	JZL, VC	4.5.2022	3	0.432 ± 0.005		SAMN55298543- SAMN55298545	3	1	
Podvranić	BA, N of the town of Grude, NW of the village of Podvranić	43.4148	17.4134	630	KS, JO; KS, JO, AB	31.5.2023	4	0.405 ± 0.002	**2*n* =14**	SAMN55298513- SAMN55298515	3	1	yes
Neves[b]	BA, W of the town of Nevesinje, Grebak	43.2485	18.0944	1,090	JZL, HM, EV	27.5.2024	6	0.219 ± 0.002[b]	2*n* =12[b,] 2*n* =12[a], **2*n* =12**	SAMN49725808, SAMN49725809	2[b]	1[b]	yes
RILJA	BA, Bjelašnica Mts, E of the town of Nevesinje, settlement Rilja	43.2638	18.2808	931	JZL, HM, EV	27.5.2024	4	0.407 ± 0.004	**2*n* =14**	SAMN55298526, SAMN55298527	2	1	yes
**populations genetically admixed, intermediate between *E. vitekii* and *E. carniolicum* (suspected allopolyploids)**													
OBGL	ME, between the towns of Kolašin and Šavnik, below the Obla Glava peak	42.9184	19.2675	1,545	AK, MŠ; JZL, HM, EV	22.6.2022; 26.5.2024	4	0.477 ± 0.014	**2*n* =20, 22**	SAMN55298498- SAMN55298500	3	1	yes
VRMAC	ME, between the towns of Kolašin and Šavnik, below the Vrmac peak	42.8932	19.2756	1,269	JZL, HM, EV	26.5.2024	10	0.473 ± 0.011	**2*n* =22, 24**	SAMN55298557- SAMN55298559	3	1	yes
***Erysimum odoratum* species complex in a stricter sense (following Bačák et al. 2026)**													
E. odoratum													
Bacin[b]	HR, Papuk Mts, E of the village of Baćin Dol below Kula Gračanica hill	45.3098	17.4573	374	JZL, HM, EV	28.5.2024	4	0.471 ± 0.009[b]		SAMN49725662, SAMN49725663	2[b]		
Maliscak[b]	HR, Papuk Mts, S slope of Mališčak hill	45.4728	17.6375	544	KS, JO	22.6.2022	4	0.474 ± 0.006[b]	2*n* =22[b]	SAMN49725784, SAMN49725785, SAMN49725787	3[b]	1[b]	
CIUC[b]	RO, Munții Banatului Mts, Munții Almăjului, SW of the town of Orşova, above the village of Dubova, Ciucaru Mare	44.6156	22.2689	280	JZL, MŠ, BŠ, KM	11.6.2022; 11.7.2023	5	0.459 ± 0.010[b]	2*n* =22[b]	SAMN49725704, SAMN49725705, SAMN49725706	3[b]	1[b]	
RET[b]	RO, Munții Retezat Mts, Cheile Urzicarului gorge, below Vf. Piule peak	45.2817	22.9258	1,041	IT, PT, JS, RB	11.6.2024	4	0.477 ± 0.008[b]	2*n* =22[b]	SAMN49725849, SAMN49725850	2[b]	1[b]	
SCO[b]	RO, Munții Retezat Mts, Cheile Urzicarului gorge, below Vf. Piule Mt	45.2817	22.9258	1,041	IT, PR, JS, RB	11.6.2024	4	0.470 ± 0.007[b]		SAMN49725858, SAMN49725859	2[b]	1[b]	
GLAV[b]	RS, Fruška Gora Mts, Glavica hill	45.1807	19.8517	320	JZL, HM, EV	20.5.2024	4	0.936 ± 0.023[b]	2*n* ≈42**[b]	SAMN49725735, SAMN49725736	2[b]	1[b]	
VGrad[b]	RS, Fruška Gora Mts, Veliki Gradac hill	45.1653	19.7677	467	JZL, HM, EV	21.5.2024	4	1.104 ± 0.016[b]	2*n* ≈50**[b]	SAMN49725911		1[b]	
BREIT[b]	AT, Leithagebirge Mts, S of the village of Breitenbrunn	47.9369	16.7192	160	JZL, MŠ	17.5.2022	4	0.702 ± 0.015[b]	2*n* =32[b]	SAMN49725680	1[b]	1[b]	
Zukici[b]	BA, N of the town of Konjic, NE of the village of Zukići	43.7319	18.0073	671	KS, JO	2.6.2023	3	0.710 ± 0.009[b]		SAMN49725928, SAMN49725929	2[b]		
Busko[b]	BA, W of Buško Blato lake	43.6683	16.9749	754	KS, JO	31.5.2023	4	0.691 ± 0.012[b]	2*n* =32[a]	SAMN49725686, SAMN49725687	2[b]		
Vojak_o[b]	HR, Istria, Učka Mts, S slope of the Mt. Vojak	45.2830	14.2018	1,340	KS, JO	22.6.2022	4	0.699 ± 0.011[b]		SAMN49725914	1[b]		
V.Ucka[b]	HR, Istria, Učka Mts, S of the village of Vela Učka	45.2915	14.1948	1,030	KS, JO	21.6.2022	4	0.698 ± 0.021[b]	2*n* =32[a]	SAMN49725918	1[b]		
M.Ucka[b]	HR, Istria, Učka Mts, S of the village of Mala Učka	45.2721	14.1963	960	KS, JO	21.6.2022	4	0.706 ± 0.010[b]	2*n* =32[a]	SAMN49725801	1[b]		
Kavlar[b]	RS, Ovčarsko-Kablarska klisura gorge, Kavlar peak	43.9126	20.1910	788	JZL, HM, EV	24.5.2024	4	0.673 ± 0.007[b]	2*n* =32	SAMN49725746, SAMN49725747	2[b]	1[b]	
Bacevci[b]	RS, S of the town of Valjevo, Bačevci settlements	44.1553	19.8899	417	KS, SŠ	16.5.2022	4	0.707 ± 0.012[b]	2*n* =32[b]	SAMN49725659, SAMN49725660	2[b]		

Plant material information							Flow cytometry		Chromosome number	BioSample accession number	RADseq	Hyb-Seq	Morpho-metrics
Population code	Locality	Latit. N	Longit. E	Elevation (m a.s.l.)	Collected by	Collection date DD.M.YYYY	No. of analy-sed ind.	Relative DNA content - 2C			No. of analysed ind.		
E. witmannii													
GEMH[b]	SK, Slovenský kras Mts, educational trail N of the village of Gemerská Hôrka	48.5417	20.3778	340	JZL, EV	6.6.2023	4	0.233 ± 0.008[b]		SAMN49725733	1[b]	1[b]	
LES*[b]	SK, Pieniny Mts, Lesnica, the mouth of the Lesnický potok stream, locus classicus of *E. witmannii*	49.4144	20.4503	500	JZL	20.7.2021	4	0.232 ± 0.004[b]	$2n$ =14[b]	SAMN49725770, SAMN49725920	2[b]	1[b]	
NASZ[b]	HU, Cserhát Mts, N of the town of Vácz, W of the Naszály hill	47.8308	19.1428	480	JZL, VC	7.5.2022	4	0.234 ± 0.001[b]	$2n$ =14[a]	SAMN49725805, SAMN49725806	2[b]	1[b]	
TUN[b]	RO, Buila-Vânturarița, below Vf. Stogu peak	45.2746	24.1283	1,141	IT, PR, JS, RB	15.6.2024	4	0.229 ± 0.003[b]		SAMN49725901, SAMN49725902	2[b]		
ZARN[b]	RO, Piatra Craiului Mts, between the town of Zărneşti and the village of Magura	45.5478	25.2961	846	IT, PR, JS, RB	12.7.2022; 12.6.2023	5	0.436 ± 0.016[b]	$2n$ =16[b]	SAMN49725924, SAMN49725925	2[b]	1[b]	
SUGA[b]	RO, Munţii Haşmaş Mts, Cheile Şugăului gorge	46.8248	25.8509	674	IT, PR, JS, RB, IDB	11.7.2022; 10.6.2023	2 / 1	0.232 - 0.235 / 0.338[b]	$2n$ =14[b]	SAMN49725872, SAMN49725874	2[b]	1[b]	
other included species													
E. asperulum													
PTSK	GR, Peloponnese, Achaia region, NW of the village of Petsaki	38.1029	22.0296	920	AK	3.5.2024	2	0.428 - 0.428		SAMN55298523	1		
MTCH	GR, Peloponnese, Achaia region, W of the village of Manesi	38.0150	21.9481	820	AK	3.5.2024	2	0.425 - 0.427		SAMN55298492	1		
KRKL	GR, Vardousia Mts, Central Greece, Phocis region, NW of the village Krokilio	38.5514	22.0544	990	AK	4.5.2024	2	0.417 - 0.421		SAMN55298478	1		
E. bulgaricum													
BALG	RO, Constanța region, NE of the village of Băltăgeşti, Vârful Allah Bair hill	44.4976	28.2194	200	SŠ, DRL	2.5.2024	2	0.220 - 0.233		SAMN55298435	1		
E. calycinum													
Drite	AL, Dibër region, Dritë, NW of the town of Burrel	41.6335	19.9790	192	KS, JO, AB	19.8.2024	3	0.427 ± 0.005		SAMN55298446, SAMN55298447	2		
LPSH[b]	AL, Prokletije Mts, Shkodër regions, E of the village of Gropat e Selcës	42.5404	19.6968	1,200	AK	8.6.2024	2	0.429 - 0.433[b]		SAMN49725775	1[b]		
IZVR[b]	MK, W of the town of Kičevo, SW of the village of Izvor, near the Treska river spring	41.4774	20.8148	770	AK	6.6.2024	2	0.407 - 0.409[b]		SAMN49725739	1[b]	1	
Tajmiste	MK, NW of the twon of Kičevo, crossroad to the village of Tajmishte	41.6480	20.8848	889	KS, JO, AB	22.8.2024	4	0.425 ± 0.004		SAMN55298537	1		
Botun	MK, between the towns of Ohrid and Kičevo, S of the village of Botun	41.2788	20.7836	755	KS, JO, AB	22.8.2024	4	0.427 ± 0.003		SAMN55298437, SAMN55298438	2		
NovakE2	MK, Strogovo Mts, between Burinec and Dolgash	41.4031	20.5842	633	KS, SŠ; KS, JO, AB	15.5.2022	1	0.431		SAMN55298496	1		
E. cephalonicum													
KSTR	GR, Peloponnese, Achaia region, N of the village of Kastria	37.9692	22.1423	960	AK	3.5.2024	2	0.441 - 0.456		SAMN55298479	1		
MTMA	GR, Mainalos Mts, Peloponnese, Arkadía region, S of Mainalon ski center	37.6458	22.2671	1,550	AK	30.4.2024	2	0.437 - 0.465		SAMN55298493	1	1	
VMPS	GR, Parnonas Mts, Peloponnese, Arkadia region, SW of the village Kastanitsa	37.2368	22.6109	1,480	AK	1.5.2024	1	0.471		SAMN55298549	1		
PRIL	GR, Vardousia Mts, NWW of the village of Prosilio, Mt. Prophitis Ilias	38.6055	22.2694	2,257	SŠ, DRL	23.6.2024	2	0.703 - 0.714		SAMN55298516	1		
MOUS	GR, Vardousia Mts, W of the village of Athanasios Diakos, Mousounitsiotiki Dhiasela pass	38.7027	22.0983	1,994	SŠ, DRL	22.6.2024	2	0.468 - 0.481		SAMN55298487	1		
E. comatum													
ZvezdeP11	AL, Korçë region, N of the village of Zvezdë	40.7527	20.8541	1,658	IT, PT	17.5.2024	2	0.214 - 0.224		SAMN55298569	1		
CAKR[b]	ME, Plav region, NE of the Čakor pass	42.6802	20.0127	1,935	AK, MŠ	23.6.2022	3 / 1	0.226 ± 0.005 / 0.331[b]		SAMN49725689	1[b]	1	
GLCC	MK, Galičica Mts, E of the village of Trpejca, Baba	40.9540	20.8129	1,600	AK, MŠ	26.6.2022	4	0.222 ± 0.006		SAMN55298454, SAMN55298460	2		
MarkoviKuli[b]	MK, SE of Skopje, S of the vilage of Badar, Markovi Kuli castle ruins above Pčinja river	41.8382	21.6988	276	KS, SŠ	11.5.2022	2	0.207 - 0.209[b]		SAMN49725792	1[b]		
CHEIA[b]	RO, Buila-Vânturarița, above Cheile Răului Cheia gorge	45.2635	24.1205	1,450	IT, PT, JS, RB	15.6.2024	2	0.232 - 0.238[b]		SAMN49725696	1[b]		
BUILA[b]	RO, Buila-Vânturarița, below the Vânturarița peak	45.2449	24.1024	1,670	IT, JS, RB	18.7.2022	2	0.224 - 0.227[b]		SAMN49725685	1[b]	1	

Plant material information							Flow cytometry		Chromosome number	BioSample accession number	RADseq	Hyb-Seq	Morpho-metrics
Population code	Locality	Latit. N	Longit. E	Elevation (m a.s.l.)	Collected by	Collection date DD.M.YYYY	No. of analy-sed ind.	Relative DNA content - 2C			No. of analysed ind.		
HER	RO, E of Băile Herculane spa	44.8661	22.4166	290	IT, PT, JS, RB	10.6.2024	2	0.461 - 0.473		SAMN55298466	1		
E. cf cuspidatum													
BAND	BG, Pirin, Bansko, Banderitsa chalet	41.7682	23.4264	1,800	JZL, MŠ, BŠ, KM; AK	9.6.2022; 26.6.2024	2	0.418 - 0.427		SAMN55298436	1		
E. cuspidatum													
GLCH	MK, Galičica Mts, E of the village of Trpejca	40.9653	20.8169	1,486	AK, MŠ	26.6.2022	2	0.436 - 0.443		SAMN55298461	1		
SPSH	MK, S of Skopje, Sopishte	41.9528	21.4222	530	AK	5.6.2024	2	0.439 - 0.444		SAMN55298533	1		
PEC[b]	RO, Pecinişca S of the Băile Herculane spa	44.8624	22.3910	130	IT, PR, JS, RB	10.6.2024	4	0.429 ± 0.004[b]		SAMN49725817, SAMN49725818	2[b]		
SVIcus	RO, Portiļe de Fier, between the villages of Cozla and Sviniţa, above the Danube river	44.6104	22.0061	78	IT, PR, JS, RB	9.6.2024	1	0.442		SAMN49725931	1	1	
cusBRUS[b]	RS, Kopaonik Mts, N of the town of Brus	43.3947	21.0337	500	AK	3.6.2024	2	0.425 - 0.428[b]		SAMN49725684	1[b]		
Hadic	RS, NE of the city of Niš, between the villages of Vrelo and Grbavce	43.3921	22.0513	530	JZL, HM, EV	21.5.2024	2	0.443 - 0.443		SAMN55298465	1		
KONJ[b]	RS, Tara Mts, S of Konjska Reka	43.8938	19.4148	1,064	AK, MŠ	20.6.2022	2	0.423 - 0.442[b]		SAMN49725757	1[b]	1[b]	
OzrenCusp	RS, Ozren Mts, E of Brodarevo	43.2346	19.7461	674	JZL, HM, EV	25.5.2024	1	0.437		SAMN55298509	1		
E. diffusum s.l. (E. crassistylum)													
PTL[b]	MK, Nidže Mts, Pelagoniski region, S of the village of Beshishte	41.1289	21.7894	981	KS, SŠ	14.5.2022	1	0.213[b]		SAMN49725654	1[b]		
RDU[b]	MK, Skopski region, between the villages of Rashche and Radusha	42.0541	21.2553	325	KS, SŠ	10.5.2022	2	0.207 - 0.214[b]		SAMN49725653	1[b]	1	
E. diffusum s.l. (E. cf pirinicum)													
ORLK	BG, Pirin Mts, NW of Popovi Livadi, Mt. Oreljak	41.57038	23.613135	2,080	AK	27.5.2024	2	1.652 - 1.668		SAMN55298503	1		
E. diffusum s.l. (E. cf welchevii)													
Nise	RS, Svrljig Mts, Niševac gorge	43.47169	22.096722	341	JZL, HM, EV	22.5.2024	2	0.644 - 0.658		SAMN55298494	1		
Vrelo	RS, NE of the city of Niš, N of the village of Vrelo	43.3814	22.0460	390	JZL, HM, EV	21.5.2024	2	0.661 - 0.668		SAMN55298556	1		
E. diffusum s.l.													
SVI[b]	RO, Portiļe de Fier, between the villages of Cozla and Sviniţa, above the Danube river	44.6104	22.0061	78	IT, PR, JS, RB	9.6.2024	9	0.681 ± 0.020[b]		SAMN49725883-SAMN49725885	3[b]		
COR[b]	RO, Munţii Banatului Mts, Portiļe de Fier, S of the village Coronini, above the Danube river	44.6686	21.6899	83	IT, PR, JS, RB	9.6.2024	4	0.674 ± 0.008[b]		SAMN49725709-SAMN49725712	4[b]		
BREH[b]	SK, Východoslovenská nížina Plain, Brehov, Veľký vrch hill	48.4931	21.8064	141	KS, SŠ	6.10.2022	5	0.415 ± 0.004[b]		SAMN49725679	1[b]		
LEV[b]	SK, Záhorská nížina Plain, W of the village of Malé Leváre, near Rudavské jazero lake	48.5053	16.9528	150	JZL	14.7.2021	4	0.425 ± 0.003[b]		SAMN49725771	1[b]	1	
E. diffusum s.l. (E. cf canum)													
DANK	SK, Podunajská nížina Plain, NW of Nána, between the vineyards and Vrch Dank hill	47.8208	18.6459	220	AK	25.4.2024	2	0.848 - 0.857		SAMN55298444	1		
GAB	SK, Podunajská nížina Plain, Gabčíkovo, between 'café kompa' and port	47.8628	17.5331	120	JZL	14.6.2021	3	1.326 ± 0.128		SAMN55298452	1		
CERN	RO, Munţii Măcinului Mts, NW of the village of Cerna, Muchia lungă hill	45.10888	28.250506	140	SŠ, DRL	3.5.2024	2	1.097 - 1.121		SAMN55298441	1		
E. graecum													
KRIA	GR, Peloponnese, Achaia region, NE of Klitoria	37.9172	22.1435	550	AK	3.5.2024	2	0.418 - 0.423		SAMN55298477	1		
SLLS	GR, Parnonas Mts, Peloponnese, Laconia, between the villages of Sellasia and Vasaras	37.1850	22.4691	570	AK	2.5.2024	2	0.420 - 0.420		SAMN55298532	1		
SKOT	GR, Trachi Mts, Peloponnese, Argolida, E of the village Skoteini	37.8003	22.4522	700	AK	30.4.2024	2	0.419 - 0.428		SAMN55298531	1		
ACHR	GR, Parnitha Mts, East Attica, Athens-Acharnes	38.1169	23.7232	350	AK	30.4.2024	2	0.418 - 0.422		SAMN55298433	1	1	
E. hayekii													
PHTS	GR, Kalymnos, NE of the town of Chorio, Mt. Prophitis Ilias	36.9796	26.9730	645	SŠ, MŠ	24.3.2024	2	0.215 - 0.218		SAMN55298512	1		
E. horizontale													
LESB	GR, Lesbos, SW of Agiasos, Mt. Olympos	39.0711	26.3538	846	SŠ, MŠ	27.3.2024	2	0.415 - 0.425		SAMN55298482	1		
E. cheiranthoides													
Balaze[b]	SK, Nízke Tatry Mts, Baláže, Marková dolina valley	48.8280	19.1990	654	KS	23.8.2022	4	0.231 ± 0.004[b]		SAMN49725665	1[b]		

Plant material information							Flow cytometry		Chromosome number	BioSample accession number	RADseq	Hyb-Seq	Morpho-metrics
Population code	Locality	Latit. N	Longit. E	Elevation (m a.s.l.)	Collected by	Collection date DD.M.YYYY	No. of analy-sed ind.	Relative DNA content - 2C			No. of analysed ind.		
GAJ[b]	SK, Záhorská nížina Plain, W of the village of Gajary	48.4676	16.8747	140	JZL	14.7.2021	2	0.216 - 0.219[b]		SAMN49725731	1[b]		
E. cf janchenii													
Ozren	RS, Ozren Mts, E of Brodarevo	43.2346	19.7461	674	JZL, HM, EV	25.5.2024	2	0.694 - 0.721		SAMN55298508, SAMN55315826	1	2	
E. linariifolium													
TomorritT2	AL, Berat region, SE slopes of Mt. Tomorr	40.6011	20.1958	1,344	IT, PT	14.5.2024	1	0.218		SAMN55298539	1		
Gacko[b]	BA, NE of the town of Gacko	43.2051	18.5680	1,099	JZL, HM, EV	27.5.2024	2	0.236 - 0.241[b]		SAMN49725730	1[b]		
STOZ	ME, Durmitor Mts, below the Stožina peak	43.0927	19.0859	1,707	AK, MŠ	22.6.2022	4	0.223 ± 0.003		SAMN55298536	1		
GORPO	ME, Bjelasica Mts, S of the town of Mojkovac, S of the village of Gornje Polje	42.9285	19.5759	857	JZL, HM, EV	25.5.2024	2	0.228 - 0.232		SAMN55298463	1		
TUSI[b]	ME, Sinjajevina Mts, E of the town of Šavnik, Tušinja	42.9521	19.1958	1,100	JZL, HM, EV	26.5.2024	2	0.236 - 0.241[b]		SAMN49725905	1[b]		
linKavlar[b]	RS, Ovčarsko-Kablarska klisura gorge, Kavlar peak	43.9126	20.1848	867	JZL, HM, EV	24.5.2024	2	0.226 - 0.240[b]		SAMN49725930	1[b]	1[b]	
DUCA[b]	RS, Ovčarsko-Kablarska klisura gorge, above the settlement of Ducalovici, SW of the Ovčar peak	43.8912	20.2109	840	AK, MŠ	20.6.2022	4	0.226 ± 0.001[b]		SAMN49725728	1[b]	1[b]	
E. microstylum													
Alona[b]	GR, Dytikí Makedonía region, W of town the of Florina, N of the village of Alona	40.7867	21.3026	1,268	KS, SŠ	13.5.2022	2	0.204 - 0.205[b]		SAMN49725656	1[b]	1[b]	
E. parnassi													
Gero	GR, Parnassós Mts, N of the town of Arachova, NW of Mt. Gerontovrachos summit	38.5369	22.5912	2,136	SŠ, DRL	21.6.2024	2	0.445 - 0.459		SAMN55298455, SAMN55315824	1	2	
E. pseudocuspidatum													
MNLN	GR, Mainalos Mts, Peloponnese, Arkadía region, Mainalos ski center	37.6532	22.2663	1,570	AK	30.4.2024	2	0.223 - 0.225		SAMN55298486	1		
Vrondero	GR, Dytikí Makedonía region, SE of the village of Vrondero, W of Mikri Prespa lake	40.7189	21.0347	975	KS, SŠ	13.5.2022	4	0.228 ± 0.003		SAMN55298560, SAMN55298561	2	1	
E. pseudorhaeticum													
642CAT2	IT, Appennino Umbro Marchigiano Mts, N of the village of Chiaserna	43.4601	12.6680	819	KS, SŠ	2.5.2022	2	0.250 - 0.256		SAMN55298432	1		
639CLB2	IT, Monte Cetona, near Casa al Berna	42.9515	11.8528	629	KS, SŠ	1.5.2022	2	0.258 - 0.259		SAMN55298430	1	1	
640SIV2	IT, Monte Cetona, saddle N of Sopra il Varco	42.9474	11.8695	863	KS, SŠ	1.5.2022	2	0.262 - 0.273		SAMN55298431	1		
Vent	IT, Monti Sibillini Mts, SE of the town of Norcia, Strada provinciale di Castelluccio	42.7704	13.1520	1,200	KM, MŠ	22.5.2023	2	0.243 - 0.246		SAMN55298548	1		
E. pusillum													
PROF	GR, Taygetos Mts, Peloponnese, Laconia, NE of Profitis Ilias peak	36.9589	22.3879	1,210	AK	2.5.2024	2	0.225 - 0.228		SAMN55298520	1	1	
E. rhaeticum													
PFDS	AT, Tirol, Oberinntal, Pfunds - Stuben	46.9712	10.5392	1,011	MŠ	5.7.2022	4	0.986 ± 0.030		SAMN55298510, SAMN55298511	2		
CDLA	FR, Hautes-Alpes depart., NE of Col du Lautaret Pass	45.0429	6.4174	1,980	AK	25.6.2023	2	0.967 - 0.985		SAMN55298440	1		
GRNG	CH, Valais canton, Zermatt, SW of the Gornergrat station	45.9803	7.7774	2,800	AK	29.6.2023	2	1.006 - 1.013		SAMN55298464	1		
LLDN	CH, Valais canton, NE of the town of Visp, N of the train station Lalden	46.3014	7.9097	820	AK	30.6.2023	2	1.126 - 1.173		SAMN55298483	1		
BRUS	CH, Graubünden canton, Val Poschiavo valley, N of Brusio	46.2651	10.1251	877	KM, MŠ	20.5.2023	2	0.929 - 0.946		SAMN55298439	1		
JOLI	CH, Valais canton, NW of the town of Visp, N of the village of Niedergesteln	46.3176	7.7889	970	AK	30.6.2023	2	1.181 - 1.212		SAMN55298467	1		
E. smyrnaeum													
AGDI	GR, Kos, Mt. Dikaios, S of Agios Dimitrios chapel	36.8372	27.2247	840	SŠ, MŠ	22.3.2024	2	0.222 - 0.237		SAMN55298434	1		
DKES	GR, Kos, Mt. Dikaios, S of the villages of Asfendiou and Zia	36.8312	27.1997	721	SŠ, MŠ	22.3.2024	2	0.217 - 0.229		SAMN55298445	1		
***E. sylvestre* auct.**													
Dovlici[b]	BA, SE of Sarajevo, Mt. Trebević, N of Dovlići	43.8326	18.4703	1,147	KS, JO	1.6.2023	4	0.245 ± 0.004[b]		SAMN49725726	1[b]	1[b]	
Sab/Sabotin[b]	SI, Trnovski Gozd Plateau, N of the town of Gorizia/Nova Gorica, below the peak of Mt. Sabotin	45.9900	13.6320	575	MŠ, KM	15.5.2022	2	0.249 - 0.253[b]		SAMN49725855	1[b]	1[b]	
***Malcolmia graeca* (outgroup)**													

Plant material information							Flow cytometry		Chromosome number	BioSample accession number	RADseq	Hyb-Seq	Morpho-metrics
Population code	Locality	Latit. N	Longit. E	Elevation (m a.s.l.)	Collected by	Collection date DD.M.YYYY	No. of analy-sed ind.	Relative DNA content - 2C			No. of analysed ind.		
Malcolmia_II[b]	GR, Mainalos Mts, Peloponnese, Arkadía, N of the village of Dimitsana	37.6008	22.0388	950	AK	2.5.2025	3	0.264 ± 0.010[b]		SAMN49725783	1[b]	1[b]	
Malcolmia_I[b]	GR, Taygetos Mts, Peloponnese, Laconia, W of the village of Tripi	37.0919	22.3244	680	AK	2.5.2024	3	0.273 ± 0.010[b]		SAMN49725782	1[b]	1[b]	

References

a, Polatschek A. 2013. Revision der Gattung *Erysimum* (Cruciferae): Teil 5: Nord-, West-, Zentraleuropa, Rumänien und westliche Balkan-Halbinsel bis Albanien. Annalen des Naturhistorischen Museums in Wien, 115: 75-218.

b, Bačák R, et al. 2026. Understanding early stages of speciation: allopatric divergence, introgression and chromosomal dynamics in the *Erysimum odoratum* species complex. Molecular Phylogenetics and Evolution 214: 108481. https://doi.org/10.1016/j.ympev.2025.108481

Collectors

AB, Andrej Belák
AK, Adam Kantor
BŠ, Barbora Šingliarová
DRL, Dominik Roman Letz
EV, Edina Víziová (Kminiaková)
FK, Filip Kolář
HM, Hana Majerová
IDB, István-Dávid Becze
IT, Ingrid Turisová
JO, Jolana Olšavská
JS, Janka Smatanová
JZL, Judita Zozomová-Lihová
KM, Karol Marhold
KS, Katarína Skokanová
MŠ, Marek Šlenker
PT, Peter Turis
RB, Richard Bačák
SŠ, Stanislav Španiel
TK, Tereza Koberová
VB, Vít Bureš
VC, Veronika Cetlová
