# Supplementary material for: Cryptic diversity, polyploidy and intraspecific divergence in Erysimum (Brassicaceae) species endemic to the western Balkans
Source: Ann Bot. 2026 Jul 30;138(5):1804–24. doi: 10.1093/aob/mcag229 (PMC13715005; doi:10.1093/aob/mcag229)
Supplement: Supplementary file 1 [file mcag229_supplementary_data.zip › TableS5_A3.pdf]

Table S5. Descriptive statistics for quantitative morphological characters measured in the studied *Erysimum* species based on field-collected population samples (see Table S1). Characters referred to in the text and identified as diagnostic are highlighted in bold. Variation in trichome type is presented in Table S4. The delimitation of *E. odoratum* and *E. witmannii* follows Bačák et al. 2026, and the statistics is based on all populations previously analyzed in that study. HR, BA, XK refer to Croatia, Bosnia and Herzegovina, and Kosovo.

**Characters/species**	**Character abbreviation**	***E. carniolicum***	***E. vitekii***	***E. carniolicum* x *E. vitekii***	***E. kuemmerlei***	***E. pectinatum***	**population from Mt. Ovčar**	***E. croaticum* (HR, BA)**	***E. croaticum* auct. (XK)**	***E. odoratum***	***E. witmannii***
		(5%-)MEAN(-95%)	(5%-)MEAN(-95%)	(5%-)MEAN(-95%)	(5%-)MEAN(-95%)	(5%-)MEAN(-95%)	(5%-)MEAN(-95%)	(5%-)MEAN(-95%)	(5%-)MEAN(-95%)	(5%-)MEAN(-95%)	(5%-)MEAN(-95%)
		Nr. of ind: 193	Nr. of ind: 66	Nr. of ind: 35	Nr. of ind: 112	Nr. of ind: 40	Nr. of ind: 28	Nr. of ind: 62	Nr. of ind: 17	Nr. of ind: 515	Nr. of ind: 414
**Length of sepal (mm)**	LS	**(7.94-)10.53(-12.99)**	**(6.65-)7.85(-9.05)**	(7.81-)9.47(-11.31)	**(5.19-)6.77(-8.35)**	**(5.3-)6.22(-7.45)**	(6.72-)7.72(-8.56)	(7.85-)9.17(-10.86)	(7.79-)9.53(-11.27)	(7.1-)8.75(-10.53)	(7.3-)9.63(-12.04)
**Length of petal (including claw and blade) (mm)**	LP1	**(15.83-)20.5(-24.88)**	**(13.52-)15.97(-18.59)**	(15.4-)18.97(-23.38)	**(9.35-)12.46(-15.75)**	**(9.35-)11.3(-12.95)**	(13.42-)15.37(-17.38)	(14.91-)17.92(-21.05)	(14.87-)17.91(-20.76)	(13.14-)16.51(-20.03)	(13.91-)18.99(-24.75)
**Lenght of petal blade (mm)**	LP2	**(6.02-)8.07(-10.13)**	**(5.31-)6.69(-8.2)**	(5.66-)7.61(-9.4)	**(3.72-)4.91(-6.73)**	**(3.55-)4.33(-5.18)**	(5.23-)6.08(-7.27)	(5.68-)7.33(-8.71)	(5.83-)6.9(-7.85)	(5-)6.64(-8.41)	(5.08-)7.15(-9.65)
**Max. width of petal blade (mm)**	WPmax	**(5.4-)7.76(-10.1)**	**(4.2-)5.31(-6.58)**	(5.33-)6.67(-8.62)	**(2.47-)3.83(-5.99)**	**(2.59-)3.23(-4.06)**	(2.69-)4.01(-4.89)	(4.93-)6.28(-7.65)	(3.89-)5.27(-6.34)	(4.4-)6.03(-7.68)	(4.7-)6.88(-9.54)
Length from the widest point of the petal blade to its tip (mm)	LP3	(2.25-)3.13(-4.07)	(1.81-)2.33(-2.87)	(2.1-)2.68(-3.54)	(1.09-)1.63(-2.45)	(1.18-)1.55(-2.14)	(1.42-)1.89(-2.44)	(1.71-)2.36(-3.09)	(1.36-)2.08(-2.45)	(1.49-)2.24(-3.03)	(1.72-)2.7(-3.91)
**Length of style (mm)**	Lsty	**(9.94-)13.75(-17.25)**	**(8.11-)10.01(-11.92)**	(10.36-)12.83(-16.53)	**(5.81-)7.91(-10.39)**	**(6.19-)7.55(-8.67)**	(7.32-)10.68(-13.22)	(8.54-)12.11(-15.58)	(9.91-)11.64(-13.94)	(8.49-)11.3(-14.81)	(8.76-)12.69(-17.22)
Width of stigma (mm)	Wsti	(0.83-)1.38(-2.11)	(0.64-)0.87(-1.17)	(0.66-)0.95(-1.31)	(0.59-)0.89(-1.22)	(0.65-)0.87(-1.12)	(0.54-)0.83(-1.12)	(0.83-)1.11(-1.46)	(0.77-)0.98(-1.15)	(0.68-)1.04(-1.59)	(0.55-)0.94(-1.38)
**Length of longer filaments**	LF	**(9.13-)12.26(-15.45)**	**(7.78-)9.55(-11.16)**	(8.91-)11.54(-14.45)	**(5.04-)7.53(-9.44)**	**(4.5-)6.63(-8.31)**	(7.8-)9.58(-11.41)	(8.53-)10.37(-12.69)	(8.5-)10.48(-12.85)	(7.52-)10.28(-12.83)	(8.2-)11.76(-15.67)
Ratio Length of petal blade/Length of petal (including claw and blade)	LP2/LP1	(0.35-)0.39(-0.44)	(0.37-)0.42(-0.48)	(0.35-)0.4(-0.46)	(0.33-)0.4(-0.49)	(0.33-)0.38(-0.44)	(0.36-)0.4(-0.46)	(0.36-)0.41(-0.46)	(0.34-)0.39(-0.43)	(0.34-)0.4(-0.46)	(0.32-)0.38(-0.43)
**Ratio Length/Max. width of petal blade**	LP2/WPmax	**(0.87-)1.06(-1.32)**	(1.02-)1.27(-1.58)	(0.97-)1.16(-1.44)	(1-)1.33(-1.73)	(1.15-)1.35(-1.63)	**(1.27-)1.56(-2.13)**	(0.96-)1.18(-1.49)	(1.08-)1.33(-1.76)	(0.9-)1.12(-1.36)	(0.85-)1.05(-1.3)
Ratio Length from the widest point of the petal blade to its tip/Length of petal blade	LP3/LP2	(0.31-)0.39(-0.48)	(0.28-)0.35(-0.46)	(0.29-)0.35(-0.42)	(0.25-)0.33(-0.4)	(0.25-)0.36(-0.48)	(0.24-)0.31(-0.38)	(0.24-)0.32(-0.4)	(0.2-)0.3(-0.38)	(0.24-)0.34(-0.43)	(0.27-)0.38(-0.49)
**Length of lower stem leaf (including petiole if present) (mm)**	LL1	(16-)30.88(-48)	(28.05-)43.69(-72.75)	(20.7-)40.6(-70.6)	(16-)30.41(-50)	(17-)34.03(-54.05)	(19.75-)36.63(-56.7)	(28.05-)42.16(-58)	(26.6-)42.12(-64)	(27-)47.29(-78)	(24-)46.06(-83.05)
Max. width of lower stem leaf (mm)	WLmax1	(3-)6.24(-10.43)	(4.5-)8.07(-12)	(4-)7.43(-12)	(3-)6.02(-10)	(3-)6(-9.03)	(4.5-)8.48(-14.7)	(2-)3.45(-5)	(1.8-)3.41(-5.6)	(4-)7.8(-14)	(3-)6.5(-12.5)
Max. depth of teeth on lower stem leaf margin (mm)	DT1	(1-)2.13(-4.43)	(1-)2.25(-4.48)	(0.85-)2.44(-5)	(1-)2.7(-4.5)	(1.48-)2.88(-4.5)	(1-)2.63(-4.85)	**(0-)0.57(-1.5)**	**(0-)0.38(-1)**	(0.5-)1.53(-3.5)	(0.2-)1.46(-3.5)
Number of the teeth on one side of the lower stem leaf margin	NT1	(4-)5.51(-8)	(3-)4.98(-7)	(2.7-)5.37(-7)	(5-)6.82(-9.75)	(5-)6.48(-9)	(4-)6.21(-9)	(0-)2.21(-4)	(0-)2.06(-4)	(2-)4.21(-6)	(2-)4.9(-7.35)
Lenght from the widest part of the lower stem leaf to its tip (mm)	LLw1	(5-)12.22(-21.85)	(9-)17.27(-30.9)	(6.7-)15.49(-31.1)	(5.25-)12.42(-24)	(7-)14.28(-26.1)	(4.45-)12.58(-21.55)	(5-)10.1(-14.95)	(4-)8.47(-11.4)	(7-)15.33(-27)	(6-)13.89(-28.35)
**Length of middle stem leaf (mm)**	LL2	(18-)29.91(-45.85)	(24.05-)37.87(-56)	(19.4-)41.17(-69.5)	(14.25-)25.12(-38.75)	(15.9-)28(-41.25)	(21.3-)33.58(-52.7)	(27-)38.24(-55.95)	(26.2-)35.94(-50.6)	(24-)42.22(-66)	(24-)43.46(-72)
Max. width of middle stem leaf (mm)	WLmax2	(3.5-)6.85(-11)	(4.5-)7.82(-11.98)	(3.5-)7.53(-12)	(3-)6.07(-10)	(4-)6.56(-10)	(5-)7.9(-12.43)	(2-)2.96(-4)	(1.9-)2.53(-4)	(4.35-)8.09(-14)	(4-)7.5(-14)
**Max. depth of teeth on middle stem leaf margin (mm)**	DT2	(1-)1.63(-3)	(1-)1.56(-2.95)	(0.5-)1.85(-5)	(1-)2.73(-5)	(1.5-)3.01(-5.05)	(1-)1.85(-3)	**(0-)0.27(-1)**	**(0-)0.06(-0.26)**	(0.5-)1.24(-2.5)	(0.2-)1.18(-2.5)
Number of the teeth on one side of the middle stem leaf margin	NT2	(4-)5.42(-7)	(3-)4.71(-6)	(3-)4.66(-6)	(4-)6.16(-8)	(4.95-)6.2(-8)	(3-)5.46(-7.85)	(0-)1.48(-4)	(0-)0.71(-3.2)	(3-)4.67(-7)	(2-)4.55(-7)
Lenght from the widest part of the middle stem leaf to its tip (mm)	LLw2	(7.15-)15.27(-26.85)	(10-)17.18(-27)	(8.1-)18.14(-29.3)	(7-)12.91(-20)	(7.9-)14.88(-23.2)	(10.15-)17.67(-28.4)	(7.05-)14.36(-23)	(7.8-)11.65(-17.6)	(10-)18.6(-32)	(9-)18.8(-32)
Length of upper stem leaf (mm)	LL3	(12-)21.01(-33)	(14.05-)25.03(-38.95)	(15.7-)29.6(-55.1)	(9.25-)16.55(-25)	(10-)18.5(-28.3)	(14.3-)23.38(-33.85)	(15-)24.82(-42.65)	(18.8-)28.47(-41.4)	(11-)21.21(-36)	(13-)25.17(-43)
Max. width of upper stem leaf (mm)	WLmax3	(2.5-)5.04(-9)	(3-)5.6(-9.95)	(2-)5.16(-9.9)	(2-)4.52(-9)	(2.48-)4.8(-9.1)	(3-)5.75(-9.85)	(1-)1.83(-3)	(1.4-)2.18(-3.3)	(2-)4.08(-8)	(1.5-)4.37(-8.68)
**Max. depth of teeth on upper stem leaf margin (mm)**	DT3	(0.5-)1.34(-2.5)	(0.5-)1.23(-2)	(0.2-)1.07(-2.45)	(0.5-)1.87(-4)	(1-)2.08(-4.53)	(0.5-)1.27(-2)	**(0-)0.15(-0.5)**	**(0-)0.05(-0.26)**	(0.2-)0.78(-1.5)	(0.2-)0.75(-1.5)
Number of the teeth on one side of the upper stem leaf margin	NT3	(3-)4.53(-6)	(2-)3.48(-5)	(2-)3.91(-5.3)	(3-)4.7(-6)	(3-)4.6(-6)	(2.15-)4.25(-7)	(0-)0.77(-3)	(0-)0.29(-2)	(2-)3.45(-5)	(1-)3.32(-5)
Lenght from the widest part of the upper stem leaf to its tip (mm)	LLw3	(5-)12.03(-23)	(8.05-)13.61(-19.95)	(6.4-)14.31(-25.3)	(5-)9.83(-17.75)	(5-)11.3(-19.05)	(9-)14.79(-23.85)	(5-)8.51(-15)	(4-)11.29(-28.6)	(4-)10.42(-20)	(5-)11.58(-21)
**Number of primary teeth bearing secondary teeth on lower stem leaf margin**	NTF1	(0-)0.27(-3)	(0-)0.21(-1)	(0-)1.06(-4.3)	**(0-)3.73(-7)**	**(0-)3.63(-6.05)**	(0-)1.17(-4)	0	0	x	x
**Number of primary teeth bearing secondary teeth on middle stem leaf margin**	NTF2	(0-)0.03(-0)	0	(0-)0.23(-1.3)	**(0-)1.17(-2)**	**(0.95-)1.5(-2.05)**	(0-)0.33(-1)	0	0	x	x
**Number of primary teeth bearing secondary teeth on upper stem leaf margin**	NTF3	(0-)0.04(-0)	0	(0-)0.09(-0.3)	**(0-)1.47(-4)**	**(0-)1.68(-4)**	(0-)0.04(-0)	0	0	x	x
**Number of stem leaves in the upper half of the leafy part of stem**	NL	(4-)10.08(-19)	(6.05-)13.31(-23)	(4-)8.54(-14)	**(5-)10.1(-17)**	**(4-)6.93(-11.05)**	(5.15-)12.29(-18)	(4-)7.13(-10.95)	(4-)5.77(-7.4)	(9-)19.64(-31)	(6-)13.45(-25.35)
Height of the leafy part of the stem (mm)	Hstem	(150-)278(-480)	(265.25-)487(-718)	(149-)335(-495)	(100-)271(-453.75)	(180-)294(-415.5)	(263-)403(-529.25)	(125.25-)220(-340)	(94-)190(-288)	(273.5-)485(-746.5)	(140-)311(-497)
Number of trichomes on the upper surface of the middle stem leaf per area 0.5 x 0.5 mm^2	NrTrL	(4-)6.1(-9)	(4-)6(-8.95)	(4-)5.66(-8)	(5-)7.81(-11.75)	(4-)6.05(-8)	(4-)5.63(-7.85)	(4-)5.51(-7)	(4-)5.41(-8)	(4-)6.13(-9)	(3-)5.23(-8)

Characters/species	Character abbreviation	*E. carniolicum*	*E. vitekii*	*E. carniolicum* x *E. vitekii*	*E. kuemmerlei*	*E. pectinatum*	population from Mt. Ovčar	*E. croaticum* (HR, BA)	*E. croaticum* auct. (XK)	*E. odoratum*	*E. witmannii*
		(5%-)MEAN(-95%)	(5%-)MEAN(-95%)	(5%-)MEAN(-95%)	(5%-)MEAN(-95%)	(5%-)MEAN(-95%)	(5%-)MEAN(-95%)	(5%-)MEAN(-95%)	(5%-)MEAN(-95%)	(5%-)MEAN(-95%)	(5%-)MEAN(-95%)
**Max. length of the trichome ray on the upper surface of the middle stem leaf (µm) measured for ≥3-fid trichomes**	LTrRay	(0.14-)0.18(-0.23)	**(0.17-)0.21(-0.25)**	(0.16-)0.19(-0.23)	(0.15-)0.18(-0.22)	(0.15-)0.19(-0.22)	(0.17-)0.21(-0.23)	x	x	(0.18-)0.23(-0.28)	(0.16-)0.2(-0.26)
Max. length of the trichome ray on the upper surface of the middle stem leaf (µm) measured for 2-fid trichomes	LTrRay2	x	x	x	x	x	x	(0.24-)0.31(-0.37)	(0.23-)0.27(-0.33)	x	x
Ratio Length of lower stem leaf (including petiole if present)/Length of middle stem leaf	LL1/LL2	(0.72-)1.04(-1.44)	(0.88-)1.16(-1.44)	(0.75-)1.01(-1.27)	(0.8-)1.22(-1.8)	(0.86-)1.23(-1.64)	(0.8-)1.1(-1.45)	(0.85-)1.12(-1.5)	(0.81-)1.19(-1.68)	(0.78-)1.14(-1.6)	(0.72-)1.08(-1.49)
Ratio Length of middle stem leaf/Length of upper stem leaf	LL2/LL3	(1.03-)1.46(-2.15)	(1.07-)1.57(-2.17)	(1.05-)1.47(-2.48)	(1.09-)1.57(-2.27)	(1.08-)1.54(-2.2)	(1.06-)1.48(-2.13)	(1.13-)1.61(-2.18)	(0.78-)1.31(-1.68)	(1.31-)2.10(-3.27)	(1.16-)1.81(-2.67)
Ratio Number of stem leaves in the upper half of the leafy part of stem/Height of the leafy part of the stem	NL/HStem	(0.02-)0.04(-0.07)	(0.02-)0.03(-0.04)	(0.01-)0.03(-0.05)	(0.02-)0.04(-0.08)	(0.01-)0.02(-0.04)	(0.02-)0.03(-0.05)	(0.02-)0.03(-0.05)	(0.02-)0.03(-0.05)	(0.02-)0.04(-0.07)	(0.02-)0.05(-0.09)
**Ratio Length of lower stem leaf (including petiole if present)/Height of the leafy part of the stem**	LL1/Hstem	(0.07-)0.12(-0.19)	(0.05-)0.1(-0.18)	(0.08-)0.13(-0.22)	(0.06-)0.13(-0.27)	(0.07-)0.12(-0.2)	(0.05-)0.09(-0.18)	**(0.12-)0.21(-0.33)**	**(0.15-)0.25(-0.42)**	(0.06-)0.1(-0.16)	(0.08-)0.17(-0.32)
**Ratio Length of middle stem leaf/Height of the leafy part of the stem**	LL2/HStem	(0.07-)0.12(-0.2)	(0.05-)0.09(-0.15)	(0.08-)0.13(-0.22)	(0.05-)0.11(-0.23)	(0.07-)0.1(-0.15)	(0.05-)0.09(-0.15)	**(0.09-)0.19(-0.29)**	**(0.12-)0.21(-0.36)**	(0.05-)0.09(-0.15)	(0.08-)0.16(-0.29)
**Ratio Length of upper stem leaf/Height of the leafy part of the stem**	LL3/HStem	(0.04-)0.08(-0.15)	(0.03-)0.06(-0.11)	(0.05-)0.09(-0.15)	(0.03-)0.07(-0.16)	(0.04-)0.06(-0.09)	(0.04-)0.06(-0.11)	**(0.06-)0.12(-0.22)**	**(0.08-)0.17(-0.3)**	(0.02-)0.05(-0.09)	(0.04-)0.09(-0.2)
**Ratio Length/Max. width of lower stem leaf**	LL1/WLmax1	(3.34-)5.18(-8)	(3.31-)5.61(-7.88)	(3.92-)5.59(-7.42)	(3.23-)5.2(-7.88)	(3.79-)5.94(-10.82)	(3.03-)4.47(-5.65)	**(7.52-)13(-20.74)**	**(8.46-)13.34(-24.2)**	(4.29-)6.34(-9.17)	(4.58-)7.51(-11.54)
**Ratio Length/Max. width of middle stem leaf**	LL2/WLmax2	(3.18-)4.53(-6.2)	(3.67-)5.07(-7.6)	(3.49-)5.67(-8.01)	(2.8-)4.43(-7.36)	(2.79-)4.35(-6.3)	(3.27-)4.4(-5.6)	**(8.61-)13.53(-20.97)**	**(9.35-)14.93(-22.88)**	(3.71-)5.44(-7.68)	(4.24-)6.09(-8.73)
**Ratio Length/Max. width of upper stem leaf**	LL3/WLmax3	(3-)4.46(-6.48)	(2.78-)4.83(-7.74)	(3.45-)6.07(-9.66)	(2.29-)4.12(-6.63)	(2.79-)4.18(-6.03)	(3.01-)4.44(-6.8)	**(8.51-)14.25(-21.95)**	**(8-)13.97(-19.1)**	(3.5-)5.65(-8.5)	(4-)6.26(-9.54)
Ratio Length from the widest part of the lower stem leaf to its tip/Length of lower stem leaf	LLw1/LL1	(0.24-)0.39(-0.59)	(0.23-)0.4(-0.67)	(0.2-)0.37(-0.58)	(0.22-)0.41(-0.67)	(0.23-)0.44(-0.68)	(0.22-)0.35(-0.48)	(0.15-)0.24(-0.36)	(0.12-)0.21(-0.33)	(0.18-)0.33(-0.49)	(0.15-)0.3(-0.48)
Ratio Length from the widest part of the middle stem leaf to its tip/Length of middle stem leaf	LLw2/LL2	(0.33-)0.51(-0.69)	(0.28-)0.45(-0.64)	(0.26-)0.46(-0.67)	(0.33-)0.52(-0.78)	(0.33-)0.53(-0.73)	(0.38-)0.54(-0.68)	(0.22-)0.38(-0.57)	(0.22-)0.33(-0.49)	(0.3-)0.44(-0.6)	(0.27-)0.43(-0.58)
Ratio Length from the widest part of the upper stem leaf to its tip/Length of upper stem leaf	LLw3/LL3	(0.38-)0.56(-0.76)	(0.39-)0.56(-0.73)	(0.37-)0.49(-0.68)	(0.38-)0.59(-0.81)	(0.37-)0.61(-0.8)	(0.46-)0.63(-0.77)	(0.18-)0.36(-0.56)	(0.21-)0.38(-0.64)	(0.31-)0.49(-0.69)	(0.28-)0.46(-0.67)
**Ratio Number of teeth on one side of the lower stem leaf margin/Length of lower stem leaf**	NT1/LL1	**(0.1-)0.2(-0.33)**	(0.08-)0.12(-0.19)	(0.07-)0.15(-0.27)	**(0.12-)0.25(-0.38)**	**(0.12-)0.22(-0.36)**	(0.11-)0.18(-0.3)	(0-)0.06(-0.1)	(0-)0.05(-0.12)	(0.04-)0.1(-0.16)	(0.04-)0.12(-0.2)
**Ratio Number of teeth on one side of the middle stem leaf margin/Length of middle stem leaf**	NT2/LL2	**(0.1-)0.2(-0.33)**	(0.09-)0.13(-0.18)	(0.06-)0.13(-0.18)	**(0.14-)0.27(-0.38)**	**(0.13-)0.25(-0.4)**	(0.1-)0.18(-0.27)	**(0-)0.04(-0.1)**	**(0-)0.02(-0.11)**	(0.06-)0.12(-0.19)	(0.06-)0.11(-0.19)
**Ratio Number of teeth on one side of the upper stem leaf margin/Length of upper stem leaf**	NT3/LL3	**(0.13-)0.23(-0.37)**	(0.07-)0.15(-0.21)	(0.07-)0.15(-0.25)	**(0.18-)0.3(-0.5)**	**(0.15-)0.27(-0.45)**	(0.12-)0.19(-0.28)	**(0-)0.04(-0.13)**	**(0-)0.01(-0.05)**	(0.07-)0.18(-0.31)	(0.06-)0.15(-0.27)
Ratio Max. depth of teeth on lower stem leaf margin/Max. width of lower stem leaf	DT1/WLmax1	(0.17-)0.34(-0.5)	(0.09-)0.28(-0.45)	(0.13-)0.31(-0.5)	**(0.33-)0.45(-0.6)**	**(0.33-)0.48(-0.6)**	(0.18-)0.31(-0.49)	(0-)0.16(-0.33)	(0-)0.1(-0.24)	(0.07-)0.2(-0.36)	(0.06-)0.22(-0.42)
**Ratio Max. depth of teeth on middle stem leaf margin/Max. width of middle stem leaf**	DT2/WLmax2	(0.12-)0.24(-0.35)	(0.11-)0.21(-0.32)	(0.08-)0.24(-0.44)	**(0.29-)0.45(-0.59)**	**(0.33-)0.45(-0.59)**	(0.15-)0.24(-0.33)	(0-)0.08(-0.3)	(0-)0.02(-0.11)	(0.07-)0.16(-0.25)	(0.05-)0.16(-0.27)
**Ratio Max. depth of teeth on upper stem leaf margin/Max. width of upper stem leaf**	DT3/WLmax3	(0.13-)0.27(-0.4)	(0.11-)0.23(-0.44)	(0.09-)0.2(-0.4)	**(0.25-)0.41(-0.6)**	**(0.33-)0.43(-0.6)**	(0.13-)0.23(-0.33)	(0-)0.07(-0.25)	(0-)0.02(-0.09)	(0.07-)0.2(-0.33)	(0.06-)0.17(-0.33)
**Ratio Number of primary teeth bearing secondary teeth on lower stem leaf margin/Number of teeth on one side of the lower stem leaf margin**	NTF1/NT1	(0-)0.05(-0.44)	(0-)0.04(-0.2)	(0-)0.18(-0.74)	**(0-)0.54(-0.83)**	**(0-)0.55(-0.84)**	(0-)0.18(-0.78)	0	0	x	x
**Ratio Number of primary teeth bearing secondary teeth on middle stem leaf margin/Number of teeth on one side of the middle stem leaf margin**	NTF2/NT2	(0-)0.01(-0)	0	(0-)0.11(-0.69)	**(0-)0.53(-0.83)**	**(0.19-)0.6(-0.88)**	(0-)0.17(-0.75)	0	0	x	x
**Ratio Number of primary teeth bearing secondary teeth on upper stem leaf margin/Number of teeth on one side of the upper stem leaf margin**	NTF3/NT3	(0-)0.01(-0)	0	(0-)0.02(-0.08)	**(0-)0.29(-0.8)**	**(0-)0.36(-0.81)**	(0-)0.01(-0)	0	0	x	x

x, morphological characters not present/measured in these species.

Bačák R, et al. 2026. Understanding early stages of speciation: allopatric divergence, introgression and chromosomal dynamics in the *Erysimum odoratum* species complex. Molecular Phylogenetics and Evolution 214: 108481. https://doi.org/10.1016/j.ympev.2025.108481
